# Molecular diversity and phenotypic pleiotropy of ancient genomic regulatory loci derived from human endogenous retrovirus type H (HERVH) promoter LTR7 and HERVK promoter LTR5_Hs and their contemporary impacts on pathophysiology of Modern Humans

**DOI:** 10.1007/s00438-022-01954-7

**Published:** 2022-09-19

**Authors:** Gennadi V. Glinsky

**Affiliations:** Institute of Engineering in Medicine, University of California, San Diego, 9500 Gilman Dr. MC 0435, La Jolla, San Diego, CA 92093-0435 USA

**Keywords:** Human endogenous retrovirus type H (HERVH), Human endogenous retrovirus type K (HERVK), LTR7 subfamilies, LTR5_Hs, Retrotransposition, Primate evolution, Mammalian offspring survival genes, Human embryogenesis, Human spermatogenesis, Synaptic transmission, Protein–protein interactions at synapses, Oligospermia, Azoospermia, Neoplasm metastasis, SARS-CoV-2 coronavirus, COVID-19 pandemic

## Abstract

Timelines of population-level effects of viruses on humans varied from the evolutionary scale of million years to contemporary spread of viral infections. Correspondingly, these events are exemplified by: (i) emergence of human endogenous retroviruses (HERVs) from ancient germline infections leading to stable integration of viral genomes into human chromosomes; and (ii) wide-spread viral infections reaching a global pandemic state such as the COVID-19 pandemic. Despite significant efforts, understanding of HERV’s roles in governance of genomic regulatory networks, their impacts on primate evolution and development of human-specific physiological and pathological phenotypic traits remains limited. Remarkably, present analyses revealed that expression of a dominant majority of genes (1696 of 1944 genes; 87%) constituting high-confidence down-steam regulatory targets of defined HERV loci was significantly altered in cells infected with the SARS-CoV-2 coronavirus, a pathogen causing the global COVID-19 pandemic. This study focused on defined sub-sets of DNA sequences derived from HERVs that are expressed at specific stages of human preimplantation embryogenesis and exert regulatory actions essential for self-renewal and pluripotency. Evolutionary histories of LTR7/HERVH and LTR5_Hs/HERVK were charted based on evidence of the earliest presence and expansion of highly conserved (HC) LTR sequences. Sequence conservation analyses of most recent releases 17 primate species’ genomes revealed that LTR7/HERVH have entered germlines of primates in Africa after the separation of the New World Monkey lineage, while LTR5_Hs/HERVK successfully colonized primates’ germlines after the segregation of Gibbons’ species. Subsequently, both LTR7 and LTR5_Hs undergo a marked ~ fourfold–fivefold expansion in genomes of Great Apes. Timelines of quantitative expansion of both LTR7 and LTR5_Hs loci during evolution of Great Apes appear to replicate the consensus evolutionary sequence of increasing cognitive and behavioral complexities of non-human primates, which seems particularly striking for LTR7 loci and 11 distinct LTR7 subfamilies. Consistent with previous reports, identified in this study, 351 human-specific (HS) insertions of LTR7 (175 loci) and LTR5_Hs (176 loci) regulatory sequences have been linked to genes implicated in establishment and maintenance of naïve and primed pluripotent states and preimplantation embryogenesis phenotypes. Unexpectedly, HS-LTRs manifest regulatory connectivity to genes encoding markers of 12 distinct cells’ populations of fetal gonads, as well as genes implicated in physiology and pathology of human spermatogenesis, including Y-linked spermatogenic failure, oligo- and azoospermia. Granular interrogations of genes linked with 11 distinct LTR7 subfamilies revealed that mammalian offspring survival (MOS) genes seem to remain one of consistent regulatory targets throughout ~ 30 MYA of the divergent evolution of LTR7 loci. Differential GSEA of MOS versus non-MOS genes identified clearly discernable dominant enrichment patterns of phenotypic traits affected by MOS genes linked with LTR7 (562 MOS genes) and LTR5_Hs (126 MOS genes) regulatory loci across the large panel of genomics and proteomics databases reflecting a broad spectrum of human physiological and pathological traits. GSEA of LTR7-linked MOS genes identified more than 2200 significantly enriched records of human common and rare diseases and gene signatures of 466 significantly enriched records of Human Phenotype Ontology traits, including Autosomal Dominant (92 genes) and Autosomal Recessive (93 genes) Inheritance. LTR7 regulatory elements appear linked with genes implicated in functional and morphological features of central nervous system, including synaptic transmission and protein–protein interactions at synapses, as well as gene signatures differentially regulated in cells of distinct neurodevelopmental stages and morphologically diverse cell types residing and functioning in human brain. These include Neural Stem/Precursor cells, Radial Glia cells, Bergman Glia cells, Pyramidal cells, Tanycytes, Immature neurons, Interneurons, Trigeminal neurons, GABAergic neurons, and Glutamatergic neurons. GSEA of LTR7-linked genes identified significantly enriched gene sets encoding markers of more than 80 specialized types of neurons and markers of 521 human brain regions, most prominently, subiculum and dentate gyrus. Identification and characterization of 1944 genes comprising high-confidence down-steam regulatory targets of LTR7 and/or LTR5_Hs loci validated and extended these observations by documenting marked enrichments for genes implicated in neoplasm metastasis, intellectual disability, autism, multiple cancer types, Alzheimer’s, schizophrenia, and other brain disorders. Overall, genes representing down-stream regulatory targets of ancient retroviral LTRs exert the apparently cooperative and exceedingly broad phenotypic impacts on human physiology and pathology. This is exemplified by altered expression of 93% high-confidence LTR targets in cells infected by contemporary viruses, revealing a convergence of virus-inflicted aberrations on genomic regulatory circuitry governed by ancient retroviral LTR elements and interference with human cells’ differentiation programs.

**Supplementary Information:**

The online version contains supplementary material available at 10.1007/s00438-022-01954-7.

## Introduction

Viruses possess unique abilities to affect human population’s phenotypic traits on a markedly broad timescale ranging from the evolutionary span of millions’ years to contemporary wide-spread pandemic infections. Ancient germline infections leading to stable integration of multiple copies of viral genomes into human chromosomes caused the emergence of human endogenous retroviruses (HERVs) that evolved into intrinsic regulatory elements of genomes of Modern Humans. Contemporary wide-spread viral infections reaching a global pandemic state such as the COVID-19 pandemic constitute a present day example of virus–host interactions affecting humans’ population. It is not known whether these markedly distinct virus infections-associated phenomena causing population-scale effects on Modern Humans may share the common genomic underpinning. One of the approaches facilitating the resolution of this conundrum may represent comparative analyses of gene expression networks affected by HERV-derived regulatory elements and contemporary viral infections.

In the human genome, there are thousands of genomic loci origin of which could be traced to hundreds of distinct HERV families and subfamilies (Kojima 2018; Vargiu et al. 2016). Sequences derived from LTR7/HERVH retroviruses are among the most abundant and extensively investigated genomic regulatory elements that were originated from various HERV families. LTR7/HERVH insertions originated from a gamma retrovirus that presumably infected primates approximately 40 million years ago (MYA) and colonized the genome of the common ancestor of Apes, Old World Monkeys, and New World Monkeys (Goodchild et al. 1993; Mager and Freeman 1995).

Substantial uncertainties remain in the estimates of species divergence timelines during primate evolution, often reporting different timelines of species segregation. The divergence of human and chimpanzee ancestors most often dates back to approximately 6,5–7,5 MYA (Langergraber et al. 2012; Amster et al. 2016). Moorjani et al. (2016) demonstrate that genomic divergence events in primate evolution are most reliably dated using CpG transitions. Using mutations accumulated at CpG sites for calculations of species split time estimates, they estimated the human and chimpanzee divergence time at 12.1 MYA, while the human and gorilla divergence time was estimated at 15.1 MYA (Moorjani et al. 2016). Using mutation rates calculated by comparing parents to offspring, Prüfer et al. (2017) estimated the human–chimpanzee divergence time at 13 MYA rather than 6.5 MYA. Whole genome molecular dating analyses indicate that the gibbon lineage (Carbone et al. 2014) diverged from that of great apes around 16.8 MYA (95% confidence interval: 15.9–17.6 MYA; given a divergence of 29 MYA from Old World monkeys). Divergence time from the other hominids (gorillas, chimpanzees, and humans) of the orangutans, the only surviving species of the subfamily Ponginae, is estimated between 15.7 and 19.3 MYA. Estimates of the divergence time between species are based on estimated mutation rates of the species’ genomes. They remain controversial, in part, because calibrations of the mutation rate in humans and other species may be significantly different depending on the utilized methodology and other confounders (Gibb and Hills 2013; Harris 2015). For example, mutation rates estimated by calibrating with dated fossils were determined as 1 × 10–9 per base per year, or ~ 2.5 × 10–8 per base per generation (Prüfer et al. 2014), while mutation rates estimated by comparing parents to offspring are about two-fold slower (0.5 × 10–9 per base per year, or 1.25 × 10–8 per base per generation). A recent report delineating an ancestral recombination graph of human, Neanderthal, and Denisovan genomes seems in agreement with setting the estimated human–chimpanzee divergence time at 13 MYA (Schaefer et al. 2021).

In this study, defined sub-sets of DNA sequences, which were derived from HERV’s families that are expressed at specific stages of human preimplantation embryogenesis and exert regulatory actions essential for establishment and maintenance of self-renewal and pluripotency phenotypes, were analyzed. Evolutionary histories and putative phenotypic impacts of genomic regulatory loci derived from infections of LTR7/HERVH and LTR5_Hs/HERVK retroviruses and residing at thousands fixed non-polymorphic locations in genomes of Modern Humans were elucidated. Many non-human primate genomes generated to date have been ''humanized'' as a result of the reliance on guidance by the reference human genome on account of their many sequencing gaps. These humanizing effects were resolved in the most recent contributions by generating and assembling long-read genomes of non-human primates. For example, Kronenberg et al. (2018) generated and assembled long-read genomes of a chimpanzee, an orangutan, and two humans and compared them with a previously generated gorilla genome to unambiguously identify genomic structural variation specific to humans and particular ape lineages. Therefore, it is essential to investigate the patterns of evolutionary conservations of genomic regulatory loci derived from HERVs employing the most recent releases of non-human primates’ reference genomes.

More than 3,000 DNA sequences derived from insertions of the human endogenous retrovirus type H (HERVH) are scattered across human genome at fixed non-polymorphic locations (Thomas et al. 2018).

The long terminal repeats designated LTR7 harbor the promoter sequence of the HERVH and the HERVH/LTR7 family has been extensively investigated in the context of its regulatory functions and locus-specific differential expression in human preimplantation embryogenesis, human embryonic and pluripotent stem cells (Fort et al. 2014; Gemmell et al. 2015; Glinsky et al. 2018; Göke et al. 2015; Izsvák et al. 2016; Kelley and Rinn 2012; Loewer et al. 2010; Lu et al. 2014; Ohnuki et al. 2014; Pontis et al. 2019; Römer et al. 2017; Santoni et al. 2012; Takahashi et al. 2021; Theunissen et al. 2016; Wang et al. 2014; Zhang et al. 2019). It has been reported that HERVH/LTR7s harbor binding sites for master pluripotency transcription factors OCT4, NANOG, SP1, and SOX2 which bind HERVH/LTRs and activate their expression (Glinsky 2015; Göke et al. 2015; Ito et al. 2017; Kelley and Rinn, 2012; Kunarso et al. 2010; Ohnuki et al. 2014; Pontis et al. 2019; Santoni et al. 2012).

Most of the previous studies have considered HERVH/LTR7 insertions in human genome as functionally homogenous genomic regulatory elements (Bao et al. 2015; Gemmell et al. 2019; Göke et al. 2015; Izsvák et al. 2016; Lu et al. 2014; Storer et al. 2021; Wang et al. 2014; Zhang et al. 2019) and regarded the entire family of > 3000 insertion sites as one monophyletic entity. In contrast, application of a ‘phyloregulatory’ approach that integrates chromatin state features as well as regulatory and expression profiling genomics data to a phylogenetic analysis of LTR7 sequences facilitated discovery of new insights into the remarkable diversity of origin, evolution, and transcriptional activities of the HERVH/LTR7 family (Carter et al. 2022). This granular interrogation of fine molecular structures of LTR7 elements and their evolutionary history has revealed striking genetic and regulatory distinctions among LTR7 elements by demonstrating that LTR7 sequences represent a polyphyletic group composed of at least eight monophyletic subfamilies (Carter et al. 2022). Collectively, these findings indicate that the HERVH/LTR7 family underwent the extensive diversification of LTR sequences during primate evolution through a combination of point mutations, indels, and recombination events facilitating the gain, loss, and exchange of multiple cis-regulatory modules. These processes are likely underlying the apparent functional partitioning of LTR7 transcription regulatory activities during primates’ preimplantation embryogenesis and maintenance of a pluripotency phenotype (Carter et al. 2022).

Human endogenous retrovirus type K [HERVK (HTML-2)] is the most recently endogenized primate-specific retrovirus and all human-specific and human-polymorphic HERVK insertions are associated with a specific LTR subtype designated LTR5_Hs (Hanke et al. 2016; Subramanian et al. 2011). It has been reported that LTR5_Hs/HERVK manifests transcriptional and biological activities in human preimplantation embryos and in naïve hESCs (Grow et al. 2015). Notably, LTR5_Hs elements acquire enhancer-like chromatin state signatures concomitantly with transcriptional reactivation of HERVK (Grow et al. 2015). Genome-wide CRISPR-guided activation and interference experiments targeting LTR5_Hs elements demonstrated global long-range effects on expression of human genes consistent with postulated functions of LTR5_Hs as distal enhancers (Fuentes et al. 2018).

Analyses of sequenced individual human genomes, including genomes from the 1000 Genomes Project and the Human Genome Diversity Project, consistently demonstrated insertional polymorphism of HERVK retroviruses (Autio et al. 2021; Hughes and Coffin 2004; Barbulescu et al. 1999; Belshaw et al. 2005; Shin et al. 2013; Turner et al. 2001; Wildschutte et al. 2016). There are at least 36 non-reference polymorphic HERVK proviruses with insertion frequencies ranging from < 0.0005 to > 0.75 that varied by distinct human populations (Wildschutte et al. 2016). In contrast, no polymorphic HERVH insertions have been found in the human genome (Thomas et al. 2018).

In the present study, detailed analyses of evolutionary conservation patterns of eleven LTR7 subfamilies were carried out to understand when HERVH/LTR7 family has infiltrated the primates’ germline at different evolutionary time points and how they have achieved quantitatively and qualitatively various levels of genomic amplification in distinct primate species during evolution. To highlight connectivity patterns between the LTR7 structural diversity and the phenotypic pleiotropy of putative regulatory functions of LTR7 elements, the GREAT algorithm was employed to identify and characterize LTR7-linked genes. Comprehensive Gene Set Enrichment Analyses (GSEA) of LTR7-linked genes were performed to infer potential phenotypic impacts of LTR7 regulatory networks and findings were juxtaposed to results of analyses of LTR5_Hs elements. Observations reported in this contribution demonstrate that despite markedly distinct evolutionary histories of retroviral LTRs, genes representing down-stream regulatory targets of LTR7 and LTR5_Hs elements exert the apparently cooperative and exceedingly broad phenotypic impacts on human physiology and pathology. Among striking examples of the contemporary significance of characterized herein genomic regulatory networks governed by ancient retroviral LTR elements are evidence that SARS-CoV-2 infection alters expression of a dominant majority (1696 of 1944 genes; 87%) of high-confidence LTR-target genes. Collectively, these findings implicate the interference with cellular differentiation programs in multiple types of human cells and tissues as one of molecular mechanisms of clinical pathogenesis of the COVID-19 pandemic.

## Methods

### Data source and analytical protocols

A total of 3354 LTR7 loci and 606 LTR5_Hs loci residing at fixed non-polymorphic locations in genomes of Modern Humans (hg38 human reference genome database) analyzed in this study were reported previously (Carter et al. 2022; Fuentes et al. 2018). LTR loci residing in the human genome has been considered highly conserved in the corresponding genome of non-human primates (NHP) only if the following two requirements are met: (1) during the direct LiftOver test (https://genome.ucsc.edu/cgi-bin/hgLiftOver), the human sequence mapped in the NHP genome to the single orthologous locus with at least 95% sequence identity threshold; (2) during the reciprocal LiftOver test, the NHP sequence identified in the direct LiftOver test remapped with at least 95% sequence identity threshold to the exactly same human orthologous sequence which was queried during the direct LiftOver test.

Solely publicly available datasets and resources were used in this contribution. The significance of the differences in the expected and observed numbers of events was calculated using two-tailed Fisher’s exact test. Additional proximity placement enrichment and gene set enrichment tests were performed for individual classes of regulatory sequences taking into account the position and size in bp of corresponding genomic regions, size distributions in human cells of topologically associating domains, distances to putative regulatory targets, bona fide regulatory targets identified in targeted genetic interference and/or epigenetic silencing experiments, details of methodological and analytical approaches of which were reported previously (Barakat et al. 2018; Fuentes et al. 2018; Glinsky 2015; 2016a, b; 2018; 2019; 2020a, b, c, 2021; Guffanti et al. 2018; McLean et al. 2010; 2011; Pontis et al. 2019; Wang et al. 2014).

### Gene set enrichment and genome-wide proximity placement analyses

Gene set enrichment analyses (GSEA) were carried out using the Enrichr bioinformatics platform, which enables the interrogation of nearly 200,000 gene sets from more than 100 gene set libraries. The Enrichr API (January 2018 through March 2022 releases) (Chen et al. 2013; Kuleshov et al. 2016; Xie et al. 2021) was used to test genes linked to regulatory LTRs of interest for significant enrichment in numerous functional categories. When technically feasible, larger sets of genes comprising several thousand entries were analyzed. Regulatory connectivity maps between LTRs and coding genes and additional functional enrichment analyses were performed with the GREAT algorithm (McLean et al. 2010; 2011) applying default settings at differing maximum extension thresholds as previously reported (Glinsky 2020a, b, c; 2021). The reproducibility of the results was validated by implementing two releases of the GREAT algorithm: GREAT version 3.0.0 (2/15/2015 to 08/18/2019) and GREAT version 4.0.4 (08/19/2019) as well as two releases of the human genome reference database (hg19 and hg38). The GREAT algorithm allows investigators to identify and annotate the genome-wide connectivity networks of user-defined distal regulatory loci and their putative target genes. Concurrently, the GREAT algorithm performs functional annotations and analyses of statistical enrichment of annotations of identified genes, thus enabling the inference of potential biological significance of interrogated genomic regulatory networks (GRNs). Genome-wide Proximity Placement Analysis (GPPA) of distinct genomic features co-localizing with LTRs and human-specific regulatory sequences (HSRS) was carried out as described previously and initially implemented for interrogation of human-specific transcription factor-binding sites and other candidate HSRS (Glinsky et al. 2018; 2019; Glinsky 2015, 2016a, 2016b, 2017, 2018, 2019, 2020a, 2020b, 2020c, 2021; Guffanti et al. 2018).

Targeted differential GSEA were employed to infer the relative contributions of distinct sub-sets of genes on phenotypes of interest. In brief, to gain insights into biological effects of LTRs and infer potential mechanisms of biological activities, multiple sets of differentially expressed genes (DEGs) and/or coding genes representing putative regulatory targets of LTRs were identified. These gene sets comprising from dozens to several thousand individual genetic loci were defined at multiple significance levels of corresponding statistical metrics and analyzed using differential GSEA applied to ~ 30 genomics and proteomics databases. This approach was successfully implemented for identification and characterization of human-specific regulatory networks governed by human-specific transcription factor-binding sites (Glinsky et al. 2018, 2019, 2016a, 2016b, 2017, 2018, 2019, 2020a, 2020b, 2020c, 2021; Glinsky 2015; Guffanti et al. 2018) and functional enhancer elements (Barakat et al. 2018; Glinsky et al. 2018; Glinsky and Barakat 2019; Glinsky 2015, 2016a, 2016b, 2017, 2018, 2019, 2020a, 2020b, 2020c, 2021), 13,824 genes associated with 59,732 human-specific regulatory sequences (Glinsky 2020a), 8405 genes associated with 35,074 human-specific neuro-regulatory single-nucleotide changes (Glinsky 2020b), different sub-sets of 8384 genes regulated by stem cell-associated retroviral sequences (Glinsky 2021), as well as human genes and medicinal molecules affecting the susceptibility to SARS-CoV-2 coronavirus (Glinsky 2020c).

According to a standard analytical protocol, differential GSEA entail interrogations of specific sets of DEGs comprising LTR-regulated genes using distinct genomic databases, including comprehensive pathway enrichment Gene Ontology (GO) analyses. Upon completion, these analyses were followed by in-depth interrogations of the identified significantly enriched genes employing selected genomic databases deemed most statistically informative during the initial GSEA. In all tables and plots (unless stated otherwise), in addition to the nominal *p* values and adjusted *p* values, the “combined score” calculated by Enrichr software is reported, which is a product of the significance estimate and the magnitude of enrichment (combined score c = log(p) * z, where *p* is the Fisher’s exact test *p* value and z is the z-score deviation from the expected rank).

### Statistical analyses of the publicly available datasets

All statistical analyses of the publicly available genomic datasets, including error rate estimates, background and technical noise measurements and filtering, feature peak calling, feature selection, assignments of genomic coordinates to the corresponding builds of the reference human genome, and data visualization, were performed exactly as reported in the original publications and associated references linked to the corresponding data visualization tracks (http://genome.ucsc.edu/). Any modifications or new elements of statistical analyses are described in the corresponding sections of the Results. Statistical significance of the Pearson’s correlation coefficients was determined using GraphPad Prism version 6.00 software. Both nominal and Bonferroni adjusted *p* values were estimated and considered. The significance of the differences in the numbers of events between the groups was calculated using two-sided Fisher’s exact and Chi-square test, and the significance of the overlap between the events was determined using the hypergeometric distribution test (Tavazoie et al. 1999).

## Results

### LTR family-specific and granular analyses of evolutionary origin, expansion, conservation, and divergence of HERVH promoter LTR7 and HERVK promoter LTR5_Hs during primate evolution

**Fig. 1:**
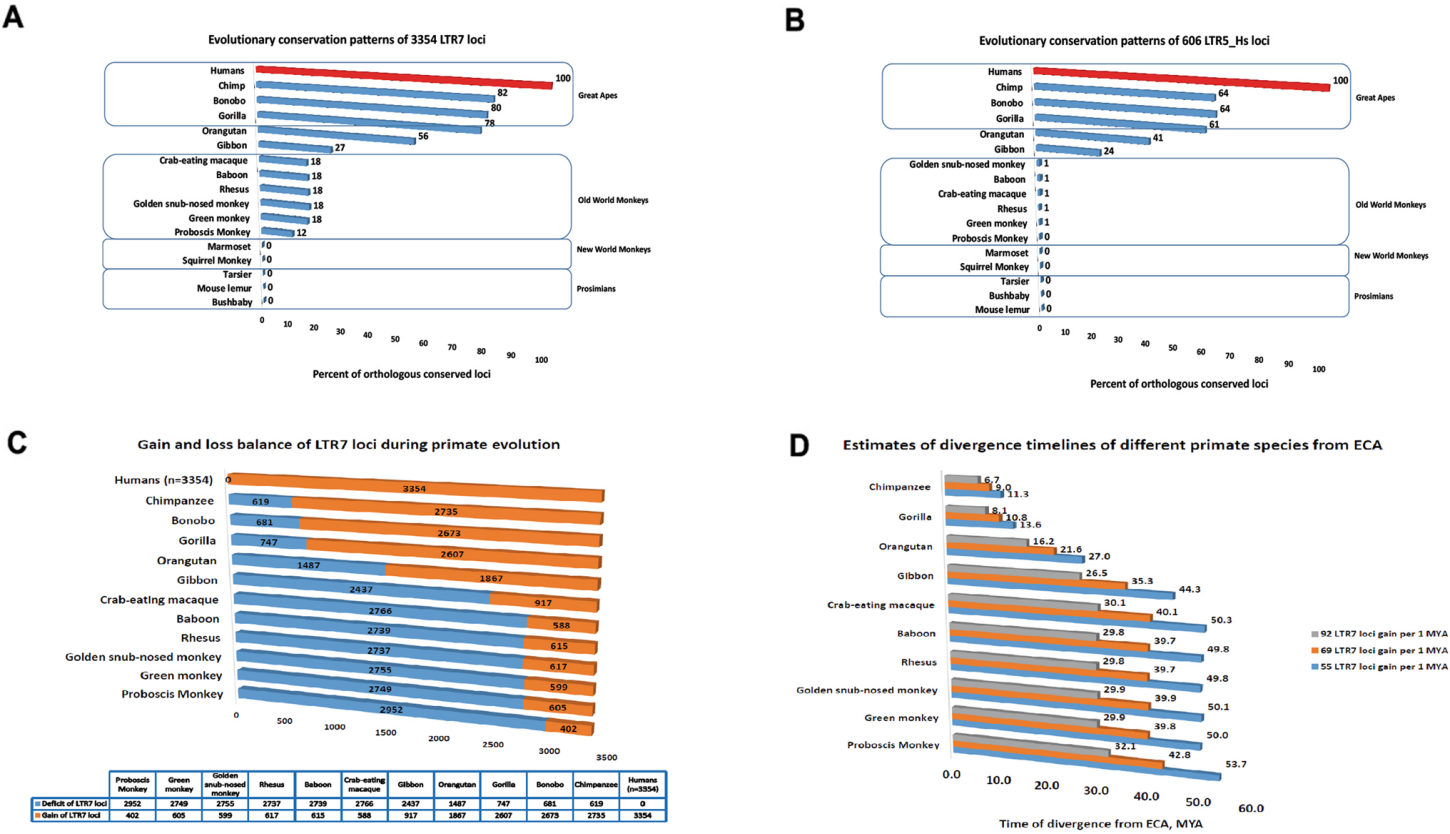
Evolutionary conservation analysis of regulatory LTR7 (**A**) and LTR5_Hs (**B**) loci and a model of species-specific expansion of regulatory LTR7 loci during primate evolution (**C**) and its putative associations with species segregation processes (**D**). The model is presented as a balance of gains and losses of highly conserved orthologous LTR7 loci in genome of each primate species (**C**). Estimates of divergence timelines of different primate species from ECA based on estimated numbers of LTR7 loci acquisition per 1 MYA (**D**)

Precise timelines of transitions of retroviruses from the state of exogenous infection agents to endogenous retroviral sequences integrated into host genomes remain unknown. This information could be inferred from the comparative analyses of highly conserved retrovirus-derived loci in genomes of multiple distinct primate species with known species’ divergence time from the last extinct common ancestor (ECA) during primates’ evolution. To this end, fixed non-polymorphic sequences of 3354 LTR7 loci and 606 LTR5_Hs loci residing in genomes of Modern Humans (hg38 human reference genome database) were retrieved and the number of highly conserved loci in genomes of sixteen non-human primates (NHP) were determined (Fig. 1). Results of these analyses demonstrate that LTR7 and LTR5_Hs loci appear to have clearly distinguishable evolutionary histories. The consistent earliest presence of LTR7 loci could be mapped to genomes of Old World Monkeys (Fig. 1A), suggesting that LTR7/HERVH retroviruses have entered germlines of the primate lineage after the separation of the New World Monkey lineage. Subsequently, endogenous LTR7/HERVH retroviruses appear to undergo a marked 4–fivefold expansion in genomes of Great Apes (Fig. 1A). Similar marked expansion of retroviral LTR7 loci in Great Apes’ genomes has been observed recently during the evolutionary age analyses of LTR7 subfamilies (Carter et al. 2022). While small numbers (~ 1%) of highly conserved LTR5_Hs loci could be traced to genomes of Old World Monkeys (Fig. 1B), the consistent earliest presence of highly conserved LTR5_Hs loci has been observed in the Gibbon’s genome (143 loci; 24% of HC LTR5_Hs loci residing in human genomes). Taken together, these findings suggest that LTR5_Hs/HERVK retroviruses successfully colonized germlines of the primate lineage after the segregation of Gibbons’ species and subsequently underwent a marked expansion in genomes of Great Apes (Fig. 1B).

Interestingly, timeline sequences of quantitative expansion of both LTR7 and LTR5_Hs loci during primate evolution appear to replicate the consensus evolutionary sequence of increasing cognitive and behavioral complexities of NHP (Fig. 1), which seems particularly striking for LTR7 loci (Fig. 1A). This hypothesis was extended further by building a model of a putative species-specific expansion of regulatory LTR7 loci during primate evolution presented for genomes of eleven NHP as relative gains (a number of highly conserved LTR7 loci identified in a genome) and losses (a deficit of highly conserved LT7 loci with regard to human genome) of LTR7 loci vis-a-vis Modern Human’s genome (Fig. 1C). A notable feature of this model is apparently similar numbers of gains and losses of LTR7 loci independently estimated for genomes of five Old World Monkeys’ species, providing a baseline for estimates of numbers of LTR7 loci gains per MYA during primate evolution (Fig. 1D). Based on these estimates tailored to a presumed timeline of Old World Monkeys’ segregation from ECA, a hypothetical model defining species segregation timelines could be built, which reflect putative associations of LTR7 loci acquisitions in primate genomes with timelines of species segregation processes during primate evolution (Fig. 1D).

**Fig. 2:**
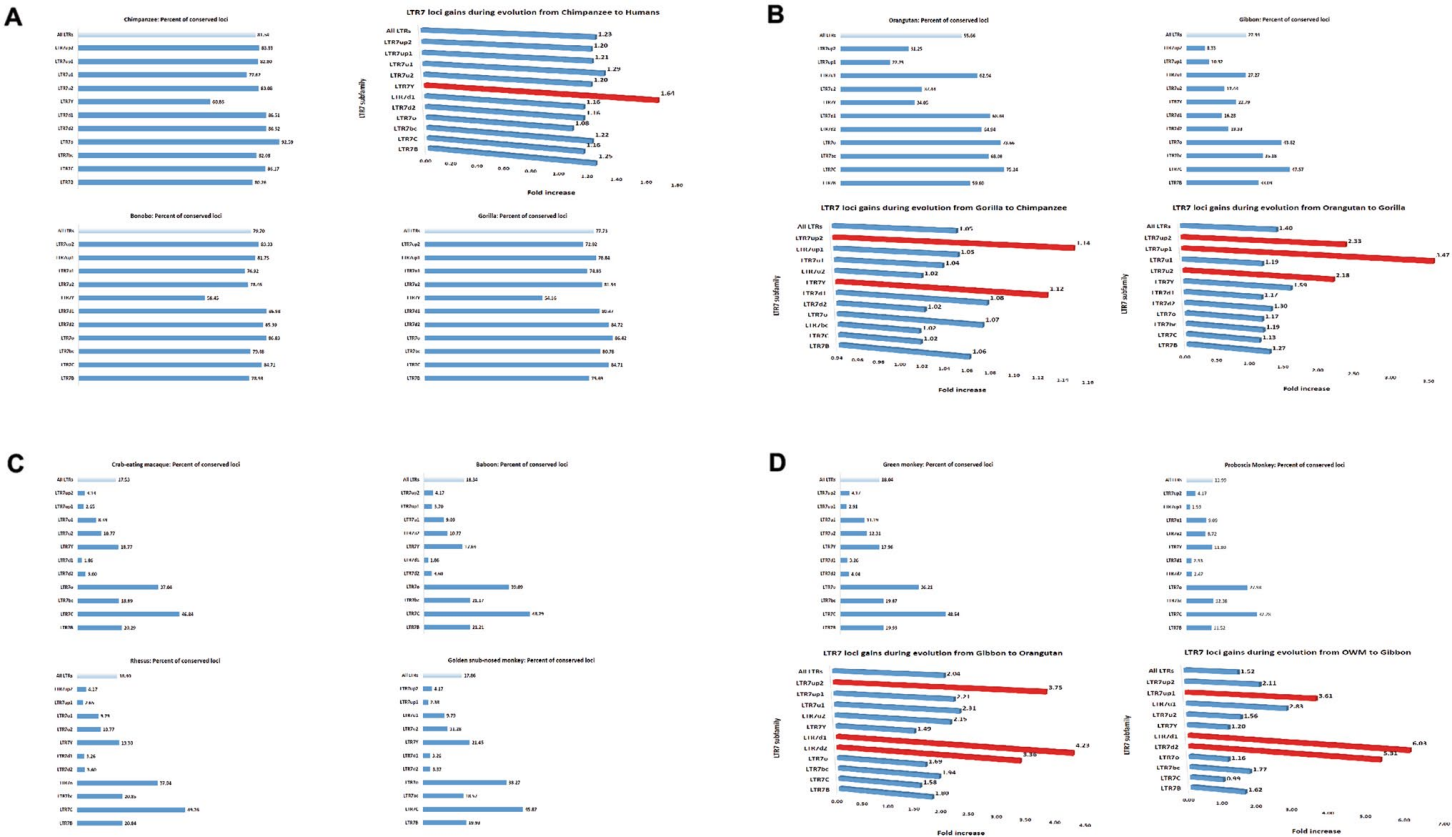
Granular evolutionary conservation analysis of eleven regulatory LTR7 subfamilies identifies subfamilies that are most rapidly expanding at different stages of species segregation during primate evolution. Numbers of highly conserved orthologous LTR7 loci of each subfamily were determined for each primate species and reported as the percentage of corresponding loci residing in genomes of Modern Humans. Relative gains of corresponding LTR7 subfamilies were calculated as ratio of highly conserved loci in designated species. Red colored bars denote the most rapidly expanding LTR7 subfamilies at indicated evolutionary stages

Recent investigations of fine molecular structures of LTR7 elements and their genetic and regulatory distinctions demonstrated that LTR7 sequences represent a complex polyphyletic group composed of at least eight monophyletic subfamilies (Carter et al. 2022). Next, sequence conservation analyses of each individual monophyletic subfamilies of LTR7 loci in genomes of sixteen NHP have been performed. It has been observed that highly conserved sequences of all eleven monophyletic LTR7 subfamilies are present in genomes of all Old World Monkeys’ species analyzed in this contribution as well as in genomes of Gibbon, Orangutan, Gorilla, Bonobo, and Chimpanzee (Fig. 2). These observations suggest that diversification of LTR7 loci into distinct genetic and regulatory subfamilies may have occurred early during primate evolution and subsequent cycles of LTR7 expansion appear to maintain this diversity.

**Fig. 3:**
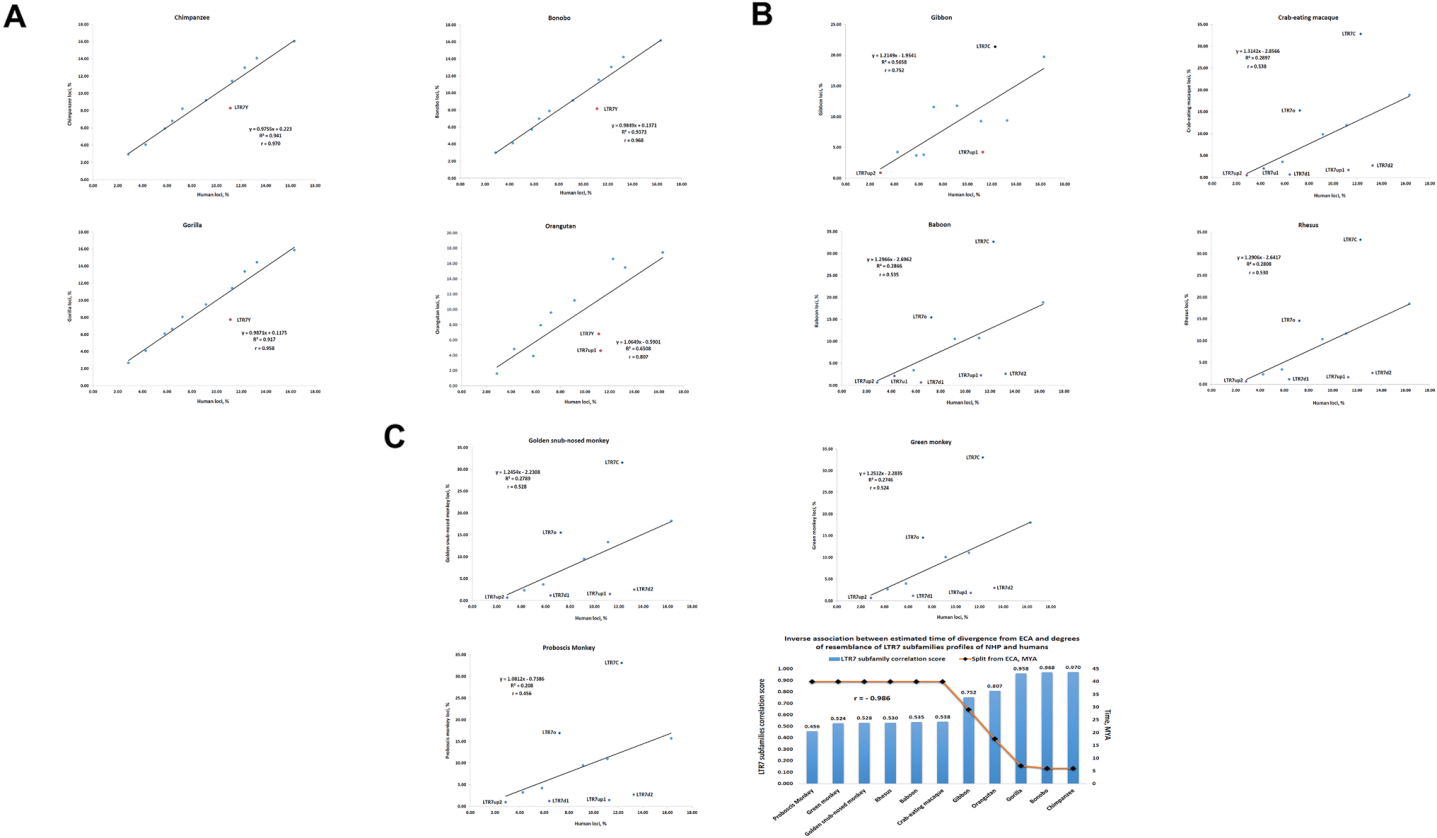
Correlation analyses of the granular evolutionary conservation patterns of regulatory LTR7 subfamilies. Granular evolutionary conservation patterns are presented as correlation plots of abundance profiles of LTR7 subfamilies in genomes of each non-human primate species vis-a-vis Modern Humans. Abundance profiles of LTR7 subfamilies were independently determined for each species and reported as percentage of loci of a given subfamily in a species’ genome. Note strikingly similar correlation coefficients for closely-related primate species which is gradually decreasing with increasing distance of species segregation. The inverse association is reported between the estimated times of divergence from ECA and degrees of resemblance of LTR7 subfamilies abundance profiles of NHP species and Modern Humans (C; bottom right panel)

Notably, despite the large difference in numbers of LTR7 loci present in genomes of different NHP species, the overall balance among eleven different LR7 subfamilies appears substantially similar (Fig. 3). A great degree of resemblance is particularly evident for evolutionary closely related species (Fig. 3 and data not shown), which is exemplified by high correlation coefficient values of LTR7 subfamilies’ abundance profiles estimated in pair-wise comparisons between Humans and Chimpanzee (*r* = 0.970), Bonobo (*r* = 0.968), and Gorilla (*r* = 0.958). Analyses of degrees of resemblance of LTR7 subfamilies abundance profiles among genomes of Great Apes revealed nearly identical arrangements of LTR7 subfamilies composition: Chimpanzee and Bonobo comparison yielded a pair-wise correlation coefficient of 0.999, while Chimpanzee and Gorilla comparison resulted in a pair-wise correlation coefficient of 0.998. Similarly, inter-species correlation coefficients for pair-wise comparisons between different Old World Monkeys species consistently exceeded values of 0.99. When the abundance profile of LTR7 subfamilies in genomes of Modern Humans was utilized as a reference, a gradual decline of correlation coefficient values of LTR7 subfamilies’ abundance profiles estimated in pair-wise comparisons between Humans and more distant NHP species has been observed (Fig. 3). A graphical summary of these findings reported in the Fig. 3C illustrates the inverse association pattern between estimated times of divergence from ECA and degrees of resemblance of LTR7 subfamilies abundance profiles in genomes of NHP and Modern Humans (Fig. 3C).

### Evolutionary conservation and divergence patterns of human-specific insertions of HERVH promoter LTR7 and HERVK promoter LTR5_Hs

**Fig. 4:**
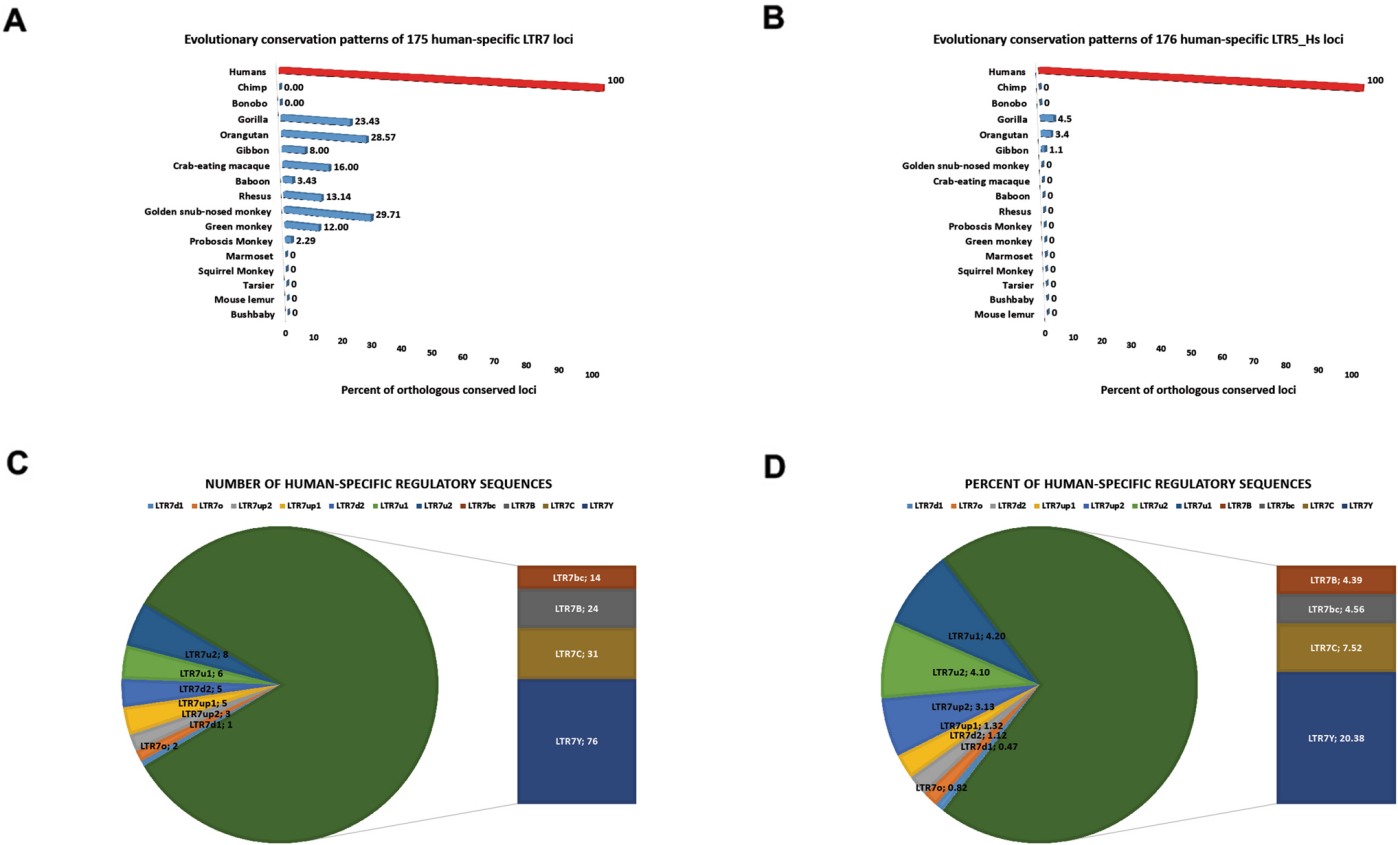
Evolutionary conservation patterns of human-specific LTR7 (**A**) and LTR5_Hs (**B**) loci in primates’ genomes identify highly conserved orthologous LTR sequences in genomes of NHP representing remnants of past retroviral expansion events during primate evolution. Granular analyses of numbers (**C**) and percentages (**D**) of human-specific LTR7 elements’ distribution among eleven monophyletic LTR7 subfamilies identify LTR7Y subfamily as a dominant source of human-specific insertions in genomes of Modern Humans. In **D,** percentages of human-specific loci for each LTR7 subfamily are reported calculated as fractions of all LTR7 sequences of corresponding subfamily

Results of LTR loci sequence conservation analyses indicate that hundreds of LTRs in genomes of our closest evolutionary relatives, Chimpanzee and Bonobo, have DNA sequences divergent by more than 5% from orthologous sequences in genomes of Modern Humans. It was of interest to determine how many of fixed non-polymorphic LTR loci identified in human genome could be defined as human-specific compared to both Chimpanzee and Bonobo genomes. Setting a selection threshold at < 10% of sequence identity, there are 175 LTR7 sequences and 176 LTR5_Hs sequences that could be classified as human-specific loci (Fig. 4). Notably, a majority of candidate human-specific LTR7 (149/175; 85%) and LTR5_Hs (139/176; 80%) loci could be classified as bona fide human-specific insertions because they did not intersect any chains in genomes of both Chimpanzee and Bonobo.

However, evolutionary conservation analyses revealed that nearly half (84/175; 48%) of human-specific LTR7 loci could be mapped as highly conserved sequences present in genomes of Old World Monkeys, Gibbon, Orangutan, and Gorilla (Fig. 4A), suggesting that these LTR7 loci were not retained in genomes of Chimpanzee and Bonobo but preserved in genomes of Modern Humans. In contrast, a much smaller fraction (8/176; 4.5%) of human-specific LTR5_Hs loci could be mapped as highly conserved sequences in genomes of Gibbon (2 loci), Orangutan (5 loci), and Gorilla (7 loci) (Fig. 4B).

Granular analyses of human-specific regulatory sequences among different LTR7 subfamilies revealed that the LTR7Y subfamily harbors nearly half (76/175; 43%) of all human-specific LTR7 loci (Fig. 4C). Overall, 20.4% of all LTRY sequences present in human genome could be classified as human-specific LTR7 loci (Fig. 4D), while other LTR7 subfamilies harbor much smaller fractions of sequences defined as human-specific loci (Fig. 4D).

### Inference of potential phenotypic impacts of human-specific insertions of HERVH promoter LTR7 and HERVK promoter LTR5_Hs

**Fig. 5:**
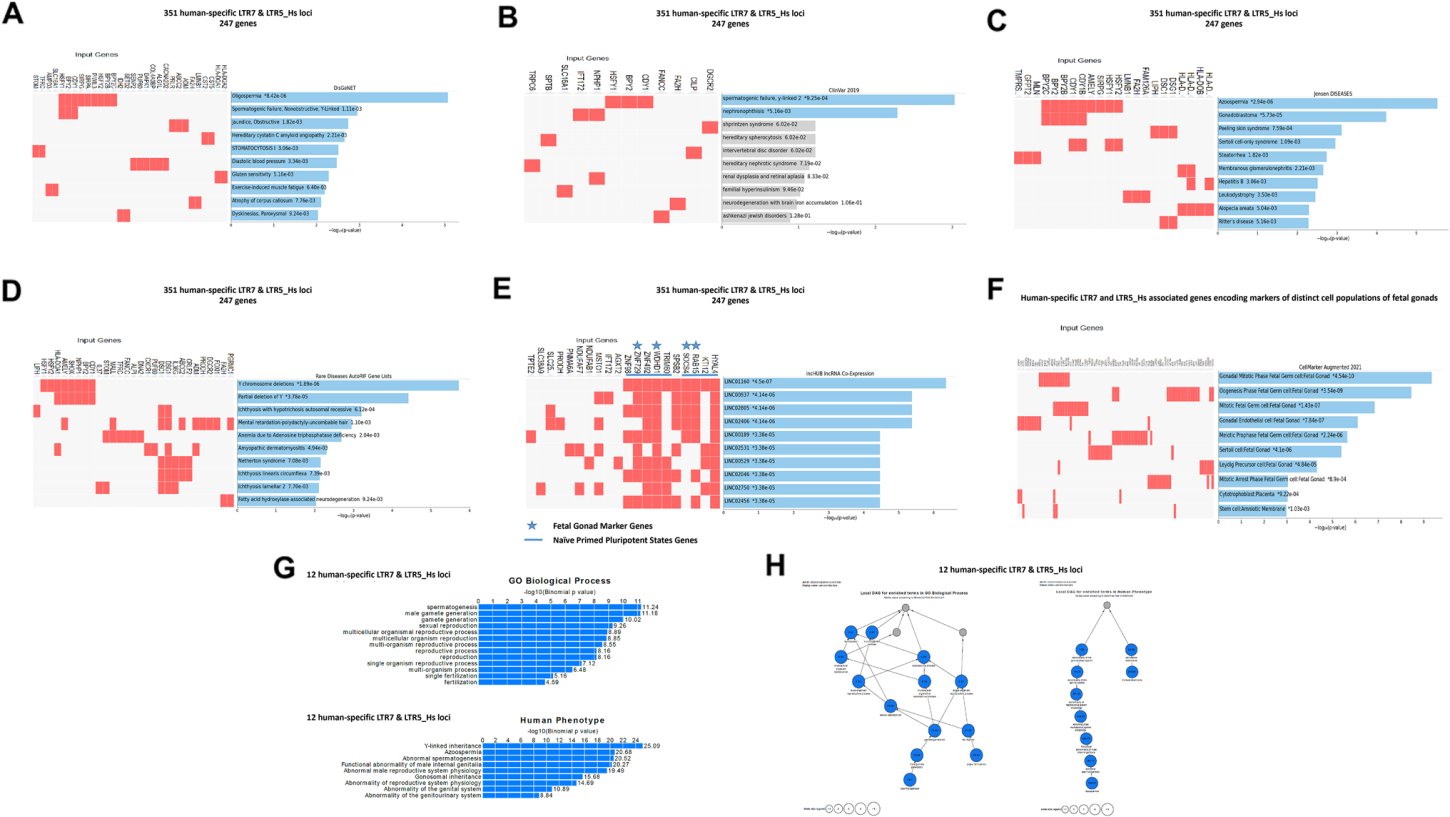
Inference of putative phenotypic impacts of human-specific LTR7 and LTR5_Hs loci based on GREAT algorithm-guided identification and functional annotations of proximity placement-linked genes. GSEA of 247 genes linked by GREAT with 351 human-specific LTR7 and LTR5_Hs using the Enrichr bioinformatics platform (**A**–**F**) and the GREAT algorithm-guided identification of 12 human-specific LTR7 elements (6 loci) and LTR5_Hs elements (6 loci) most significantly enriched in multiple GO Biological Process and Human Phenotype Ontology categories (**G**, **H**)

To infer potential biological functions of human-specific LTRs operating as distal regulatory loci, the GREAT algorithm was employed (Methods) to define the genome-wide connectivity maps of human-specific LTRs and their putative target genes. Concurrently with identification of putative regulatory target genes of human-specific LTRs, the GREAT algorithm performs stringent statistical enrichment analyses of functional annotations of identified genes, thus enabling the inference of potential biological significance of interrogated GRNs. Concurrently, a comprehensive panel of GSEA was executed employing the Enrichr bioinformatics platform (Methods) implemented on ~ 30 genomics and proteomics databases (Methods) by imputing candidate down-stream regulatory target genes of human-specific LTRs (Fig. 5; Supplementary Figures S1 and S2).

Consistent with documented biological roles of LTR7 and LTR5_Hs regulatory loci in establishment and maintenance of stemness and pluripotency phenotypes (Introduction), GSEA of 247 genes linked by GREAT with 351 human-specific LTR7 and LTR5_Hs revealed that a majority of significantly enriched records (70% of top 10 enriched records) represents genes associated with naïve and primed pluripotent states (GSEA of the database of human RNA seq GEO signatures; Supplementary Figure S1). Notable enrichment patterns of potential biological interest and translational significance were observed during the GSEA of several genomics databases of human common and rare diseases (Fig. 5). Top significantly enriched records identified by GSEA were Oligospermia (*p* = 8.42E-06; DisGeNET database); Spermatogenic Failure, Y-linked (*p* = 9.25E-04; ClinVar 2019 database), Azoospermia (*p* = 2.94E-06; Jensen Diseases database), Y chromosome deletions (*p* = 1.89E-06; Rare Diseases AutoRIF Gene Lists database) (Fig. 5), suggesting that regulation of human spermatogenesis might be one of biologically important functions of genes under putative regulatory control of human-specific LTR7 and LTR5_Hs loci. Consistent with this idea, the GREAT algorithm analysis of 351 human-specific LTR7 and LTR5_Hs loci identified seven human genes (*BPY2; CDY1; DAZ2; HSFY1; RPS4Y2; SRY; LMNB1*) associated by the Human Phenotype Ontology database analysis (Supplementary Table S1) with phenotypes of Y-linked inheritance (HP:0001450; *p* = 3.92E-07); Abnormal male reproductive system physiology (HP:0012874; *p* = 1.65E-05); Azoospermia (HP:0000027; *p* = 3.87E-05); Abnormal spermatogenesis (HP:0008669; *p* = 5.22E-05); Functional abnormality of male internal genitalia (HP:0000025; *p* = 8.37E-05). Follow-up analytical experiments focused on human-specific LTR loci and genes linked with the listed above human phenotypes confirmed these observations. The GREAT algorithm-defined connectivity map of regulatory loci and target genes identified 12 human-specific sequences of LTR7 (6 loci) and LTR5_Hs (6 loci) that manifested statistically significant enrichments in GO Biological Process (13 significantly enriched records) and Human Phenotype Ontology (9 significantly enriched records) categories (Fig. 5; Supplementary Figure S1).

GSEA of lncHUB lncRNA Co-Expression database defined 30 significantly enriched records of human long non-coding RNA molecules (lncRNAs) that are co-expressed in human tissues with a sub-sets of genes representing putative regulatory targets of human-specific LTRs (Fig. 5; Supplementary Figure S2). Detailed investigation of co-expressed loci demonstrated that 8 of 10 genes co-expressed with top-scoring LNC01160 lncRNA (*p* = 4.5E-07; Fig. 5) represent genes differential expression of which distinguishes Naïve and Primed pluripotent states, while four genes constitute genetic markers of fetal gonads (Fig. 5; Supplementary Figure S2). Next, follow-up analyses were carried out employing the CellMarker Augmented 2021 database of single-cell genomics-guided genetic markers of human and mouse cells comprising gene sets from the CellMarker database augmented with co-expression RNA seq data from ARCHS4 (Enrichr). These analyses identified 88 genes representing putative regulatory targets of human-specific LTR7 and LTR5_Hs loci and comprising genetic markers of 12 distinct cell populations of fetal gonads (Fig. 5; Supplementary Figure S2). Collectively, these observations suggest that genes implicated in development of human fetal gonads may represent regulatory targets of human-specific LTR7 and LTR5_Hs loci. Consistent with this hypothesis, expression of nearly three quarters of identified herein fetal gonad marker genes (64 of 88 genes; 73%) is significantly altered in human cells subjected to genetic and/or epigenetic targeting of LTR7/HERVH or LTR5_Hs loci (see below).

### GSEA of retroviral LTRs-linked genes revealed dominant enrichment patterns of physiological and pathological phenotypic traits affected by mammalian offspring survival (OS) genes associated with LTR7 and LTR5_Hs regulatory loci

DNA sequences derived from LTR7/HERVH and LTR5_Hs/HERVK retroviral insertions have been identified as one of significant sources of the evolutionary origin of human-specific regulatory sequences (HSRS), including transcription factor-binding sites (TFBS) for stemness state master regulators NANOG, OCT4, and SOX2 (Glinsky 2015, 2016a, 2016b, 2017, 2018, 2019, 2020a, 2020b, 2020c, 2021). Since mammalian offspring survival (OS) genes have been implicated as one of the putative genomic regulatory targets of HSRS (Glinsky, 2020a, b, c), it was of interest to determine whether mammalian OS genes are enriched among candidate regulatory targets of LTR7 and LTR5_Hs loci. Among 18,777 human genes comprising the GREAT database gene set (hg38 release of the human reference database), there are 2413 mammalian OS genes defined as genes mutations of which have been associated by the MGI database search with premature death, embryonic lethality, as well as pre-, peri-, neo-, and post-natal lethality phenotypes of both complete and incomplete penetrance. Of these, a total of 562 mammalian OS genes has been identified as putative regulatory targets of LTR7 loci (Table 1), which represents a significant enrichment compared to expected by chance number of genes (*p* = 1.131E-10; 2-tail Fisher’s exact test). In contrast, the number of mammalian OS genes identified as putative regulatory targets of LTR5_Hs loci (126 genes) did not exceed the enrichment significance threshold level (Table 1). Detailed analyses of the enrichment levels’ distribution of mammalian OS genes among different LTR7 subfamilies demonstrate that proportions of OS genes appear enriched among putative LTR7 regulatory targets of various LTR7 subfamilies (Table 2). These observations suggest that mammalian OS genes seem to remain one of favorite regulatory targets throughout ~ 30 MYA of the divergent evolution of LTR7 loci.

**Table 1:** Enrichment patterns of mammalian offspring survival (OS) genes linked with LTR7 and LTR5_Hs loci

Classification category	Number of genes
LTR7-linked genes	2957
LTR7 OS networks genes	562
Observed, %	19.01
Expected, %	12.85
Enrichment	1.48
P value*	1.131E-10
LTR5_Hs-linked genes	935
LTR5_Hs OS networks genes	126
Observed, %	13.48
Expected, %	12.85
Enrichment	1.05
P value*	0.732

Enrichment *p* values were estimated employing 2-tail Fisher’s exact test

**Table 2:** Association patterns of distinct subfamilies of regulatory LTR7 loci with offspring survival (OS) genes

LTR7 subtype	Number of loci	Number of LTR7-linked genes	Number of LTR7-linked OS genes	Percent, OS genes	Enrichment
LTR7B	547	864	145	16.78	1.31
LTR7C	412	622	115	18.49	1.44
LTR7bc	307	486	91	18.72	1.46
LTR7o	243	380	76	20.00	1.56
LTR7d1	215	361	70	19.39	1.51
LTR7d2	445	685	144	21.02	1.64
LTR7u1	143	237	48	20.25	1.58
LTR7u2	195	321	76	23.68	1.84
LTR7up1	378	575	119	20.70	1.61
LTR7up2	96	152	34	22.37	1.74
LTR7Y	373	454	79	17.40	1.35
All LTR7 loci	3354	2957	562	19.01	1.48

**Fig. 6:**
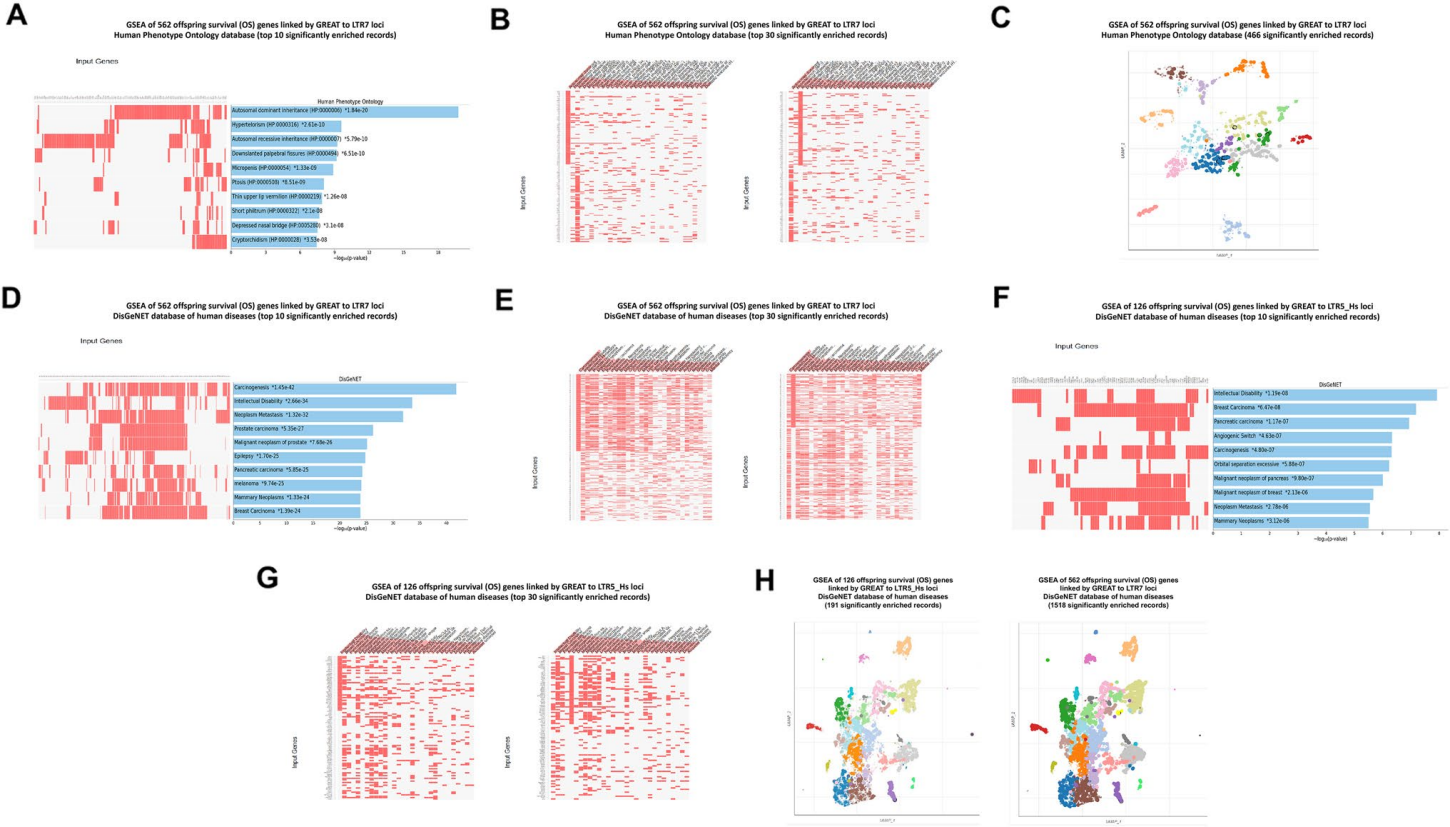
Analysis of phenotypic impacts of LTR7—and LTR5_Hs—linked mammalian offspring survival (OS) genes revealed by GSEA of the Human Phenotype Ontology database (**A**–**C**) and the DisGeNET database of human diseases (**D**–**H**). In figures (**B**; **G**; and **C**) input genes are sorted by different phenotypic traits in the left and right panels to demonstrate the overlapping patterns of genes associated with different phenotypic traits. The scatterplots in (**C**) and (**H**) are organized so that similar gene sets are clustered together. Larger, darker, black-outlined points represent significantly enriched terms. Clusters are computed using the Leiden algorithm. Points are plotted on the first two UMAP dimensions. In web-based software Enrichr settings, hovering over points will display the associated gene set name and the *p* value. Reader may have to zoom in using the toolbar next to the plot to see details in densely populated clusters. Plots can also be downloaded as a svg using the save function on the toolbar

It was of interest to determine whether mammalian OS genes may have the broader impacts on pathophysiology of Modern Humans extending beyond offspring survival phenotypes. To this end, independent GSEA were carried out on all genes defined as putative regulatory targets of LTR7 and LTR5_Hs loci and sub-sets of regulatory targets comprising mammalian OS genes and non-OS genes (Table 3). These analyses revealed clearly discernable dominant enrichment patterns of phenotypic traits affected by mammalian OS genes linked with LTR7 and LTR5_Hs regulatory loci across the large panel of genomics and proteomics databases reflecting a broad spectrum of human pathophysiology (Table 3). Enrichment patterns’ differences were particularly notable for GSEA of databases of human common and rare diseases as well as Human Phenotype Ontology database, suggesting that mammalian OS genes may have significant impacts on development of phenotypic traits and pathological conditions of Modern Humans. Significantly, clearly-defined heritability features seem apparent for a majority of LTR7-linked mammalian OS genes associated with 466 phenotypic traits reported in the Human Phenotype Ontology database because they are represented by either Autosomal dominant inheritance genes (HP: 0,000,006; *p* = 1.84E-20) or Autosomal recessive inheritance (HP: 0,000,007; *p* = 5.79E-10) genes (Fig. 6). Three top-ranked phenotypic traits captured by GSEA of 562 LTR7-linked mammalian OS genes employing the DisGeNET database of human diseases (Carcinogenesis; *p* = 1.45E-42; Intellectual disability; *p* = 2.66E-34; Neoplasm metastasis; *p* = 1.32E-32) appear associated with overlapping networks of genes (Fig. 6), perhaps, reflecting yet poorly understood common mechanistic features for malignant neoplasms and brain disorders. Consistent with this hypothesis, a qualitatively similar genotype–phenotype association patterns were documented by GSEA of 126 LTR5_Hs-linked mammalian OS genes (Fig. 6).

**Table 3:** Dominant enrichment patterns of phenotypic traits affected by offspring survival (OS) genes linked with LTR7 and LTR5_Hs regulatory loci

Classification category	LTR7	LTR7 OS genes	LTR7 non-OS genes	LTR5_Hs	LTR5_Hs OS genes	LTR5_Hs non-OS genes
Number of associated genes	2957	562	2395	935	126	809
Significantly enriched records of phenotypic traits identified by the GSEA of genomic databases						
DisGeNET database	72	1518	0	1	191	1
PanglaoDB augmented 2021	70	67	33	3	4	0
CellMarker augmented 2021	203	181	83	3	0	2
Azimuth cell types 2021	89	17	47	0	0	0
ARCHS4 human tissues	36	37	24	2	11	0
Human gene atlas	2	10	0	0	1	0
Allen brain atlas database of up-regulated genes	420	506	43	0	0	0
Allen brain atlas database of down-regulated genes	101	230	5	0	1	0
GTEx tissues’ expression database of up-regulated genes	334	583	129	0	48	0
GTEx tissues’ expression database of down-regulated genes	98	329	56	0	80	0
Reactome 2016	3	50	0	2	77	1
WikiPathway 2021 human	1	68	0	0	20	0
KEGG 2021 human	2	38	1	1	15	1
BioPlanet 2019	6	111	1	3	4	1
Jensen diseases database	40	75	15	4	6	3
MGI mammalian phenotype level 4 2021	25	1253	0	0	207	0
Human phenotype ontology	3	466	0	0	26	0
GTEx aging signatures 2021	14	0	11	0	0	0
Disease perturbations from GEO up-regulated genes	63	160	14	0	0	0
Disease perturbations from GEO down-regulated genes	88	153	23	0	0	0
Significantly enriched records of phenotypic traits identified by the GSEA of human rare diseases databases						
Rare diseases GeneRIF gene lists	1	497	0	0	5	0
Rare diseases AutoRIF gene lists	15	738	0	0	2	0
Rare diseases AutoRIF ARCHS4 predictions	57	161	0	0	0	0
Rare diseases GeneRIF ARCHS4 predictions	47	137	0	0	11	0
Significantly enriched records of phenotypic traits identified by the GSEA of targeted TF perturbations, TF PPI, and PPI Hub Proteins genomic databases						
TF perturbations followed by expression	368	704	76	4	26	1
Transcription factor PPIs	0	86	0	0	0	0
PPI hub proteins	0	105	0	0	9	0

Gene Set Enrichment Analyses (GSEA) were carried out for each gene set and numbers of significantly enriched phenotypic traits defined at the adjusted *p* values < 0.05 were recorded. Complete lists of phenotypic traits, associated genes, and corresponding statistical metrics for analyzed genomic databases are reported in the Supplement

*TF* transcription factors, *PPI* protein–protein interactions, *GEO* gene ontology omnibus

Exceptions from quantitatively dominant enrichment patterns of phenotypic associations of mammalian OS genes were noted for a database reporting genetic markers of human tissues (ARCHS4 Human Tissues database; Table 3) and several databases of cell-type specific markers (PanglaoDB Augmented 2021; CellMarker Augmented 2021; and Azimuth Cell Types 2021 databases; Table 3). These findings demonstrate consistent tissue- and cell-type specific expression profiles of both mammalian OS genes and non-OS genes comprising putative regulatory targets of LTR7 loci, perhaps, reflecting their contributions to functions of a broad spectrum of differentiated cells in human body (Table 3). In contrast, GSEA of LTR7-linked genes focused on the GTEx Aging Signatures 2021 database revealed that significantly associated traits of numerous human aging tissues (nerve; small intestine; uterus; beast; brain; lung; blood vessels) appear linked with non-OS genes (Table 3; Supplementary Table S2).

A total of 704 significantly enriched records of genetic targeting of transcription factor (TF)—coding genes affected expression of large numbers of mammalian OS genes comprising putative regulatory targets of LTR7 loci, which significantly exceeded numbers of targeted genes expected by chance (Table 3; Supplementary Table S2). These observations indicate that reported herein LTR7-linked genes may function in human cells as down-stream targets of genomic regulatory networks governed by hundreds of regulatory interactions of host TFs and down-stream target genes. Intriguingly, protein products of many mammalian OS genes comprising putative regulatory targets of LTR7 loci were identified as partners of protein–protein interactions (PPI) of at least 86 TFs and 105 PPI Hub proteins, which are proteins that interact with protein products encoded by at least 50 other genes. These findings support the hypothesis that engagements in PPI of the multi-molecular complexes operating in human cells may represent an important mechanistic vector of biological activities of mammalian OS genes comprising putative regulatory targets of LTR7 loci.

### Identification and characterization of retroviral LTR7 and LTR5_Hs loci associated with genes regulating synaptic transmission and protein–protein interactions at synapses

GSEA of genomic databases focused on gene expression signatures of human tissues and cell types revealed a clear prevalence of enrichment records related to brain and CNS functions among significantly enriched phenotypic traits affected by genes comprising putative regulatory targets of LTR7 loci (Supplementary Table S2). For example, records of cell types and tissues related to brain and CNS functions constitute 55% and 75% of top 20 significantly enriched records identified by GSEA of the single-cell sequencing PanglaoDB Augmented 2021 and ARCHS4 Human Tissues databases, respectively. Strikingly, all top 20 significantly enriched records (Supplementary Table S2) identified by GSEA of the GTEx human tissues' expression database of up-regulated genes and single-cell genomics-guided Azimuth Cell Types 2021 database reference either brain samples (GTEx database) or different highly specialized types of GABAergic and Glutamatergic neurons (Azimuth database). Overall, 82 of 89 (92%) of all significantly enriched records identified by GSEA of the Azimuth Cell Types 2021 database represent different highly specialized types of neurons (Supplementary Table S2). Significantly enriched records of cell types identified by GSEA of the single-cell sequencing PanglaoDB Augmented 2021 database represent cells of distinct neurodevelopmental stages and morphologically diverse cell types residing and functioning in human brain, which include Neural Stem/Precursor cells, Radial Glia cells, Bergman Glia cells, Pyramidal cells, Tanycytes, Immature neurons, Interneurons, Trigeminal neurons, GABAergic neurons, and Glutamatergic neurons (Supplementary Table S2). Collectively, these observations indicate that one of the important biological functions of genes comprising putative regulatory targets of LTR7 loci is contribution to development and functions of the human brain. Consistent with this hypothesis, GSEA of the Allen Brain Atlas database identified 521 significantly enriched records of different human brain regions harboring expression signatures of both up-regulated (420 brain regions) and down-regulated (101 brain regions) genes comprising putative LTR7 regulatory targets (Supplementary Table S2).

Based on these findings, it was of interest to determine whether genes that play the essential biological role in brain functions are enriched among putative LTR7-taget genes. To test this hypothesis, records of 355 genes defined by the Reactome database as genes whose products regulate the synaptic transmission and are engaged in protein–protein interactions at synapses (collectively designated here synaptic transmission networks’ genes) were retrieved and analyzed. It has been determined that there are 87 synaptic transmission networks’ genes comprising putative regulatory targets of LTR7 loci (Table 4), which represents the significant enrichment compared to the expected by chance value (*p* = 0.01; 2-tail Fisher’s exact test). In contrast, there were only 19 synaptic networks’ genes among genes comprising putative regulatory targets of LTR5_Hs loci (Table 4), which corresponds to the expected by chance value.

**Table 4:** Enrichment patterns of synaptic networks genes linked with LTR7 and LTR5_Hs loci

Classification category	Number of genes
LTR7-linked genes	2957
LTR7 Synaptic networks genes	87
Observed, %	2.94
Expected, %	1.89
Enrichment	1.56
*P* value*	0.0109
LTR5_Hs-linked genes	935
LTR5_Hs Synaptic networks genes	19
Observed, %	2.03
Expected, %	1.89
Enrichment	1.07
*P* value*	1

Enrichment *p* values were estimated employing 2-tail Fisher’s exact test

**Fig. 7:**
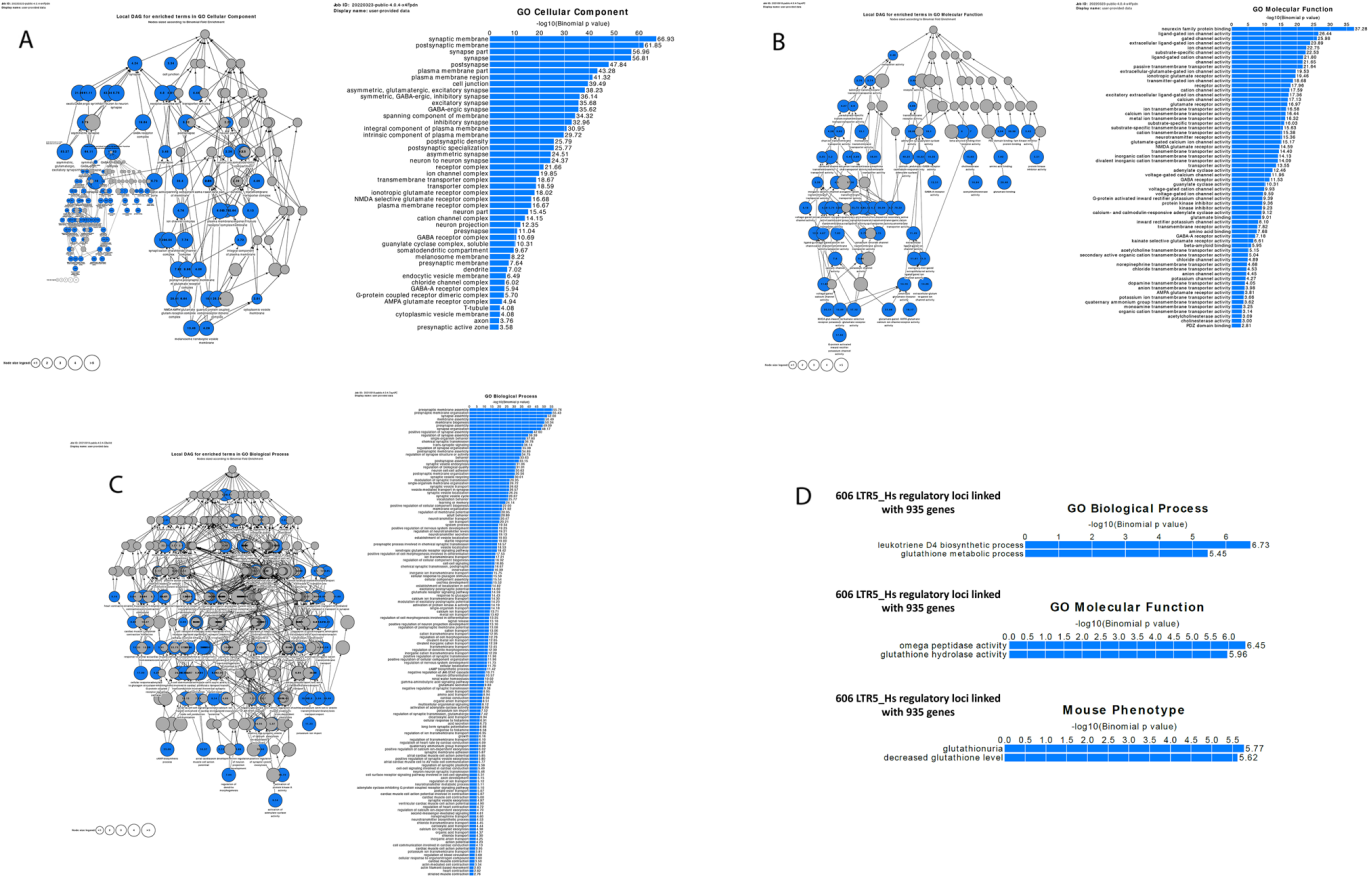
Identification and characterization LTR7 regulatory loci linked with genes whose products affect transmission across synapses and protein–protein interactions at synapses. Potential phenotypic impacts of 235 human regulatory LTR7 loci linked by the GREAT algorithm with 170 down-stream target genes revealed by interrogation of GO Cellular Component database (panel A; 46 significant terms), GO Molecular Function database (panel B; 64 significant terms), and GO Biological Process database (panel C; 146 significant terms). In contrast, analyses of 606 LTR5_Hs regulatory loci linked by GREAT with 935 gene identify 2 significantly enriched terms in GO Biological Process, GO Molecular Function, and Mouse Phenotype databases. Analyses were carried out using the GREAT algorithm designed to predict functions of cis-regulatory regions. Genomic coordinates of interrogated LTRs were based on the hg38 release of the human reference genome database

Next, the assessments were made of regulatory loci/target genes connectivity patterns with respect to synaptic transmission networks’ genes for individual LTR7 subfamilies. To this end, 220 LTR7 loci linked to 87 synaptic transmission networks’ genes by the GREAT algorithm (Table 5; Supplementary Table S3) were retrieved, all genes comprising their putative regulatory targets were identified, and numbers (fractions) of associated synaptic transmission networks’ genes were determined for each LTR7 subfamily (Table 5). It has been determined that all LTR7 subfamilies appear to manifest putative regulatory links to synaptic transmission networks’ genes (Table 5), suggesting that observed associations between LTR7 loci and synaptic transmission networks’ genes remain relatively constant during primate evolution. Notably, when a hypothetical genomic regulatory network connecting LTR7 loci and synaptic transmission networks’ genes was interrogated using the GREAT algorithm, Gene Ontology analyses identified numerous highly significantly enriched phenotypic traits affected by synaptic transmission networks’ genes linked with LTR7 loci (Fig. 7). For example, analysis of GO Cellular Component database revealed 46 significantly enriched terms, while GO Molecular Function database identified 64 significantly enriched terms and GO Biological Process database defined 146 significantly enriched records (Fig. 7; Supplementary Table S3). In contrast, the GREAT algorithm identified a single significantly enriched record employing Human Phenotype ontology database, namely Autism (Binominal FDR q value = 1.313E-15).

**Table 5:** LTR7 regulatory loci linked with genes mediating transmission across synapses and protein–protein interactions at synapses

LTR7 subfamily	Number of loci*	Number of linked genes	Number of synaptic transmission network genes**	Percent of synaptic transmission network genes
LTR7B	28	37	22	59.5
LTR7C	24	38	19	50.0
LTR7bc	29	46	26	56.5
LTR7o	23	34	18	52.9
LTR7d1	15	26	15	57.7
LTR7d2	39	60	31	51.7
LTR7u1	10	14	9	64.3
LTR7u2	13	21	12	57.1
LTR7up1	23	36	18	50.0
LTR7up2	5	8	4	50.0
LTR7Y	11	18	10	55.6
All LTR7 loci	220	170	87	51.2

*Number of individual non-redundant LTR7 regulatory loci; in total, there are 235 regulatory loci linked with 170 human genes by the GREAT algorithm, including synaptic transmission networks’ genes

**Reported results are based on the Reactome database analysis of 355 synaptic transmission network genes

**Fig. 8:**
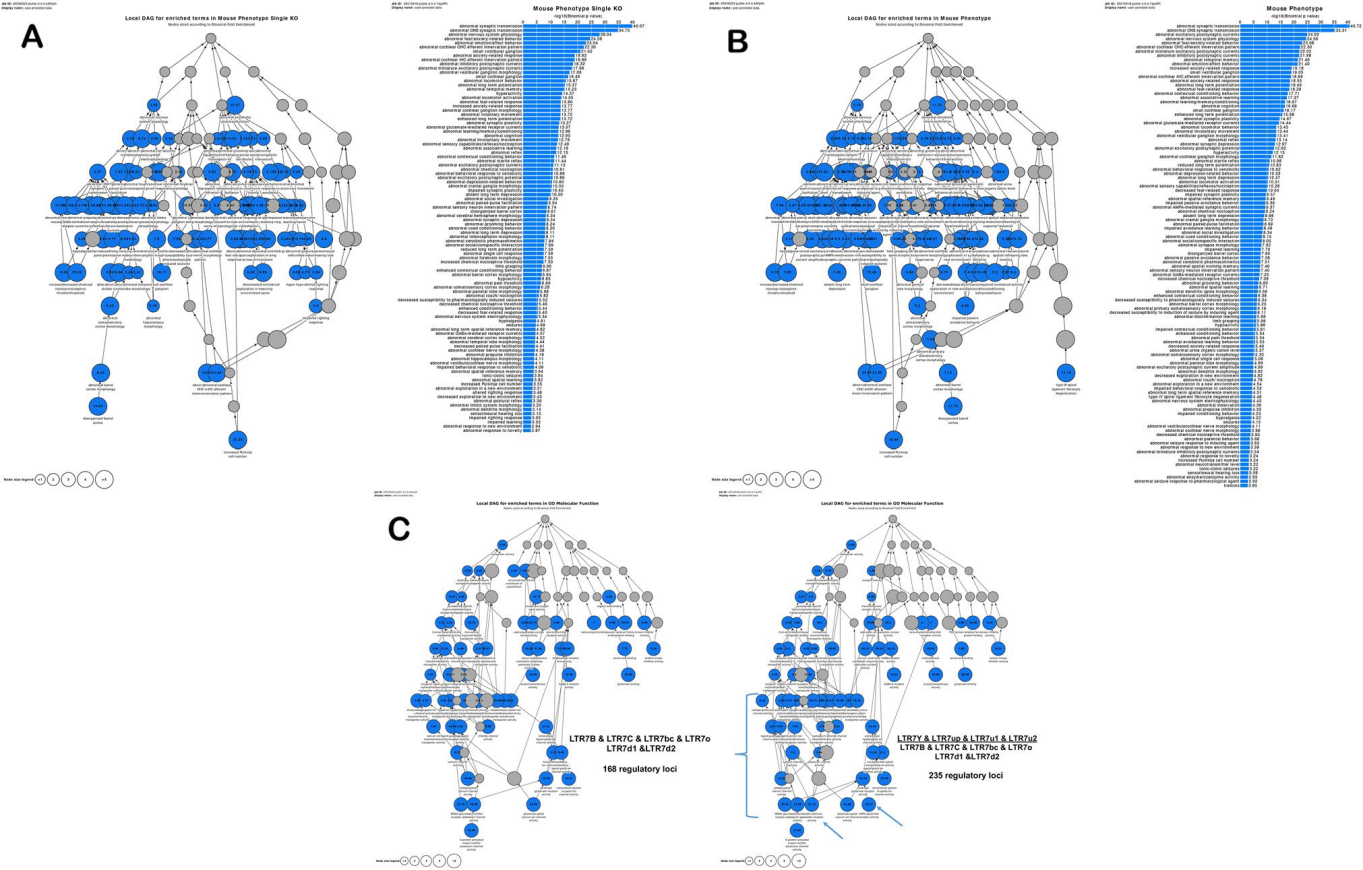
Potential phenotypic impacts of 235 regulatory LTR7 loci revealed by interrogation of Mouse Phenotype Single KO database (panel A; 97 significant terms) and Mouse Phenotype database (panel B; 110 significant terms). Differential GO Molecular Function analyses of genetically and evolutionary distinct subfamilies of human regulatory LTR loci (panel C) comprising 168 and 235 LTR7 loci designed to highlight LTR7-linked phenotypic traits enriched in humans after separation of chimpanzee and human lineages (depicted by arrows are the kainate selective glutamate receptor activity and AMPA glutamate receptor activity). Analyses were carried out using the GREAT algorithm designed to predict functions of cis-regulatory regions. Genomics coordinates of LTR7 loci were based on the hg38 release of the human reference genome database

Numerous significantly enriched records were identified by GSEA of Mouse Phenotype (110 significant terms) and Mouse Phenotype Single KO (97 significant terms) databases (Fig. 8). These findings together with reported herein regulatory connectivity maps of HERV’s LTRs and their putative target genes provide readily available well-characterized mouse models for experimental interrogations of postulated causal regulatory effects of HERVs LTRs on specific target genes and respective phenotypes. For example, visualization of significantly enriched records identified by GO Molecular Function analysis at different stages of primate evolution depicts activities of the kainate selective glutamate receptor and the AMPA glutamate receptor as potentially important biological functions during the evolutionary transition period from Great Apes to Humans (Fig. 8). Analyses of evolutionary dynamics of connectivity patterns between candidate regulatory LTRs and down-stream target genes among specific functional and/or morphological categories suggest that dominant LTR expansion patterns during evolution of Great Ape consist of linking newly emerged regulatory LTR loci to either new down-stream target gene(s) or genes already integrated into LTR regulatory networks, which are contributing to and/or engaged within a specific phenotypic trait that already being targeted for evolutionary innovations at the earlier stages of primate evolution.

### Identification and characterization of high-confidence genetic regulatory targets of LTR7 and LTR5_Hs loci

Inferences of potential phenotypic impacts of LT7 and LTR5_Hs loci on physiological traits and pathological conditions of Modern Humans were based on GSEA-guided assessments of documented biological functions and morphological features of genes comprising putative regulatory of LTR loci identified by the GREAT algorithm. To extend this line of inquiry further, it was of interest to determine what fraction of candidate LTR regulatory target genes constitutes high-confidence transcriptional targets of LTRs in human cells defined as genes expression of which is altered following genetic targeting of LTRs. It has been determined that expression of a majority of genes (1570 of 2957; 53%) comprising candidate regulatory targets of LTR7 loci is significantly affected in hESC subjected to either targeted genetic interference with or epigenetic silencing of LTR7/HERVH loci (Table 6). Similarly, expression of a majority of genes (486 of 935 genes; 52%) identified as putative regulatory targets of LTR5_Hs loci is significantly altered in human teratocarcinoma cells following targeted epigenetic silencing or activation of LTR5_Hs loci (Table 7). Overall, expression of 1944 of 3515 genes (55%) comprising LTRs’ candidate regulatory targets was significantly affected following genetic or epigenetic manipulations of LTR7 and/or LTR5_Hs loci (Table 8). Based on these observations, it has been concluded that expression of a majority of genes identified herein as putative regulatory targets of LTR7 and/or LTR5_Hs loci appears altered in human cells following targeted genetic and/or epigenetic manipulations of LTR sequences, which is consistent with definition of these sets of genes as high-confidence down-stream regulatory targets of LTR7 and LTR5_Hs loci. Interestingly, genes defined as high-confidence down-stream regulatory targets of both LTR7 and LTR5_Hs loci represent 66.6% of all candidate regulatory targets of both LTR7 and LTR5_Hs loci (Table 8), which is significantly higher than corresponding metrics recorded for genes linked with either LTR7 loci (*p* = 1.53E-08; 2-tail Fisher’s exact test), LTR5_Hs loci (*p* = 1.70E-13; 2-tail Fisher’s exact test), or genes associated with at least two LTR7 loci (*p* = 9.958E-05; 2-tail Fisher’s exact test).

**Table 6:** A catalog of human genes identified as regulatory targets of LTR5_Hs elements

Classification category	Number of records
LTR5_Hs loci (hg38)	606
GREAT-linked genes	935
CRISPRi target genes*	4251
CRISPRi/GREAT target genes	447
KRABa target genes*	621
KRABa/GREAT target genes	191
CRISPRi/KRABa/GREAT target genes	**486**

*Number of LTR5_Hs target genes in the GREAT hg38 set of 18,777 human genes

**Table 7:** A catalog of human genes identified as regulatory targets of LTR7 elements

Classification category	Number of records
LTR7 loci (hg38)	3354
GREAT-linked genes	2957
CRISPRi LTR7Y/B target genes*	6735
CRISPRi/GREAT target genes	1012
HERVH target genes*	6110
HERVH/GREAT target genes	1017
CRISPRi/HERVH/GREAT target genes	**1570**

*Number of LTR7 target genes in the GREAT hg38 set of 18,777 human genes

**Table 8:** A significant majority of LTR-linked mammalian OS genes and fetal gonad’s cells marker genes are high-fidelity LTR-regulated genes

Classification category	Number of genes	LTR-regulated	Percent	*P* value*
LTR7 target genes	2957	1570	53.09	
LTR5_Hs target genes	935	486	51.98	
LTR7 and/or LTR5_Hs target genes	3515	1944	55.31	
LTR7 target genes (at least 2 LTR7 loci per gene)	1202	664	55.24	9.958E-05
LTR7 and LTR5_Hs target genes	377	251	**66.58**	9.958E-05
LTR7 only target genes	2580	1319	51.12	1.53E-08
LTR5_Hs only target genes	558	235	42.11	1.70E-13
LTR7 mammalian OS target genes	562	392	**69.75**	5.96E-19
LTR7 mammalian non-OS target genes	2395	1178	49.19	5.96E-19
LTR5_Hs mammalian OS target genes	126	92	**73.02**	2.85E-07
LTR5_Hs mammalian non-OS target genes	809	394	48.70	2.85E-07
LTR7 and/or LTR5_Hs mammalian OS target genes	630	432	**68.57**	8.34E-14
LTR7 and/or LTR5_Hs mammalian non-OS target genes	2885	1512	52.41	8.34E-14
LTR7 and LTR5_Hs mammalian OS target genes	58	50	**86.21**	0.0004271
LTR7 and LTR5_Hs mammalian non-OS target genes	319	201	63.01	0.0004271
Human-specific LTR7 and/or LTR5_Hs target genes	391	215	54.99	
Fetal gonad’s cells markers hsLTR7 and/or hsLTR5_Hs target genes	88	64	**72.73**	0.0001478
Human-specific LTR7 and/or LTR5_Hs target genes (excluding fetal gonad’s cell marker genes)	303	151	49.83	0.0001478

*P values were estimated using the 2-tail Fisher’s exact test

Fractions of genes representing high-confidence down-stream regulatory targets of LTR7 and LTR5_Hs loci appear significantly higher among mammalian OS genes compared to non-OS genes (Table 8), reaching 86.2% (*p* = 0.0004; 2-tail Fisher’s exact test) for mammalian OS genes defined as targets of both LTR7 and LTR5_Hs loci (Table 8). Similarly, 64 of 88 (72.7%) genes encoding Fetal Gonad’s cells markers associated with human-specific LTR7 and/or LTR5_Hs loci could be defined as their high-confidence down-stream regulatory targets (Table 8). Therefore, these functional categories and corresponding genes linked by present analyses to LTR7 and LTR5_Hs genomic regulatory networks should be considered as high-priority aims for experimental validation of bona fide transcriptional regulatory targets of LTR7 and LTR5_Hs loci.

**Fig. 9:**
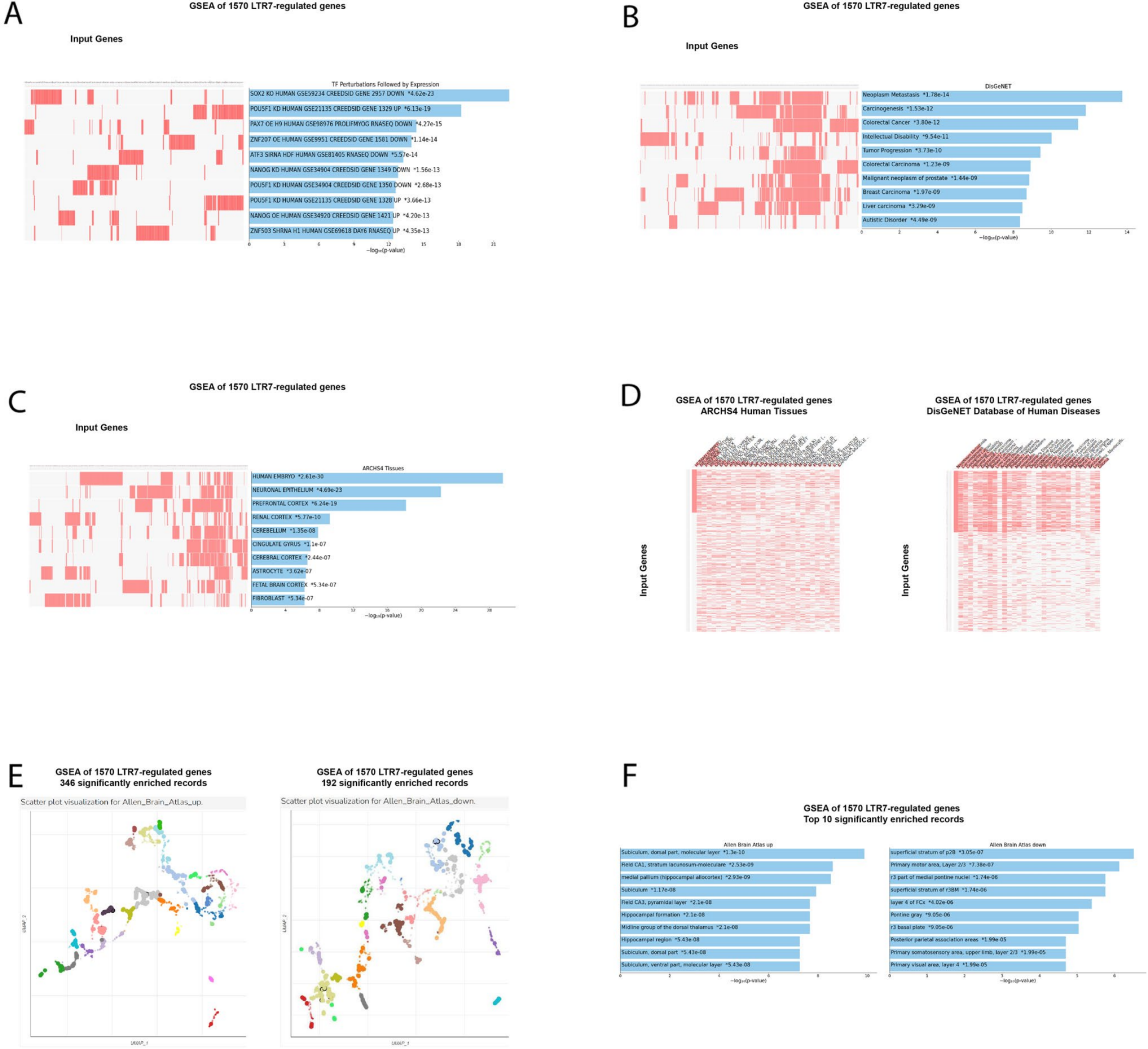
Potential phenotypic impacts of LTR7 elements revealed by GSEA of 1570 high-fidelity down-steam target genes employing the Transcription Factors (TF) perturbations followed by expression database (**A**), the DisGeNET database of human diseases (**B**; **D**), the ARCHS4 Human Tissues database (**C**, **D**), the Allan Brain Atlas databases of up-regulated genes (**E**, **F**; left panels) and down-regulated genes (**E**, **F**; right panels). In **D,** top 30 significantly enriched records of gene sets were sorted by genes comprising the expression signature of the Human Embryo (left panel) and genes comprising the expression signature of the Neoplasm Metastasis (right panel). In panels **A**, **B**, **C**, **F** results illustrating the top 10 significantly enriched records are reported. All reported significantly enriched records were identified at the significance threshold of adjusted *p* value < 0.05 by the GSEA of 1570 LTR7-regulated genes employing the corresponding genomic databases (Methods)

**Fig. 10:**
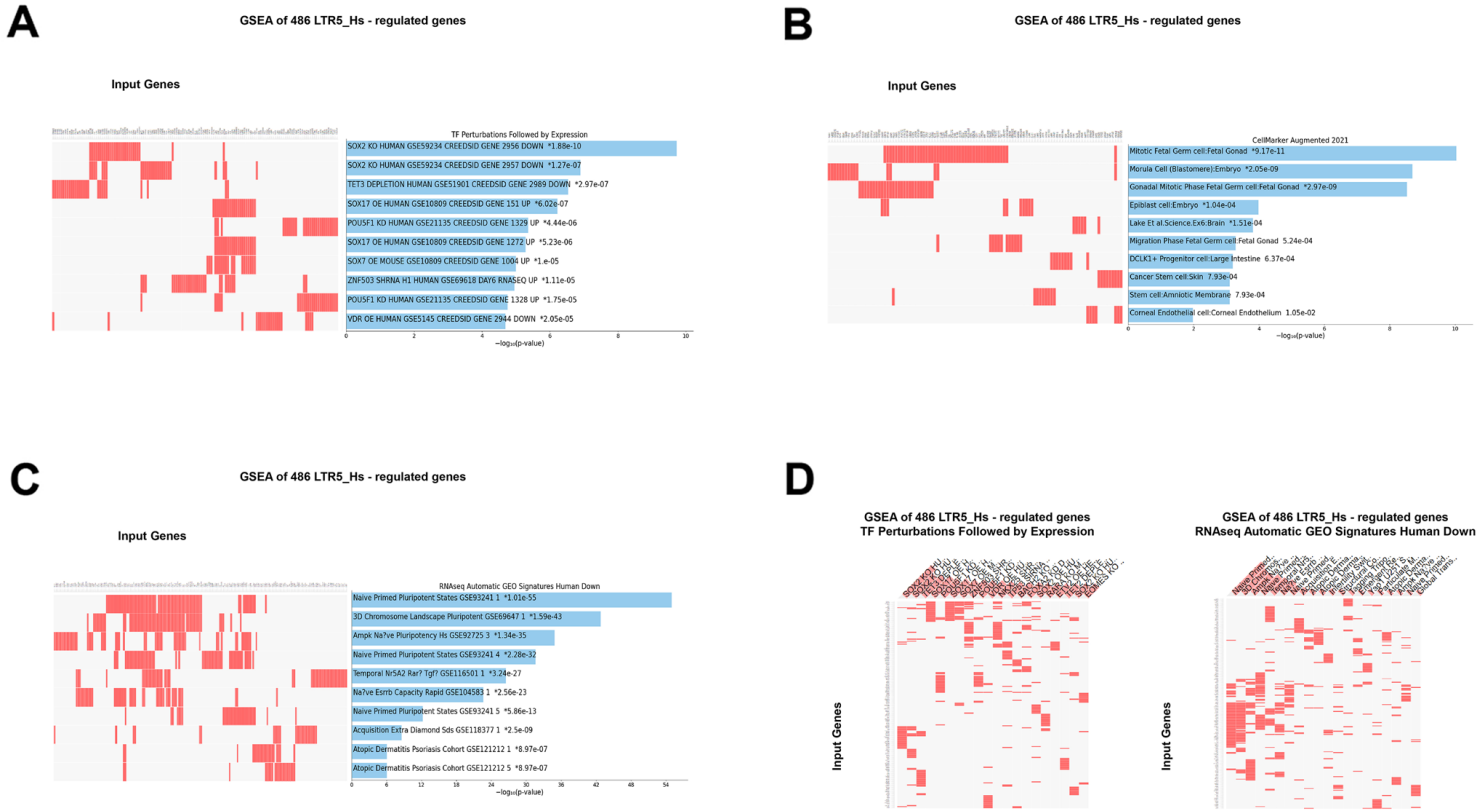
Assessments of potential phenotypic impacts of LTR5_Hs elements based on GSEA of 486 high-fidelity down-steam target genes employing the Transcription Factors (TF) perturbations followed by expression database (**A**; **D**, left panel), the Cell Marker Augmented 2021 database (**B**), the Human RNA seq Automatic GEO Signatures database (**C**; **D**, right panel). In panels **A**–**C** results illustrating the top 10 significantly enriched records are reported. In (D), top 30 significantly enriched records of gene sets are reported. All reported significantly enriched records were identified at the significance threshold of adjusted *p* value < 0.05 by the GSEA of 486 LTR5_Hs-regulated genes employing the corresponding genomic databases (Methods)

**Fig. 11:**
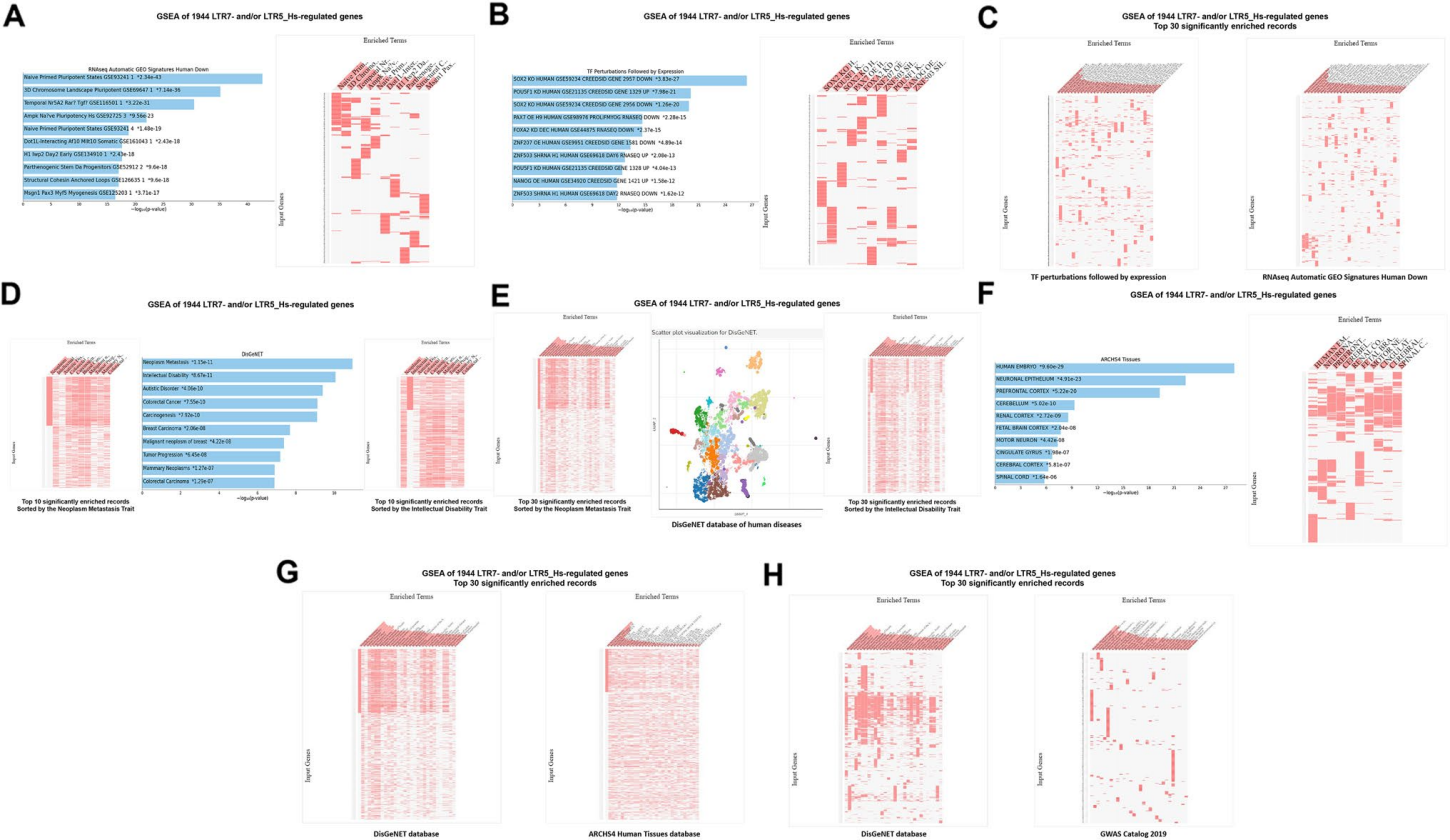
Potential phenotypic impacts of LTR7 and LTR5_Hs elements revealed by GSEA of 1944 high-fidelity down-steam target genes employing the Human RNA seq Automatic GEO Signatures database (**A**; **C**, right panel), the Transcription Factors (TF) perturbations followed by expression database (**B**; **C**, left panel), the DisGeNET database of human diseases (**D**–**H**, left panels), the ARCHS4 Human Tissues database (**F**; **G**, right panels), and the GWAS Catalog 2019 database (**H** right panel). In **D,** top 10 significantly enriched records of gene sets were sorted by genes comprising the expression signature of the Intellectual Disability trait (right panel) and genes comprising the expression signature of the Neoplasm Metastasis trait (left panel). In **E**, top 30 significantly enriched records of gene sets were sorted by genes comprising the expression signature of the Intellectual Disability trait (right panel) and genes comprising the expression signature of the Neoplasm Metastasis trait (left panel). Middle panel in **E** shows scatterplot visualization of GSEA results of the DusGeNET database of human diseases. In panels **A**, **B**, **D**, **F,** results illustrating the top 10 significantly enriched records are reported. In panels **C**, **E**, **G**, **H,** results illustrating the top 30 significantly enriched records are reported. All reported significantly enriched records were identified at the significance threshold of adjusted *p* value < 0.05 by the GSEA of 1944 genes regulated by LTR7- and/or LTR5_Hs loci employing the corresponding genomic databases (Methods)

**Fig. 12:**
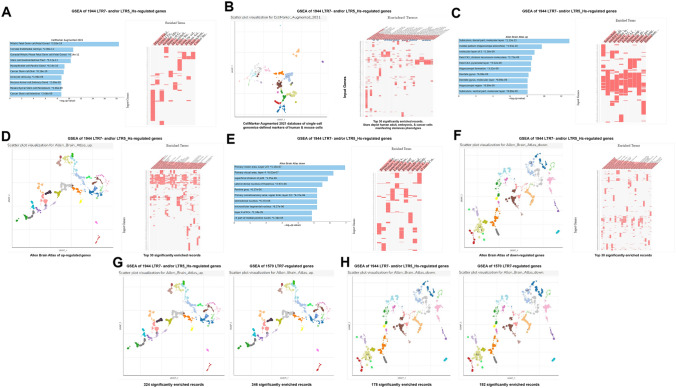
Potential phenotypic impacts of LTR7 and LTR5_Hs elements revealed by GSEA of 1944 high-fidelity down-steam target genes employing the Cell Marker Augmented 2021 database (**A**; **B**), the Allan Brain Atlas databases of up-regulated genes (**C**; **D**; **G**) and down-regulated genes (**E**; **F**; **H**). Left panel in **B** shows scatterplot visualization of GSEA results of the Cell Marker Augmented 2021 database of single-cell genomics-defined markers of human cells. Right panel in **B** reports top 30 significantly enriched records of gene sets identified by the GSEA of the Cell Marker Augmented 2021 database highlighting by stars gene expression signatures of human adult, embryonic, and cancer cells manifesting stemness phenotypes. Left panel in **D** shows scatterplot visualization of GSEA results of the Allan Brain Atlas database of up-regulated genes. Right panel in **D** reports top 30 significantly enriched records of gene sets identified by the Allan Brain Atlas database of up-regulated genes. Left panel in **F** shows scatterplot visualization of GSEA results of the Allan Brain Atlas database of down-regulated genes. Right panel in **F** reports top 30 significantly enriched records of gene sets identified by the Allan Brain Atlas database of down-regulated genes. Figure **G** shows side-by-side aligned scatterplots’ visualization of GSEA results of the 1944 LTR7- and/or LTR5_Hs-regulated genes (left panel; 324 significantly enriched records) and 1570 LTR7-regulated genes (right panel; 346 significantly enriched records) employing the Allan Brain Atlas database of up-regulated genes. Figure **H** shows side-by-side aligned scatterplots’ visualization of GSEA results of the 1944 LTR7- and/or LTR5_Hs-regulated genes (left panel; 178 significantly enriched records) and 1570 LTR7-regulated genes (right panel; 192 significantly enriched records) employing the Allan Brain Atlas database of down-regulated genes. In panels **A**, **C**, and **E,** results illustrating the top 10 significantly enriched records are reported. All reported significantly enriched records were identified at the significance threshold of adjusted *p* value < 0.05 by the GSEA of genes regulated by LTR7- and/or LTR5_Hs loci employing the corresponding genomic databases (Methods)

A majority of genes identified in this contribution as putative regulatory targets of LTR7 and/or LTR5_Hs loci manifests experimentally validated significant gene expression changes in response to targeted genetic or epigenetic manipulations (interference; silencing; or activation) of candidate up-stream regulatory LTRs (Tables 6, ,7 and 8). Therefore, these genes could be defined as high-confidence down-stream regulatory targets of LTR7 and/or LTR5_Hs loci. Taking into account these observations, it was of interest to evaluate potential biological impacts of LTR7 and LTR5_Hs loci on physiology and pathology of Modern Humans employing GSEA focused only on high-confidence down-stream regulatory target genes of LTR7 and LTR5_Hs elements. Results of these analytical experiments reinforce and extend reported above observations on biological roles and plausible pathophysiological effects of LTR7- and LTR5_Hs-linked down-stream regulatory target genes (Figs. 9, 10, 11 and 12; Supplementary Figures S3, S4; Supplementary Table S4).

Underscoring the important roles of LTR7 and LTR5_Hs loci in regulation of stemness and pluripotency state-related phenotypes, GSEA of 377 genes linked with both LTR7 and LTR5_Hs regulatory elements employing Human RNA seq Automatic GEO Signatures database identified among top 10 significantly enriched traits gene expression signatures of Naïve and Primed pluripotent states (*p* = 2.41E-09); chromatin-associated Sin3B protein-regulated quiescence (*p* = 1.27E-08); and pluripotent state 3D chromosome landscape (*p* = 6.40E-08) (Supplementary Figure S3A). Recapitulating, in part, phenotype-enrichment findings attributed to human-specific regulatory LTR elements (Fig. 5), GSEA of the DisGeNET database of human disorders identified Non-obstructive Y-linked Spermatogenic Failure (*p* = 1.32E-08); Male sterility due to Y chromosome deletions (*p* = 1.45E-05); Partial chromosome Y deletion (*p* = 1.45E-05); Oligospermia (*p* = 4.09E-05); and Chromic Alcoholic Intoxication (*p* = 6.07E-05) among top significantly enriched records (Supplementary Figures S3B; 3C). Schizophrenia (*p* = 2.40E-05) and Autism Spectrum Disorder (*p* = 3.55E-05) were scored as top 2 significantly enriched traits based on GSEA of the Disease Perturbations from GEO database focused on down-regulated genes (Supplementary Figure S3D). These observations indicate that despite distinct evolutionary histories separated by millions’ years of primates’ germlines colonization and expansion, genes representing down-stream regulatory targets of LTR7 and LTR5_Hs loci seem to exert the apparently cooperative phenotypic effects ascertained from the significantly enriched traits recorded by GSEA.

The apparent cooperative effects on phenotypic traits could be seeing when significantly enriched records attributed to 1570 high-confidence target genes of LTR7 loci (Fig. 9) and 486 high-confidence target genes of LTR5_Hs (Fig. 10) were compared for GSEA of the Transcription Factors’ (TF) perturbations followed by expression analyses database. These observations indicate that master transcriptional regulators of the pluripotency phenotype, namely SOX2, POU5F1, and NANOG, represent common up-stream TFs regulating expression of high-confidence regulatory target genes of both LTR7 and LTR5_Hs loci. This conclusion is supported by results of GSEA of 1944 genes comprising high-confidence down-steam regulatory targets of LTR7 and/or LTR5_Hs elements (Fig. 11) as evidenced by higher numbers of implicated signature genes and lower enrichment p values for down-stream targets of the SOX2 and POU5F1 TFs, which was recorded by GSEA of a cumulative set of 1944 down-stream target genes. Increased numbers of enriched signature genes and lower enrichment p values for a cumulative set of 1944 genes consisting of high-confidence down-stream regulatory targets of LTR7 and/or LTR5_Hs elements (Figs. 11 and 12) compared to gene sets linked with either LTR7 (Fig. 9) or LTR5_Hs (Fig. 10) loci were recorded for numerous significantly enriched phenotypic traits, including human embryo development stages, morphological components of the central nervous system (prefrontal cortex, cerebellum, fetal brain cortex, subiculum, hippocampal formation), and different cell types of fetal gonads. Exceptions from this trend were noted for GSEA of the Human RNA seq Automatic signatures database (Figs. 10C and 11A), indicating that down-stream target genes comprising expression signatures related to Naïve and Primed pluripotent states appear biased toward LTR5_Hs regulatory elements.

In several instances, GSEA of a cumulative set of 1944 genes consisting of high-fidelity down-stream regulatory targets of LTR7 and/or LTR5_Hs elements (Figs. 11 and 12) recorded the apparent qualitative differences compared to gene sets linked with either LTR7 (Fig. 9) or LTR5_Hs (Fig. 10) loci. For example, GSEA of the Cell Marker Augmented 2021 database revealed that human cells manifesting stemness phenotypes, including tissue-specific adult stem cells, embryonic stem cells, and cancer stem cells residing in various organs, consist a large majority (67%) of top 30 significantly enriched records (Fig. 12B). Similarly, increased numbers of up-regulated genes of dentate gyrus signatures and lower enrichment p values were recorded by GSEA of the Allen Brain Atlas database for a cumulative set of 1944 genes consisting of high-confidence down-stream regulatory targets of LTR7 and/or LTR5_Hs elements (Fig. 12C). However, the overall enrichment patterns of LTR high-confidence target genes identified by GSEA of the Allen Brain Atlas databases for both up-regulated (Fig. 12G) and down-regulated (Fig. 12H) genes appear driven by genes expression of which is regulated by LTR7 elements.

GSEA of 1944 genes comprising a cumulative set of high-confidence down-steam regulatory targets of LTR7 and/or LTR5_Hs loci revealed marked enrichment for genes implicated in neoplasm metastasis, intellectual disability, multiple cancer types, autism, Alzheimer’s, schizophrenia, and other brain disorders (Figs. 11 and 12). Similar to findings recorded for 562 LTR7-linked mammalian OS genes (Fig. 6), three most significantly enriched records of human diseases (Neoplasm metastasis; *p* = 1.15E-11; Intellectual disability; *p* = 6.67E-11; and Autistic disorder; p = 4.06E-10) appear associated with partially overlapping networks of genes (Fig. 11D, E), suggesting that transcriptional mis-regulation of high-confidence down-stream target genes of LTR7 and/or LTR5_Hs elements may contribute to pathogenesis of multiple types of human cancers and brain disorders.

### SARS-CoV-2 infection alters expression of a dominant majority of high-confidence LTR-target genes

One of noteworthy findings revealed by GSEA of 1944 genes comprising high-confidence down-steam regulatory targets of LTR7 and/or LTR5_Hs loci was a marked enrichment for genes expression of which is significantly altered in cells infected with the SARS-CoV-2 coronavirus, a pathogen causing the global COVID-19 pandemic. GSEA of high-confidence LTR-target genes employing the database of COVID-19-related Gene Sets 2021 identified 195 significantly enriched records of SARS-CoV-2-affected genes consisting of both up-regulated (48.4% of records) and down-regulated (51.6% of records) gene expression signatures (Supplementary Table S5). Follow-up analyses demonstrate that expression of a dominant majority (1696 of 1944 genes; 87%) of high-confidence LTR-target genes is altered in multiple types of SRAS-CoV-2 infected human cells and tissues as well as in SARS-CoV-2-infected cells from several other species, including Rhesus macaques, ferrets, hamsters, and mice. In contrast, only 4% of LTR-linked genes expression of which was not directly influenced by LTRs were affected in SARS-CoV-2-infected cells. These observations indicate that gene expression signatures of a cellular response to SARS-CoV-2 infection constitute a dominant majority of high-confidence LTR-target genes.

To determine whether infections by other viruses alter the expression of LTR-regulated genes, GSEA of 1944 high-confidence LTR-target genes were carried out employing Virus Perturbations from GEO databases of up-regulated and down-regulated genes manifesting significant expression changes in response to viral infections (Supplementary Table S5). Results of these analytical experiments demonstrate that expression of 1281 LTR-regulated genes is altered in response to infections by 14 different viruses (Supplementary Table S5). Notably, expression of a dominant majority of these genes (1164 of 1281 genes; 91%) is significantly altered in SARS-CoV-2-infected cells. Overall, these observations revealed that expression of 1814 of 1944 (93%) high-confidence LTR-target genes is altered in virus-infected cells, suggesting that gene expression signatures of cellular responses to encounters with viruses are embedded, in part, within genomic regulatory networks (GRN) governed by retroviral LTR elements.

### Genes implicated in differentiation of human cells and tissues constitute a significant majority of high-confidence down-stream targets of LTR elements

**Fig. 13:**
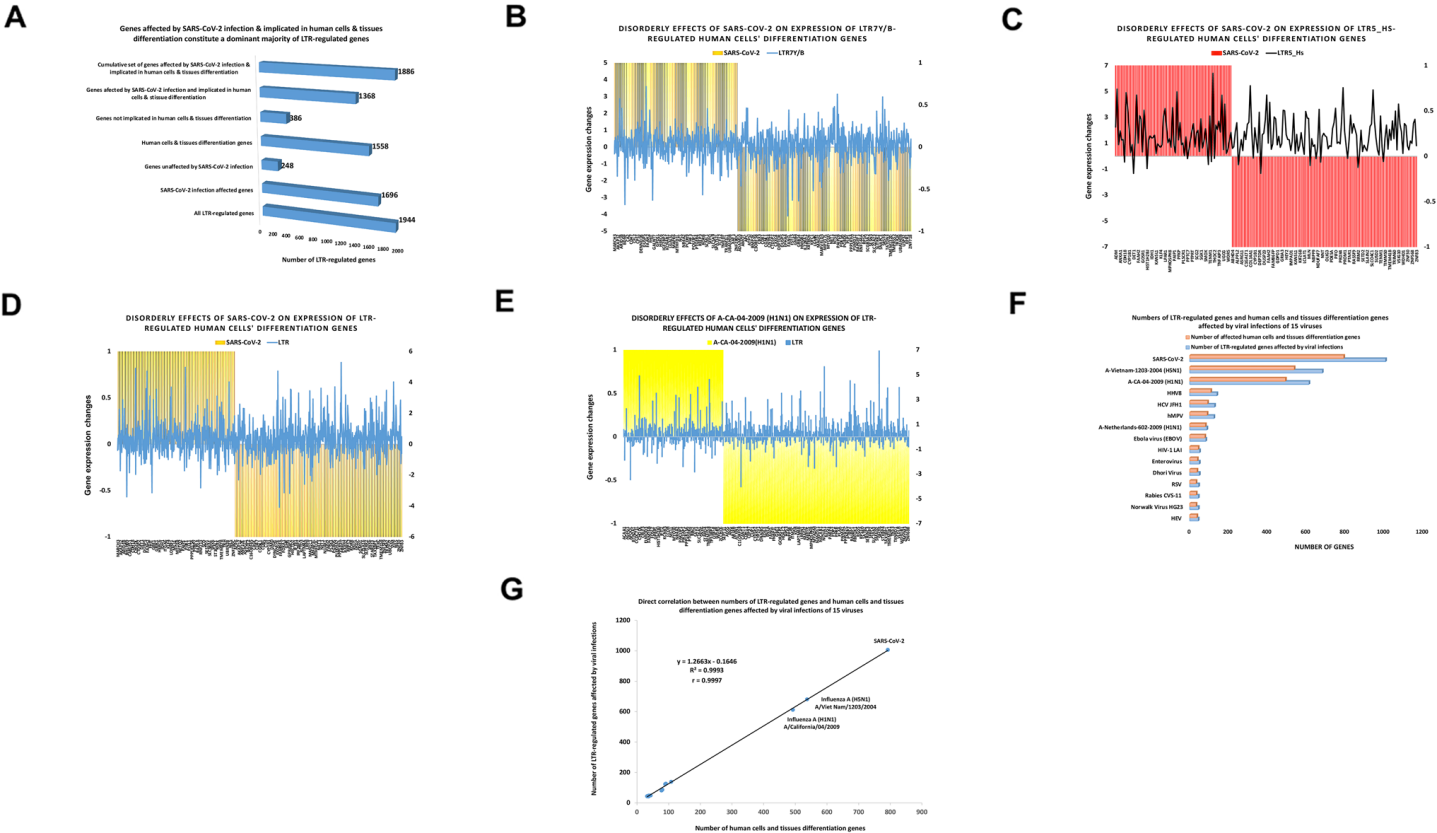
A dominant majority of high-confidence LTR-regulated down-stream target genes constitutes genes expression of which is affected by SARS-CoV-2 infection and defined as genetic markers of differentiation of multiple types of human cells and tissues. Panel **A** reports the number of genes expression of which is affected by SARS-CoV-2 infection (1696 genes); the number of genes defined as genetic markers of differentiation of human cells and tissues (1558 genes); the number of genes assigned to both viral infection and cellular differentiation categories (1368 genes); and the cumulative number of genes identified as SARS-CoV-2 infection targets and/or human cells’ and tissues’ differentiation genes (1886 genes) among 1944 genes comprising high-confidence down-stream regulatory targets of retroviral LTRs. Panels **B**–**E** illustrate disorderly effects of SARS-CoV-2 infection (**B**–**D**) and influenza H1N1 strain A-CA-04-2009 (H1N1) infection (**E**) on expression of LTR-regulated human cells’ and tissues’ differentiation genes. Panels **F**, **G** summarize the experimental evidence documenting a strict direct correlation between the numbers of LTR-regulated human cells’ and tissues’ differentiation genes and the numbers of LTR-regulated genes expression of which is significantly affected by viral infections of 15 different viruses

Results of GSEA of high-confidence down-stream regulatory target genes of LTR elements demonstrate that GRN governed by endogenous retroviral LTRs are significantly enriched for genes expression of which distinguishes multiple human cells and tissues of an exceedingly broad development spectrum, including embryogenesis, post-embryonic development, and adulthood (Figs. 11 and 12; Table 9). These findings indicate that transcriptional control of human cells and tissues differentiation programs may constitute one of the principal biological functions of GRN governed by retroviral LTRs. In accord with this hypothesis, a significant majority of high-confidence down-stream regulatory targets of retroviral LTR elements (1558 of 1994 genes; 80%) is represented by gene expression signatures (GES) of human cells’ and tissues’ differentiation. Notably, 1368 of 1944 LTR-regulated genes (70%) represent both SARS-CoV-2 infection-affected genes and genes implicated in differentiation of human cells and tissues. Consequently, expression of a dominant majority (1368 of 1558 genes; 88%) of genes comprising human cells’ and tissues’ differentiation signatures appears altered in SARS-CoV-2-infected cells. Conversely, a large fraction of genes (1368 of 1696 genes; 81%) expression of which is affected by SARS-CoV-2 infection represents genes implicated in differentiation of human cells and tissues. Overall, a cumulative set of genes identified as either SARS-CoV-2 infection-affected genes or human cells’ and tissues’ differentiation genes constitutes nearly all high-confidence down-stream regulatory target genes of retroviral LTR elements (1886 of 1944 genes; 97%). These observations strongly imply that one of important pathophysiological mechanisms underlying pathological effects of SARS-CoV-2 infection on human body may constitute the molecular interference with differentiation gene expression programs of multiple cells and tissues (Table 9). Consistent with this hypothesis, gene expression profiling experiments revealed that SARS-CoV-2 infection causes disorderly effects on expression of LTR-regulated genes implicated in differentiation of human cells and tissues (Fig. 13).

**Table 9:** Enrichment patterns of phenotypic traits affected by genes comprising high-confidence down-stream regulatory targets of LTR7 and LTR5_Hs loci

Classification category	LTR-regulated genes	SARS-CoV-2 affected genes	Human cells and tissues differentiation genes
Number of genes	1944	1696	1558
Percent	100.00	87.24	80.14
Significantly enriched records of phenotypic traits identified by the GSEA of genomic database			
DisGeNET database	122	202	212
PanglaoDB augmented 2021	62	53	71
CellMarker augmented 2021	205	228	262
Azimuth cell types 2021	10	5	76
ARCHS4 human tissues	34	33	60
Allen brain atlas up	324	308	411
Allen brain atlas down	178	260	303
GTEx tissue expression up	564	698	775
GTEx tissue expression down	455	517	324
COVID-19-related gene sets 2021	195	264	175
Virus perturbations from GEO up*	49 (17)	66 (22)	2 (1)
Virus perturbations from GEO down*	58 (25)	72 (28)	42 (13)
MSigDB oncogenic signatures	47	53	53
Disease perturbations from GEO up	218	323	212
Disease perturbations from GEO down	238	314	254
MSigDB hallmark 2020	24	26	21
GWAS catalog 2019	24	30	41
Significantly enriched records of phenotypic traits identified by the GSEA of human rare diseases databases			
Rare diseases GeneRIF gene lists	7	59	40
Rare diseases AutoRIF gene lists	51	174	38
Rare diseases AutoRIF ARCHS4 predictions	48	72	104
Rare diseases GeneRIF ARCHS4 predictions	46	56	89
Significantly enriched records of phenotypic traits identified by the GSEA of targeted TF perturbations, TF PPI, and PPI hub proteins genomic database			
TF perturbations followed by expression	833	1078	790
Transcription factor PPIs	0	0	1
PPI hub proteins	1	15	0

Reported numbers indicate the quantity of significantly enriched records (*p* adj < 0.05) identified by GSEA of corresponding genomics and proteomics databases. Analyzed gene sets include the parent set of 1944 LTR-regulated genes; a sub-set of 1696 LTR-regulated genes expression of which is affected by SARS-CoV-2 infection; and a sub-set of 1558 LTR-regulated genes comprising differentiation GES of human cells and tissues

*Numbers in parenthesis report numbers of significantly enriched records (*p* adj < 0.05) attributed to SARS-CoV-2 infections

Similar to SARS-CoV-2 infection, infections caused by 14 other viruses significantly changed expression of human cells’ and tissues’ differentiation genes, which constitute from 71% (RSV virus) to 94% (Ebola virus) of LTR-regulated genes affected by viral infections (Fig. 13; Supplementary Table S5). For gene expression changes caused by viral infections, the apparently firm association was observed between numbers of affected LTR-regulated genes and human cells’ and tissues’ differentiation genes, which is exemplified by the strict correlation (*R*^2^ = 0.999) between sets of these independently defined experimental endpoints for 15 different viruses (Fig. 13; Supplementary Table S5).

## Discussion

### Insights from LTR family-specific and granular analyses of evolutionary origin, expansion, conservation, and divergence of LTR7 and LTR5_Hs elements during primate evolution

In this contribution, DNA sequences conservation analyses of most recent releases of 17 primate species’ genomes were employed to identify genomes manifesting the consistent presence of highly conserved (HC) orthologous retroviral LTR sequences at the earliest time points during primate evolution. Results of these analyses were utilized to infer the timelines of initial colonization and subsequent expansion of human endogenous retroviruses (HERV) LTR7/HERVH and LTR5_Hs/HERVK among primate species. LTR7/HERVH and LTR5_Hs/HERVK retroviruses appear to have distinct evolutionary histories of successful colonization of primates’ genomes charted by evidence of the earliest consistent presence and expansion of HC LTR sequences. In contrast to genomes of New World Monkeys, genomes of the Old World Monkey lineage consistently harbor ~ 18% of HC-LTR7 loci residing in genomes of Modern Humans. These observations suggest that LTR7/HERVH have entered germlines of primate species in Africa after the separation of the New World Monkey lineage. The earliest presence of HC-LTR5_Hs loci has been identified in the Gibbon’s genome (24% of HC-LTR5_Hs loci residing in human genomes), suggesting that LTR5_Hs/HERVK successfully colonized primates’ germlines after the segregation of Gibbons’ species. Subsequently, both LTR7 and LTR5_Hs underwent a marked ~ fourfold–fivefold expansion in genomes of Great Apes. Intriguingly, timelines of quantitative expansion of both LTR7 and LTR5_Hs loci during evolution of Great Apes appear to replicate the consensus evolutionary sequence of increasing cognitive and behavioral complexities of non-human primates, which seems particularly striking for LTR7 loci and 8 distinct LTR7 subfamilies.

Discovery of a complex polyphyletic composition of LTR7 elements comprising at least eight monophyletic subfamilies (Carter et al. 2022) prompted sequence conservation analyses of each individual monophyletic subfamilies of LTR7 loci in genomes of sixteen NHP. Results of these analyses suggest that diversification of LTR7 loci into genetically and regulatory distinct subfamilies may have occurred early during primate evolution and subsequent cycles of LTR7 expansion appear to faithfully maintain this diversity. This conclusion is supported by observations that highly conserved sequences of all monophyletic LTR7 subfamilies are present in genomes of all analyzed in this study Old World Monkeys’ species as well as in genomes of Gibbon, Orangutan, Gorilla, Bonobo, and Chimpanzee (Fig. 2). Despite large differences in the numbers of highly conserved LTR7 loci among different primate species, the overall quantitative balance of distinct monophyletic LTR7 subfamilies appears maintained very tightly during millions’ years of primate evolution, which is reflected by nearly perfect correlations of LTR7 subfamilies abundance profiles between evolutionary closely related primate species (Fig. 3). On a 40 MYA scale of primate evolution from Old World Monkeys to Modern Humans, the phenomenon of conservation of LTR7 subfamilies abundance profiles is illustrated by the strong inverse correlation (*r* = – 0.986) of a degree of resemblance of NHP species’ and Modern Humans LTR7 subfamilies abundance profiles and estimated divergence time from ECA (Fig. 3C).

One of the notable features of LTR7 sequences conservations analyses in genomes of five Old World Monkeys’ species was strikingly similar numbers of gains and losses of LTR7 loci independently estimated for each species (Fig. 1). Using these data as a baseline for estimates of numbers of LTR7 loci gains per MYA during primate evolution (Fig. 1D), a model of a putative species-specific expansion of regulatory LTR7 loci during primate evolution was built for genomes of eleven NHP (Fig. 1C). For each species, relative gains (a number of highly conserved LTR7 loci identified in a genome) and losses (a deficit of highly conserved LTR7 loci compared to genomes of Modern Humans) of LTR7 loci were calculated vis-a-vis Modern Human’s genome housing 3354 highly conserved LTR7 elements (Fig. 1C). Based on these estimates tailored to a presumed timeline of Old World Monkeys’ segregation from ECA, a hypothetical model defining primate species segregation timelines from ECA was developed, which implies putative direct associations of LTR7 loci acquisitions in primates’ genomes with timelines of species segregation processes during primate evolution (Fig. 1D). It will be of interest to determine whether this apparent association might reflect the causative impacts of LTR7/HERVH expansions on the emergence and segregation of primate species.

### Insights from analyses of potential phenotypic impacts of LTR7 and LTR5_Hs elements on physiology and pathology of Modern Humans

Extensive investigations of LTR7 elements conclusively documented their regulatory functions and locus-specific differential expression in human preimplantation embryogenesis as well as in human embryonic and pluripotent stem cells (Fort et al. 2014; Gemmell et al. 2015; Glinsky et al. 2018; Göke et al. 2015; Izsvák et al. 2016; Kelley and Rinn, 2012; Loewer et al. 2010; Lu et al. 2014; Ohnuki et al. 2014; Pontis et al. 2019; Römer et al. 2017; Santoni et al. 2012; Takahashi et al. 2021; Theunissen et al. 2016; Wang et al. 2014; Zhang et al. 2019). Similarly, LTR5_Hs/HERVK-derived loci manifest transcriptional and biological activities in human preimplantation embryos and in naïve hESCs (Grow et al. 2015). Many facets of activities of LTR5_Hs elements were attributed to acquisition of enhancer-like chromatin state signatures concomitantly with transcriptional reactivation of HERVK sequences (Grow et al. 2015), consistent with LTR5_Hs elements acting as distal enhancers exerting global long-range effects on expression of thousands human genes (Fuentes et al. 2018). Thus, our understanding of LTR7 and LTR5_Hs functions was restricted to a large degree to preimplantation embryogenesis, hESC, and pluripotent stem cells. Results of analytical experiments carried out in this contribution strongly argue that LTR7 and LTR5_Hs elements may affect many previously underappreciated aspects of physiological functions and pathological conditions of Modern Humans.

Inferences of potential phenotypic effects of LTR7 and LTR5_Hs elements were based on assessments of experimentally validated biological functions and cell-type specific differential expression profiles of genes identified as down-stream regulatory targets of LTR7 and LTR5_Hs loci. To ensure the high-stringency definition of candidate down-stream regulatory targets and reduce the likelihood of spurious associations, only significantly enriched records of gene signatures and linked phenotypic traits identified by GSEA at the significance threshold of adjusted *p* value < 0.05 and/or FDR *q* value < 0.05 were considered. Importantly, all observations that have been considered as strong evidence of implied biological effects of LTR7 and LTR5_Hs loci were validated by GSEA of genes experimentally defined as down-stream regulatory targets of LTR7 and LTR5_Hs elements. Confirming the validity of this analytical approach, the important roles of LTR7 and LTR5_Hs loci in regulation of preimplantation embryogenesis, stemness, and pluripotency state-related phenotypes have been documented for both LTR7 and LTR5_Hs regulatory elements.

One of the most intriguing findings reported herein is the postulated regulatory effect of human-specific LTR7 and LTR5_Hs loci on genes encoding markers of 12 distinct cells’ populations of fetal gonads, as well as genes implicated in physiology and pathology of human spermatogenesis, including Y-linked spermatogenic failure, oligo- and azoospermia. Identified in this contribution readily available well-characterized mouse models conclusively linking genes and phenotypes of interest should facilitate the experimental testing of the validity of this hypothesis.

Mammalian offspring survival (MOS) genes have been identified as one of consistent regulatory targets throughout ~ 30 MYA of the divergent evolution of LTR7 loci. Significantly, differential GSEA of LTR-linked MOS versus non-MOS genes identified dominant enrichment patterns of phenotypic traits affected by 562 LTR7-regulated and 126 LTR5_Hs-regulated MOS genes. Specifically, GSEA of LTR7-linked MOS genes identified more than 2200 significantly enriched records of human common and rare diseases and 466 significantly enriched records of Human Phenotype Ontology traits, including 92 genes of Autosomal Dominant Inheritance and 93 genes of Autosomal Recessive Inheritance. It will be of interest to test experimentally whether regulatory effects on MOS genes could be one of contributing genetic determinants driving species fitness and divergence during primate evolution.

One of the most consistent observations documented by interrogations of LTR7-linked down-stream target genes was a clear prevalence of enrichment records related to brain and CNS functions among significantly enriched phenotypic traits identified by GSEA of genomic databases focused on gene expression signatures of tissues and cell types across human body. For instance, GSEA of the single-cell sequencing PanglaoDB Augmented 2021 database identified significantly enriched records of gene signatures representative of cells of distinct neurodevelopmental stages and morphologically diverse cell types residing and functioning in human brain, including Neural Stem/Precursor cells, Radial Glia cells, Bergman Glia cells, Pyramidal cells, Tanycytes, Immature neurons, Interneurons, Trigeminal neurons, GABAergic neurons, and Glutamatergic neurons. GSEA of LTR7-linked down-stream target genes employing the Allen Brain Atlas database identified 521 significantly enriched records of different human brain regions harboring expression signatures of both up-regulated (420 brain regions) and down-regulated (101 brain regions) genes.

These observations indicate that LTR7-linked down-stream target genes may contribute to multiple facets of development and functions of human brain. In-depth analyses of LTR7 and LTR5_Hs loci linked with down-stream target genes affecting synaptic transmission and protein–protein interactions at synapses provide further evidence supporting this hypothesis.

One of important conclusions that could be derived from present analyses is that despite clearly distinct evolutionary histories of LTR7/HERVH and LTR5_Hs/HERVK retroviruses separated in time by millions of years, genes representing down-stream regulatory targets of LTR7 and LTR5_Hs loci exert the apparently cooperative and exceedingly broad phenotypic impacts on physiology and pathology of Modern Humans. Considering distinct patterns of retroviral insertions during the initial stages of genome colonization and expansion, it would be of interest to determine how this cooperative phenotypic impacts have been attained and what the role of natural selection is in the alignment of phenotypic effects of distinct retroviral families.

## Conclusion

Repetitive DNA sequences, including transposons and retroviral LTRs, represent one of the principal layers of high-complexity genomic governance connectivity codes designed to seed genome-wide grids of regulatory DNA elements to enable unified cellular responses to a variety of endogenous and exogenous cues (Britten and Davidson 1971; Glinsky 2009). Streamlined unified cellular responses are facilitated by coordinated changes of transcriptional outputs at hundreds (perhaps, thousands) spatially segregated genomic loci harboring binding sites for transcriptions factors (TFs) and chromatin remodelers (Britten and Davidson 1971; Ito et al. 2017). These properties of retroviral LTRs empower their crucial contributions during evolution to creation of species-specific features of signal transduction pathways associated with defined genomic regulatory networks (GRNs).

To successfully colonize host genomes on a population scale, retroviruses must infect the germline during the early embryogenesis prior to or at the stage of the germ cells’ biogenesis, thus facilitating the propagation and stable integration of multiple copies of viral genomes into host chromosomes and ensuring the passage of integrated viral sequences to offsprings. Therefore, there is a relatively small window of the developmental timeline which represents a required target for viral infections to enable successful transitions of exogenous retroviruses to the state of endogenous retroviruses (ERVs) integrated into host genomes. Significantly, integration of retroviruses in the human genome favor active genes and integrated retroviruses appear preferentially detected in the open chromatin regions representing hallmarks of transcriptionally active genomic loci (Schröder et al. 2002; Cereseto and Giacca 2004; Bushman et al. 2005). It follows that ERVs colonizing the human genome during the relatively narrow embryonic development window may target functionally related panels of genes and developmental pathways, thus potentially affecting common sets of phenotypic traits. Results of the analyses reported in this contribution support the model of cooperative phenotypic impacts on human pathophysiology exerted by genes representing down-stream regulatory targets of LTR7 and LTR5_Hs elements, despite their markedly distinct evolutionary histories of the human genome colonization spanning millions of years.

Present analyses revealed that expression of genes comprising down-stream regulatory targets of retroviral LTRs is altered following genetic targeting of TF-coding genes, indicating that identified herein retroviral LTR-regulated genes may function in human cells as down-stream targets of GRNs governed by hundreds of regulatory interactions mediated by host TFs and PPI Hub proteins. This functional integration of retroviral LTR’s down-stream target genes into GRNs governed by host TFs and PPI Hub proteins is likely to occur at developmental stages when retroviral LTR’s activity is epigenetically silenced and processes of cells and tissues differentiation ensued. Consistent with this hypothesis, a significant majority of genes (1558 of 1994 genes; 80%) comprising high-confidence down-stream regulatory targets of retroviral LTRs have been implicated in differentiated states of exceedingly broad spectrum of human cells and tissues. Unexpectedly, expression of a dominant majority (1368 of 1558 genes; 88%) of LTR-target genes comprising human cells’ and tissues’ differentiation signatures appears altered in SARS-CoV-2-infected cells, suggesting that one of the mechanisms of pathogenic actions of the SARS-CoV-2 coronavirus is the interference with differentiation programs of multiple types of human cells and tissues. Follow-up analyses demonstrate that infections caused by 14 other viruses significantly changed expression of human cells’ and tissues’ differentiation genes comprising regulatory targets of retroviral LTRs residing in the human genome. Overall, expression of 1814 of 1944 (93%) high-confidence LTR-regulated genes is altered in virus-infected cells, consistent with the idea that gene expression signatures of cellular responses to contemporary viral infections are converged, in part, on GRNs governed by ancient retroviral LTR elements.

Several lines of observations reported in this study highlight putative mechanisms by which retroviral regulatory LTRs may have affected phenotypic traits contributing to species segregation during primate evolution and development of human-specific phenotypic features. Among intriguing findings of this category are discoveries of potential regulatory impacts of retroviral LTRs residing in the human genome on expression of mammalian offspring survival (MOS) genes as well as regulatory effects of human-specific LTR7 and LTR5_Hs loci on expression of genes encoding markers of 12 distinct cells’ populations of fetal gonads and genes implicated in pathophysiology of human spermatogenesis, including Y-linked spermatogenic failure, oligo- and azoospermia. Most recently, direct experimental evidence linking retroviral LTR loci (LTR7Y and LTR5_Hs) to gene regulatory networks shared between human primordial germ cells and naïve pluripotent cells has been reported (Ito et al. 2022). Observations documented in this contribution indicate that LTR7-linked down-stream target genes may contribute to multiple facets of development and functions of hundreds anatomically distinct regions of human brain. Follow-up analyses of LTR7 and LTR5_Hs loci demonstrated their potential regulatory effects on down-stream target genes affecting synaptic transmission and protein–protein interactions at synapses.

Collectively, observations reported in this contribution highlight LTR7 and LTR5_Hs regulatory elements as important genomic determinants of Modern Humans’ health and disease states, which exert their phenotypic impacts via effects on down-stream target genes across the stages of human lifespan from preimplantation embryogenesis throughout pre- and post-natal developmental periods to adulthood and aging.

## Supplementary Information

Below is the link to the electronic supplementary material.

Supplementary Figure S1 (PPTX 814 KB)

Supplementary Figure S2 (PPTX 440 KB)

Supplementary Figure S3 (PPTX 717 KB)

Supplementary Figure S4 (PPTX 1777 KB)

Supplementary Table S1 (XLSX 9 KB)

Supplementary Table S2 (XLSX 23675 KB) 

Supplementary Table S3 (XLSX 577 KB)

Supplementary Table S4 (XLSX 289 KB)

Supplementary Table S5 (XLSX 6805 KB)
