# Supplementary material for: Molecular diversity and phenotypic pleiotropy of ancient genomic regulatory loci derived from human endogenous retrovirus type H (HERVH) promoter LTR7 and HERVK promoter LTR5_Hs and their contemporary impacts on pathophysiology of Modern Humans
Source: Mol Genet Genomics. 2022 Sep 19;297(6):1711–40. doi: 10.1007/s00438-022-01954-7 (PMC9483895; doi:10.1007/s00438-022-01954-7)
Supplement: Supplementary file 3 — Supplementary Figure S3 (PPTX 717 KB) [file 438_2022_1954_MOESM3_ESM.pptx]

## Slide 1
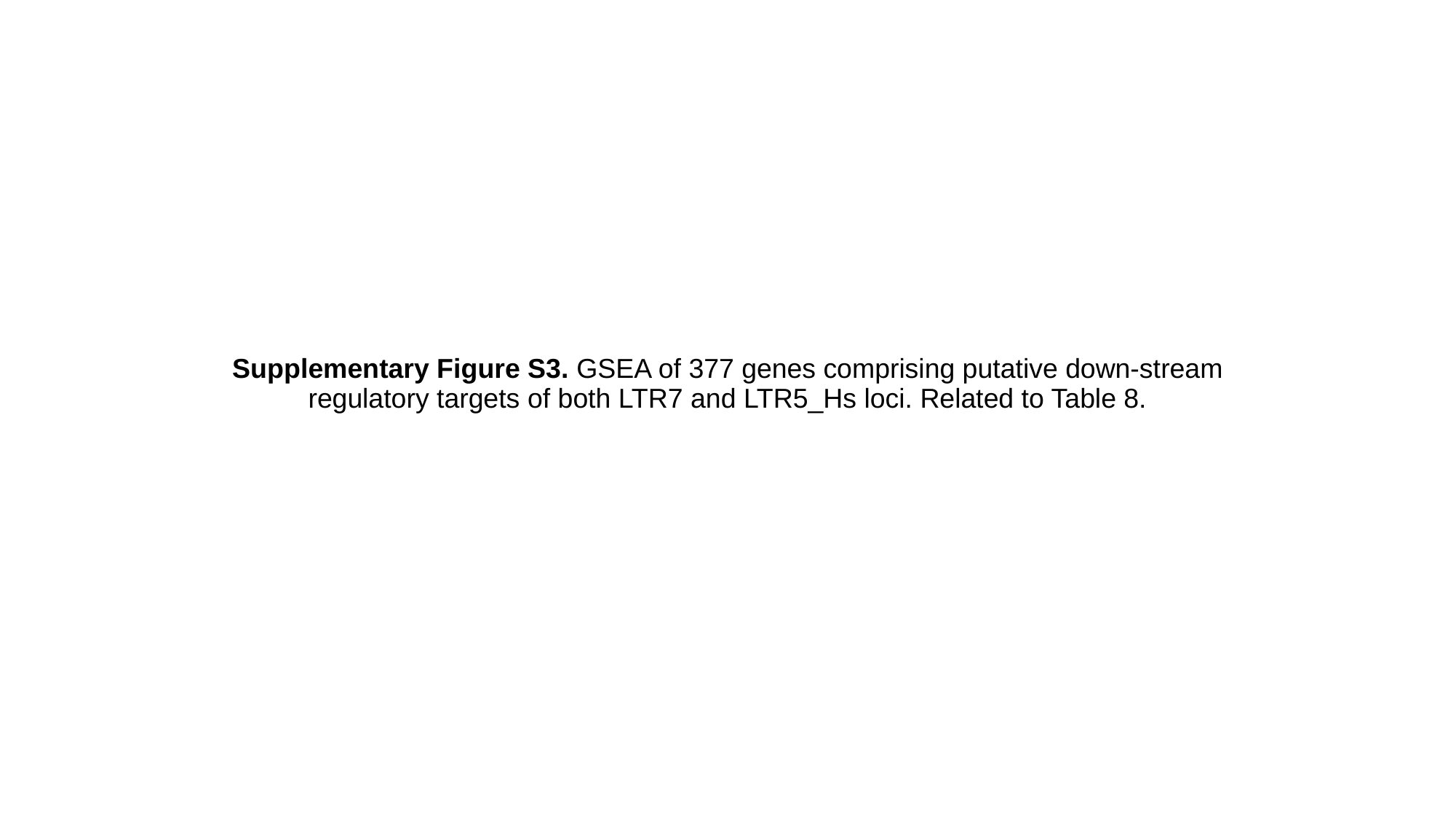

# Supplementary Figure S3. GSEA of 377 genes comprising putative down-stream regulatory targets of both LTR7 and LTR5_Hs loci. Related to Table 8.

## Slide 2
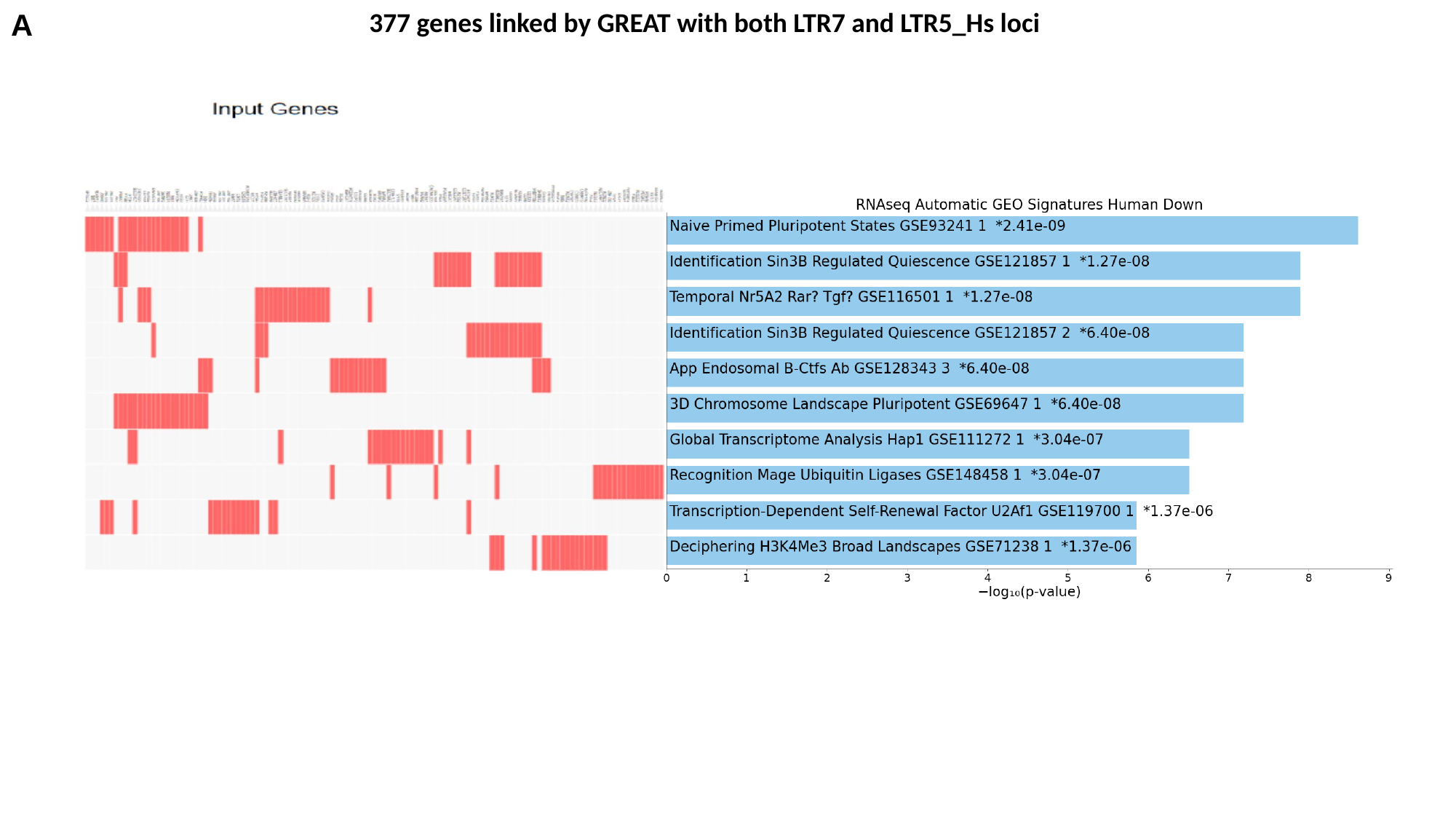

A
377 genes linked by GREAT with both LTR7 and LTR5_Hs loci

## Slide 3
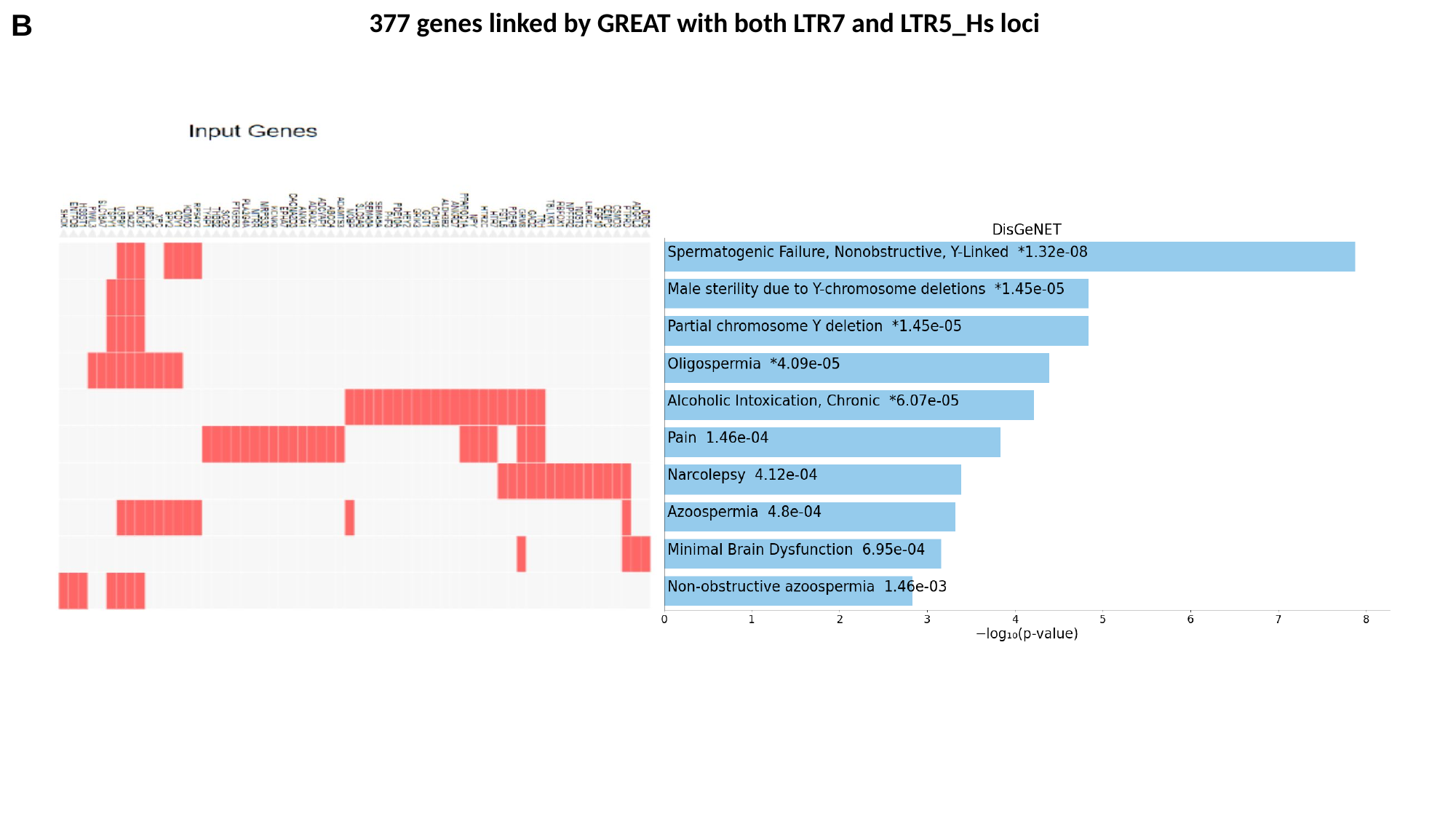

B
377 genes linked by GREAT with both LTR7 and LTR5_Hs loci

## Slide 4
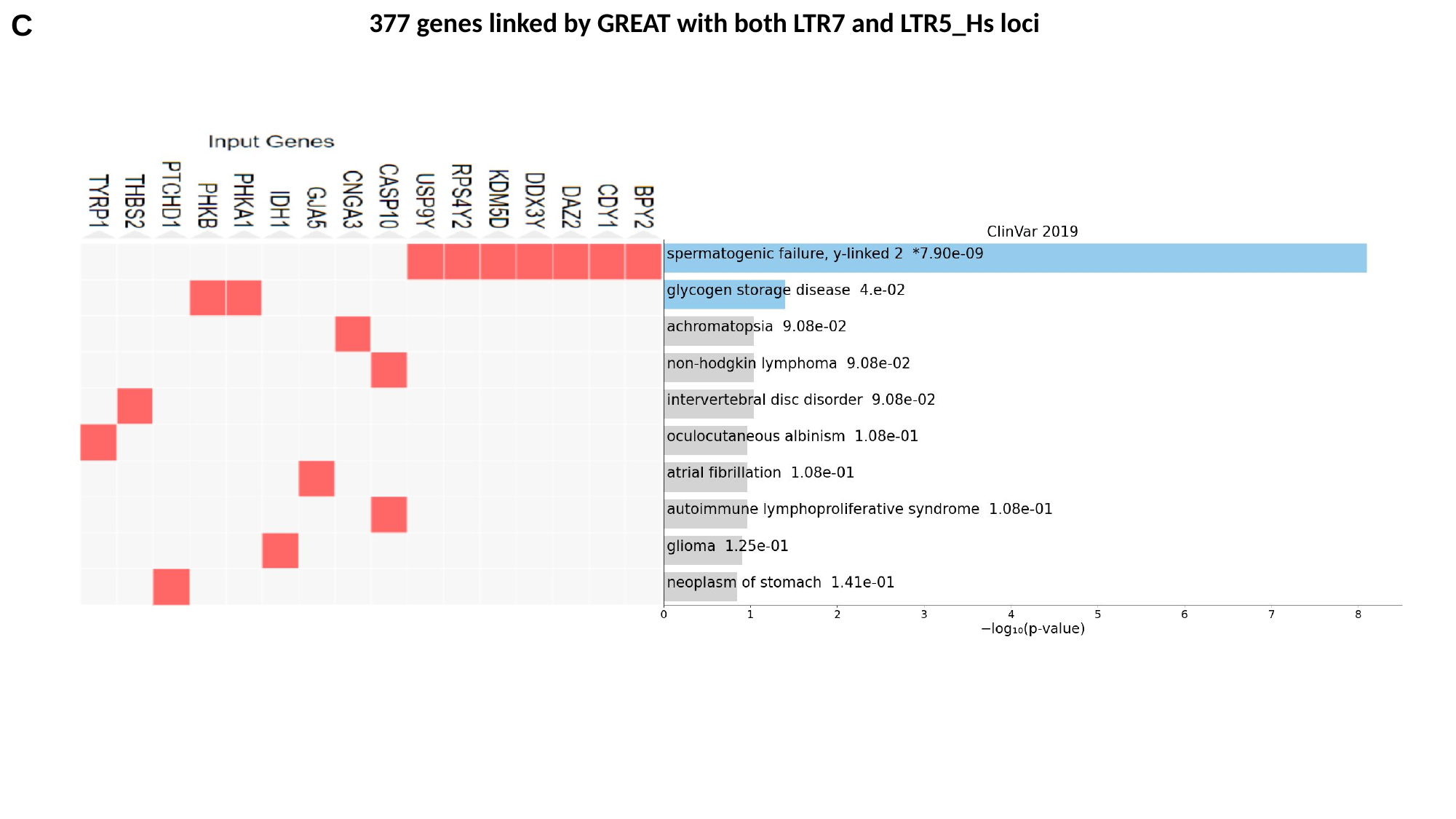

C
377 genes linked by GREAT with both LTR7 and LTR5_Hs loci

## Slide 5
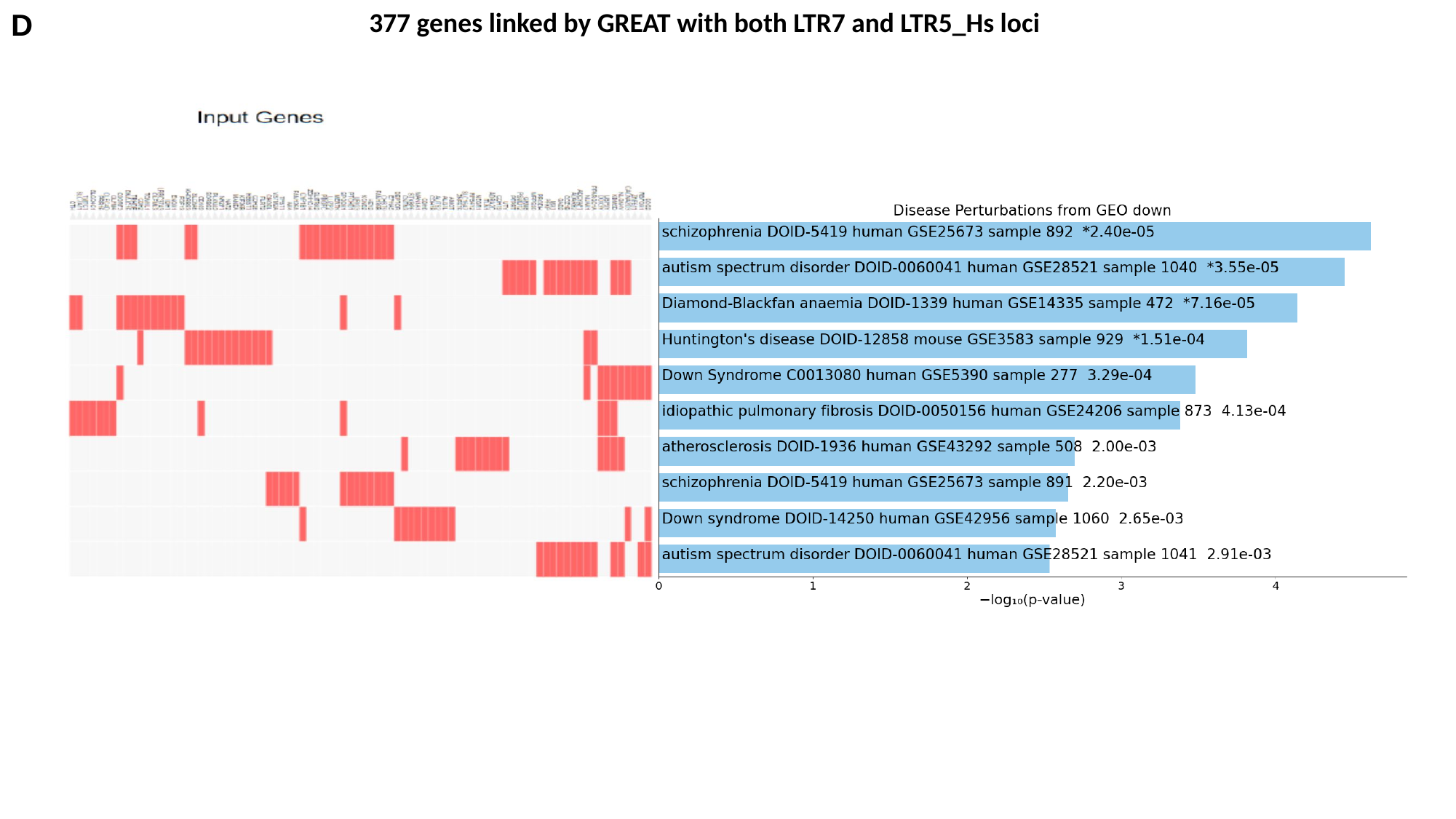

D
377 genes linked by GREAT with both LTR7 and LTR5_Hs loci
