# Supplementary material for: Molecular diversity and phenotypic pleiotropy of ancient genomic regulatory loci derived from human endogenous retrovirus type H (HERVH) promoter LTR7 and HERVK promoter LTR5_Hs and their contemporary impacts on pathophysiology of Modern Humans
Source: Mol Genet Genomics. 2022 Sep 19;297(6):1711–40. doi: 10.1007/s00438-022-01954-7 (PMC9483895; doi:10.1007/s00438-022-01954-7)
Supplement: Supplementary file 4 — Supplementary Figure S4 (PPTX 1777 KB) [file 438_2022_1954_MOESM4_ESM.pptx]

## Slide 1
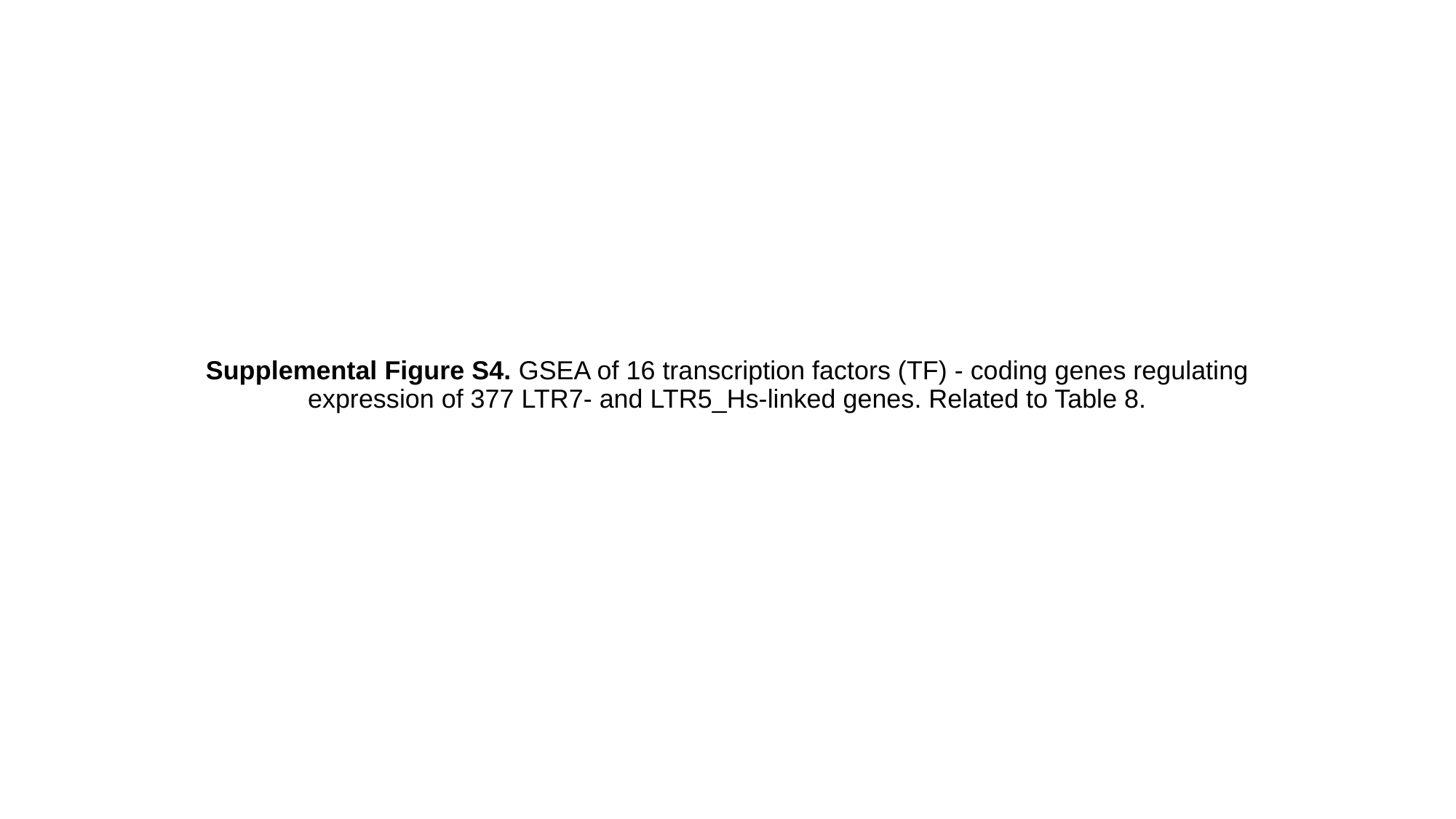

# Supplemental Figure S4. GSEA of 16 transcription factors (TF) - coding genes regulating expression of 377 LTR7- and LTR5_Hs-linked genes. Related to Table 8.

## Slide 2
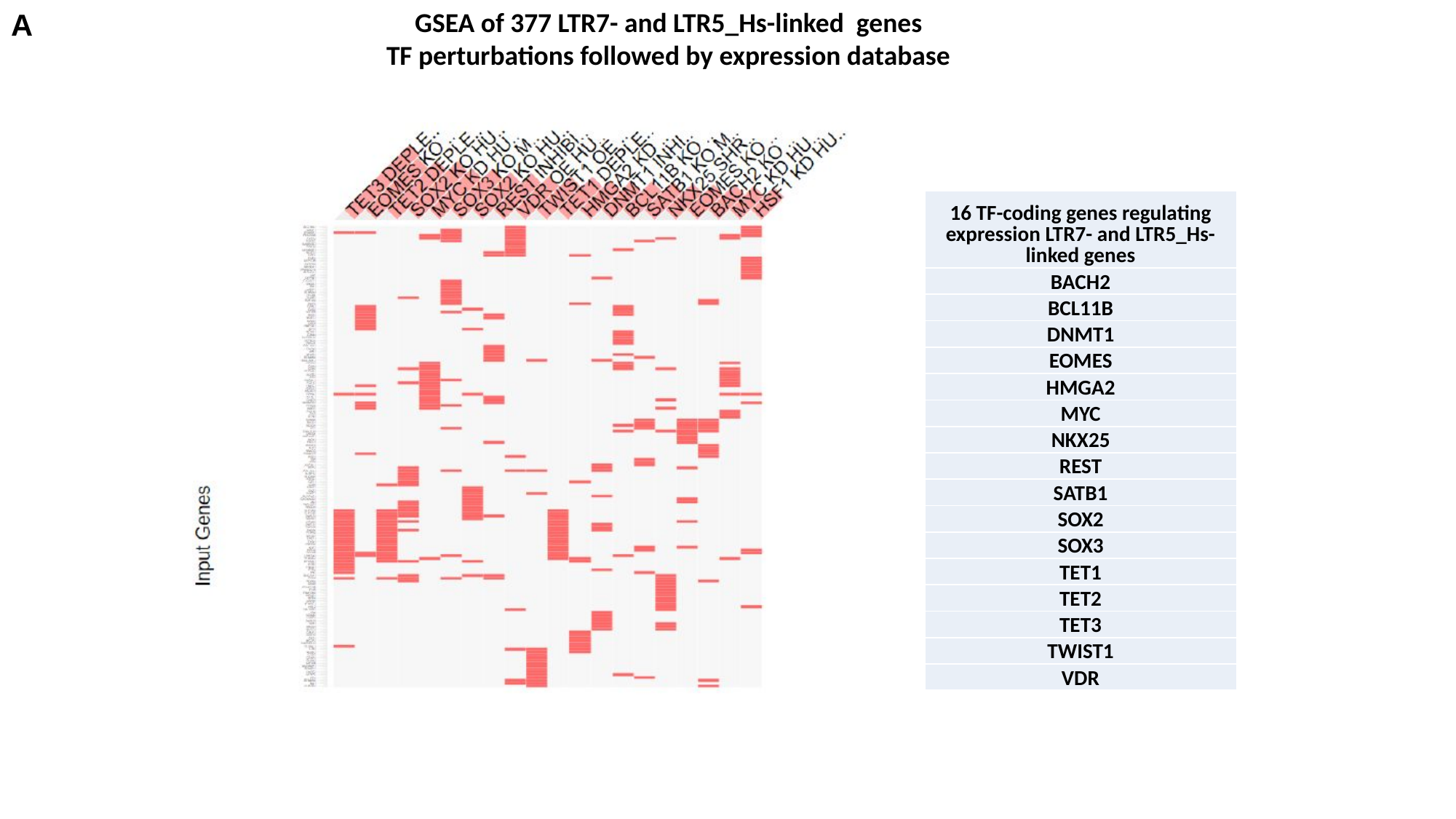

A
GSEA of 377 LTR7- and LTR5_Hs-linked genes
TF perturbations followed by expression database
| 16 TF-coding genes regulating expression LTR7- and LTR5\_Hs-linked genes |
| --- |
| BACH2 |
| BCL11B |
| DNMT1 |
| EOMES |
| HMGA2 |
| MYC |
| NKX25 |
| REST |
| SATB1 |
| SOX2 |
| SOX3 |
| TET1 |
| TET2 |
| TET3 |
| TWIST1 |
| VDR |

## Slide 3
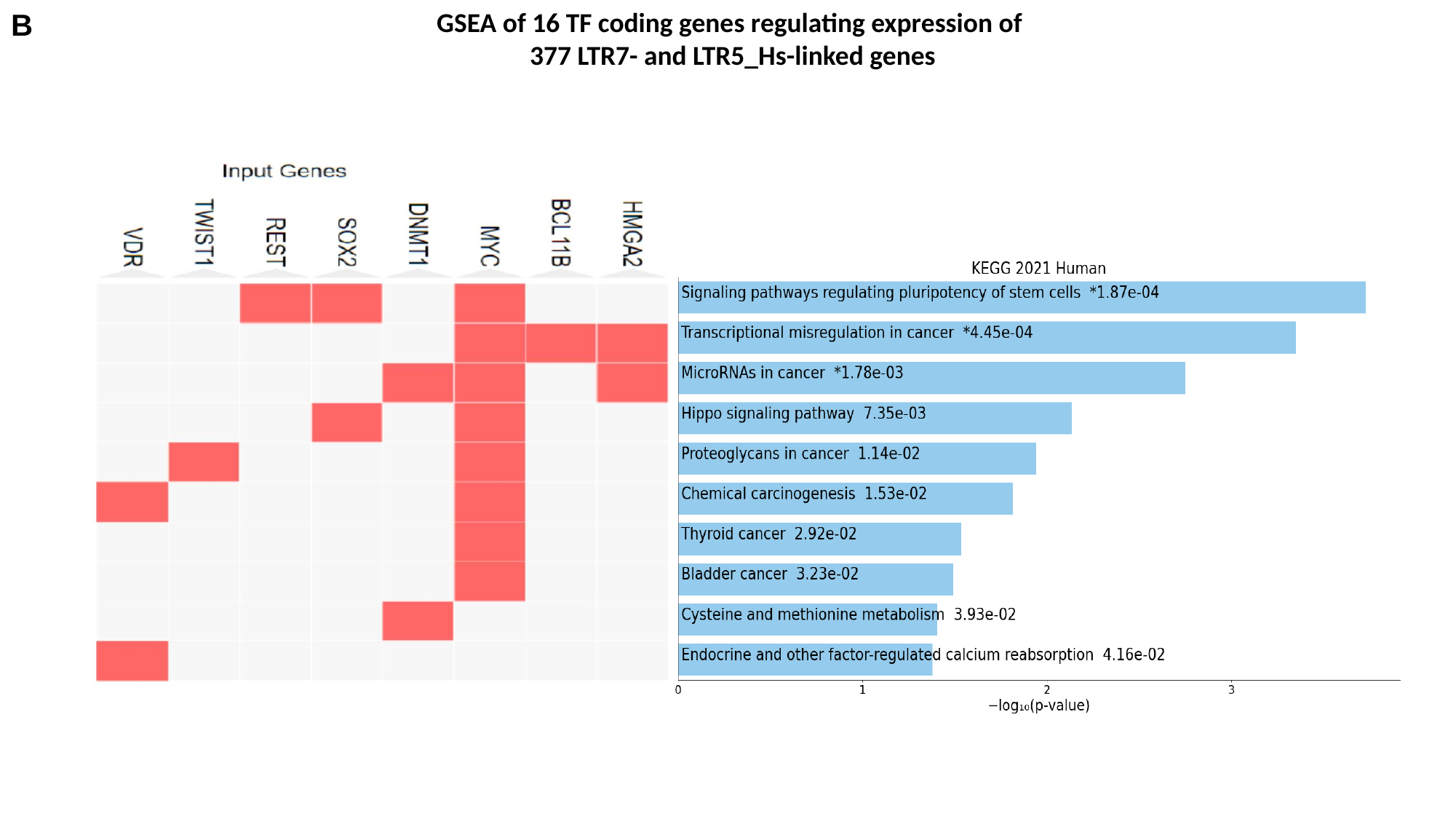

B
GSEA of 16 TF coding genes regulating expression of
377 LTR7- and LTR5_Hs-linked genes

## Slide 4
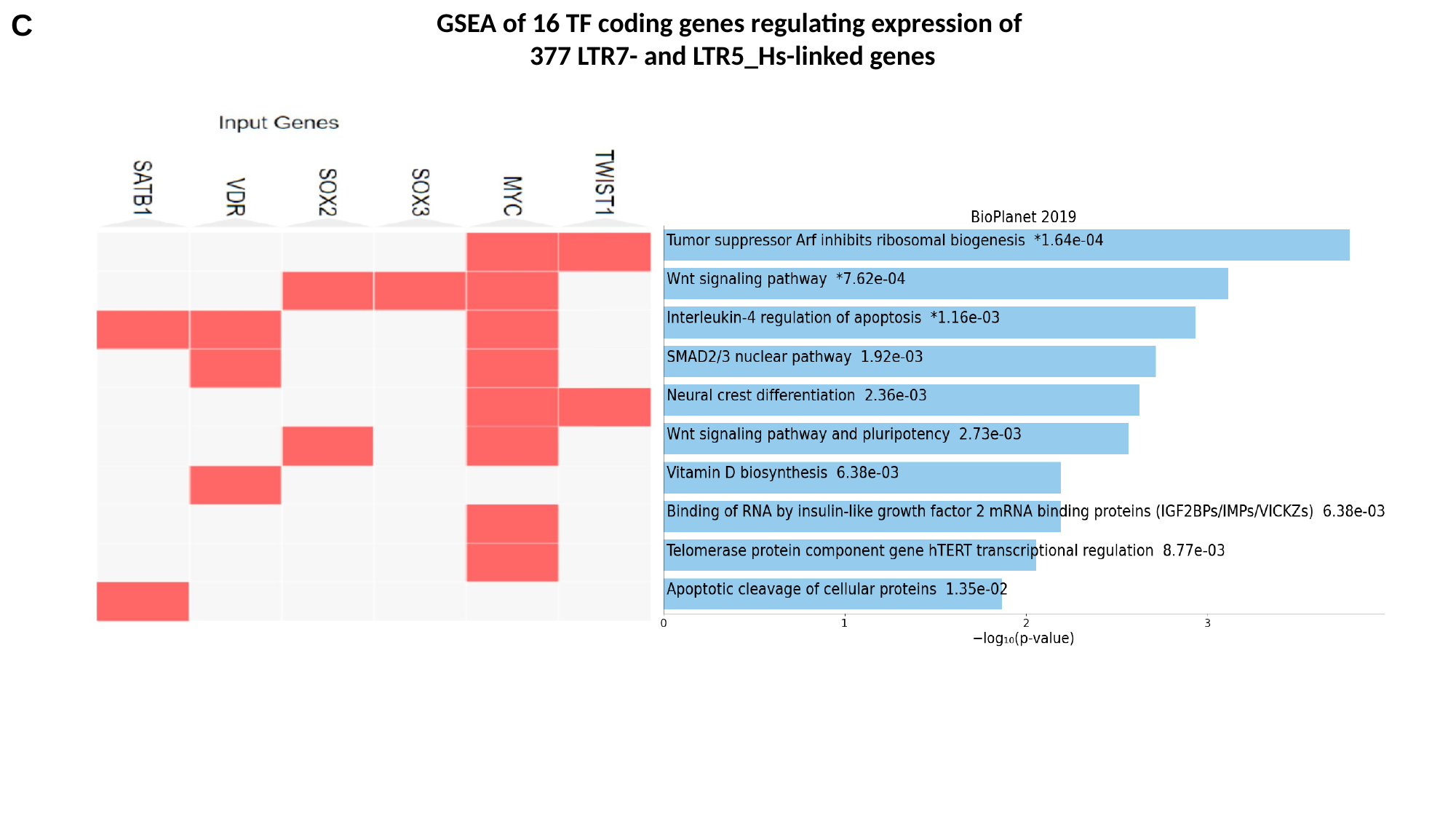

C
GSEA of 16 TF coding genes regulating expression of
377 LTR7- and LTR5_Hs-linked genes

## Slide 5
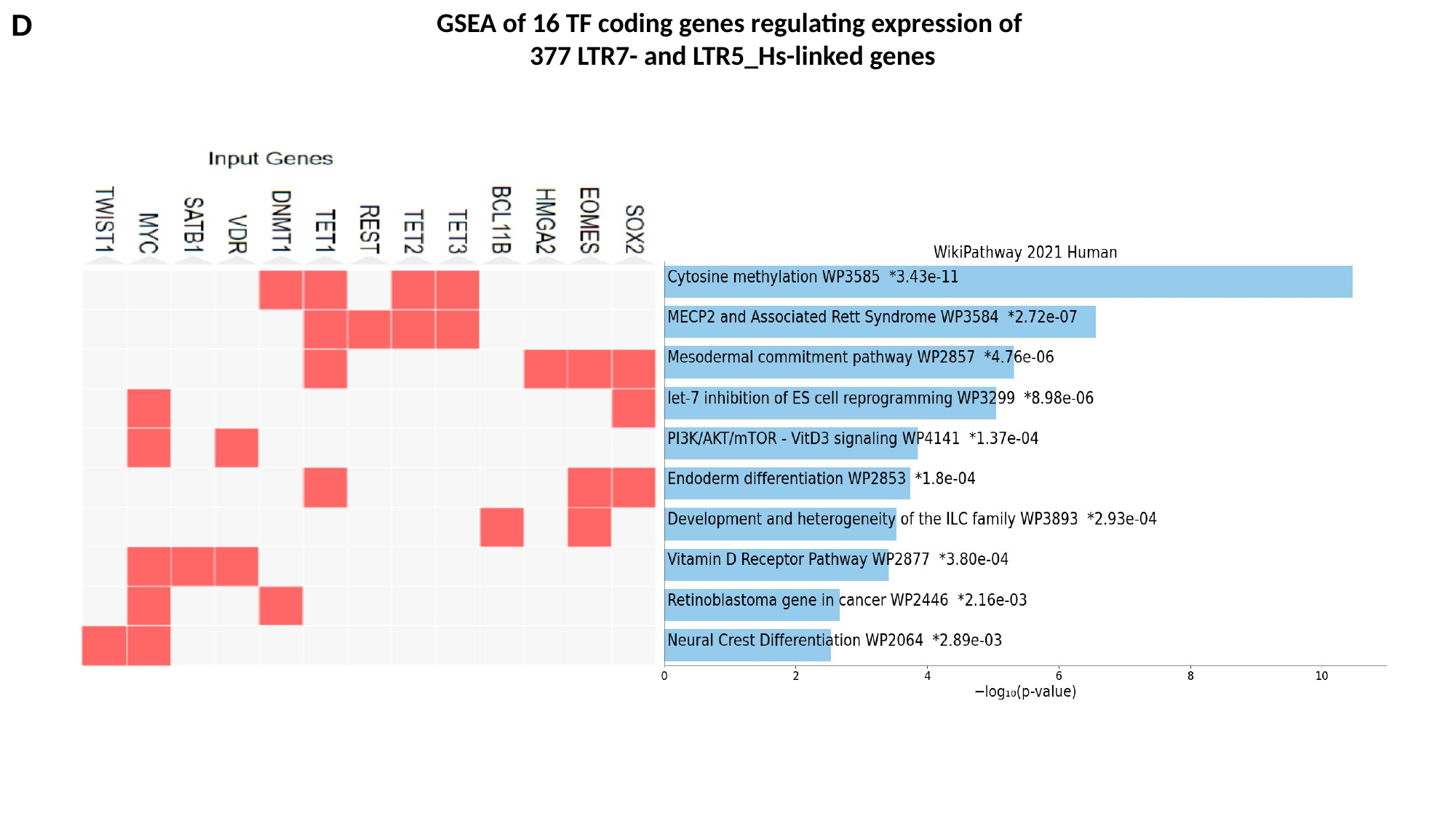

D
GSEA of 16 TF coding genes regulating expression of
377 LTR7- and LTR5_Hs-linked genes

## Slide 6
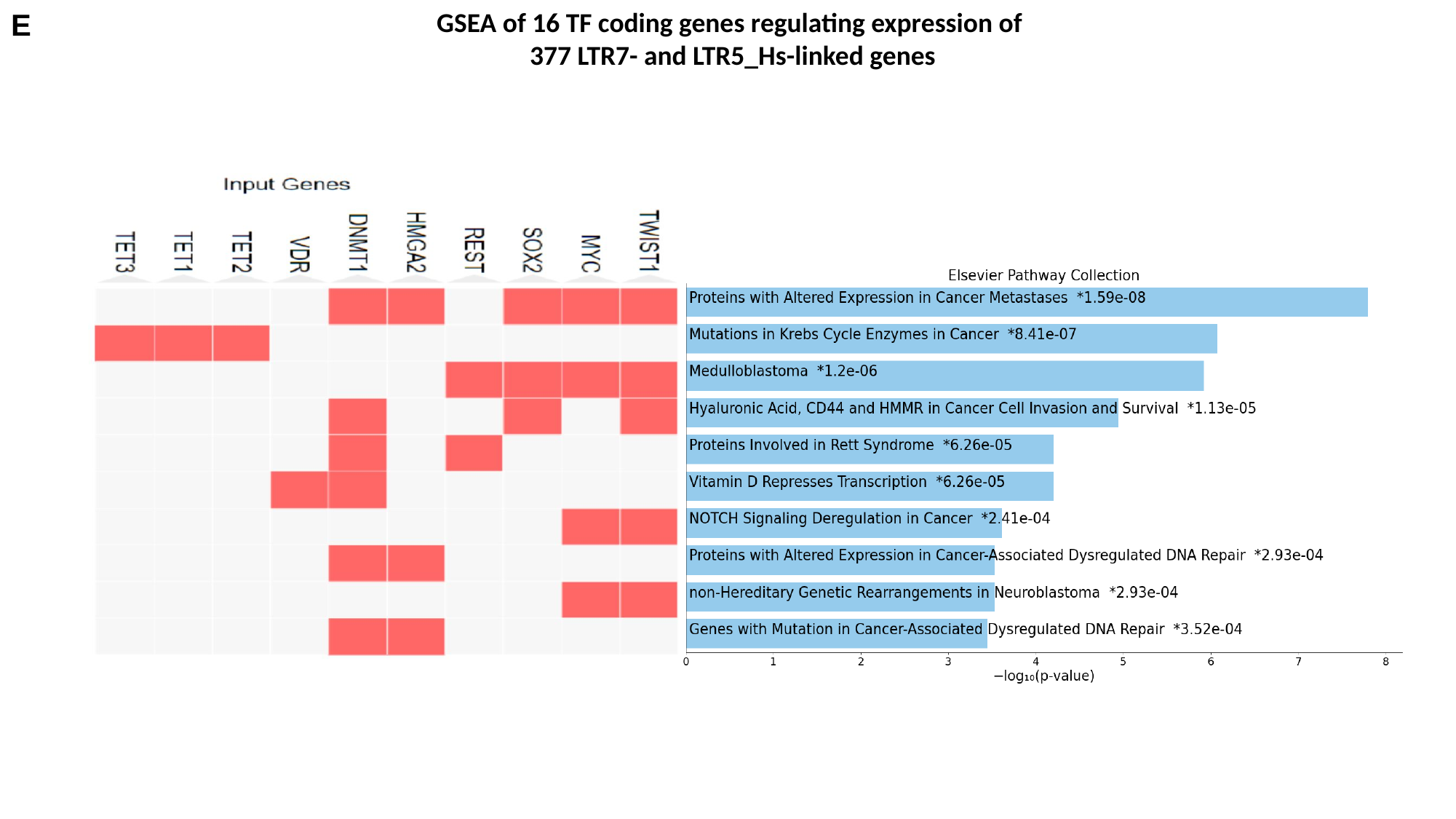

E
GSEA of 16 TF coding genes regulating expression of
377 LTR7- and LTR5_Hs-linked genes

## Slide 7
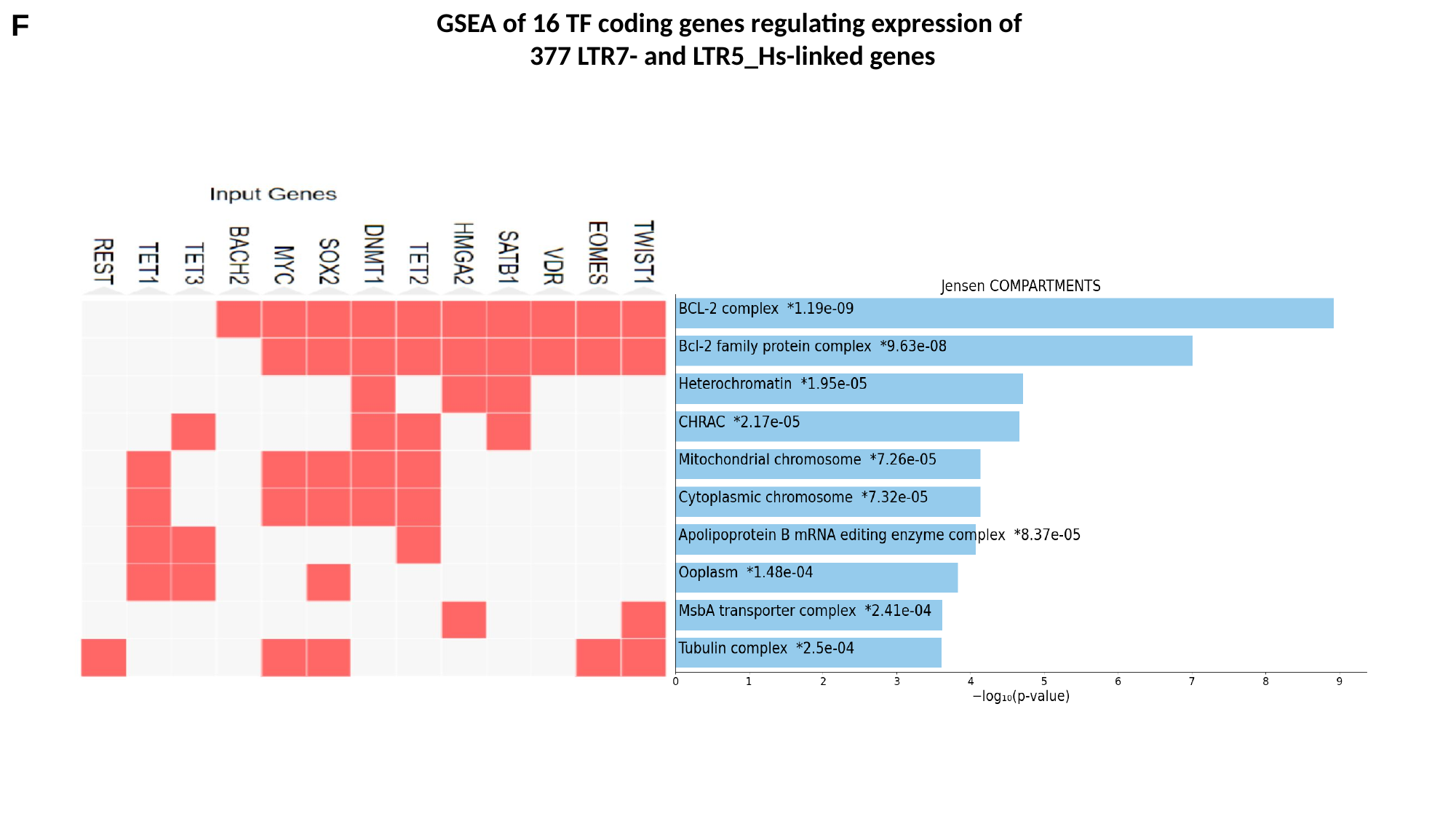

F
GSEA of 16 TF coding genes regulating expression of
377 LTR7- and LTR5_Hs-linked genes

## Slide 8
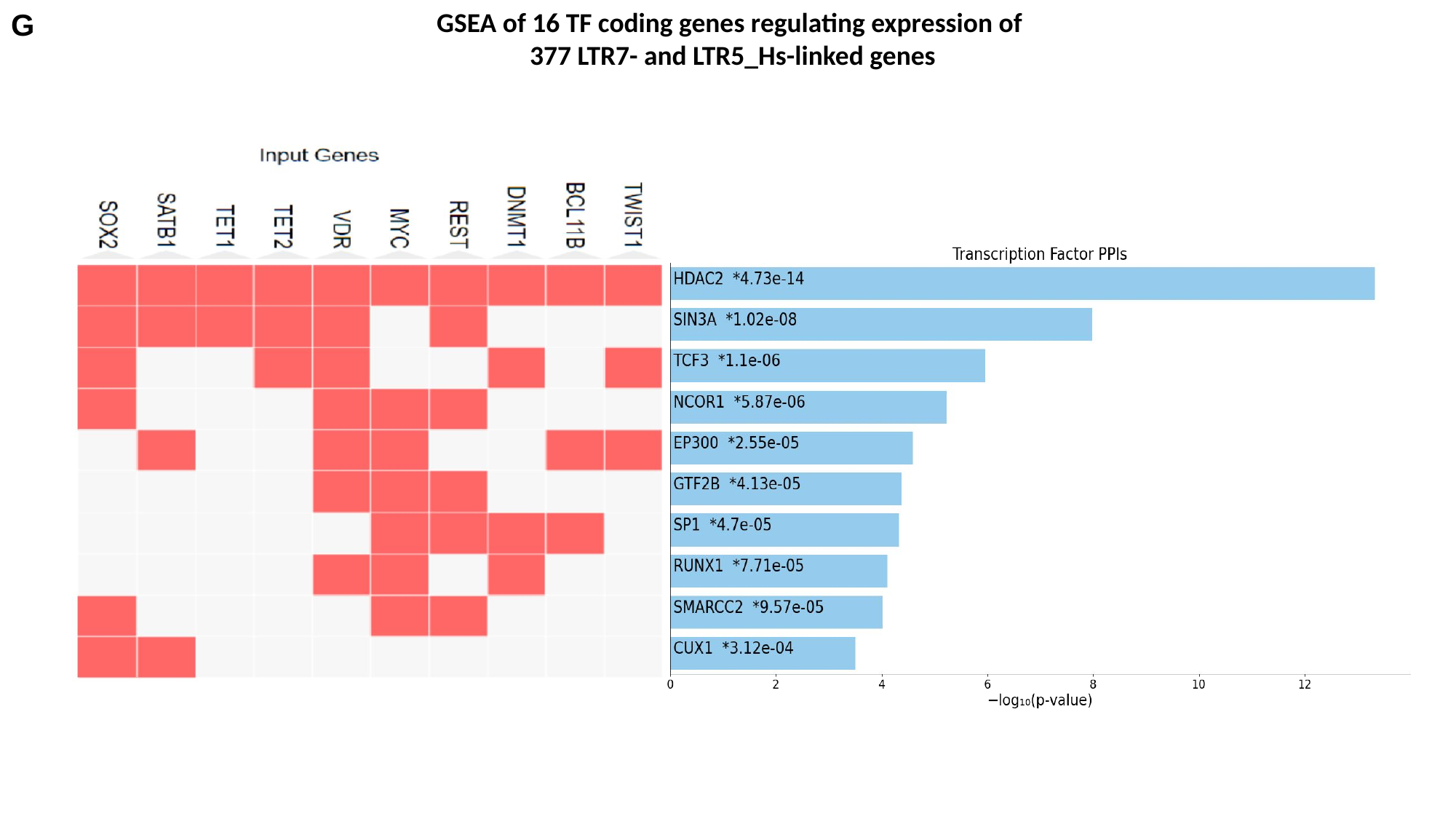

G
GSEA of 16 TF coding genes regulating expression of
377 LTR7- and LTR5_Hs-linked genes

## Slide 9
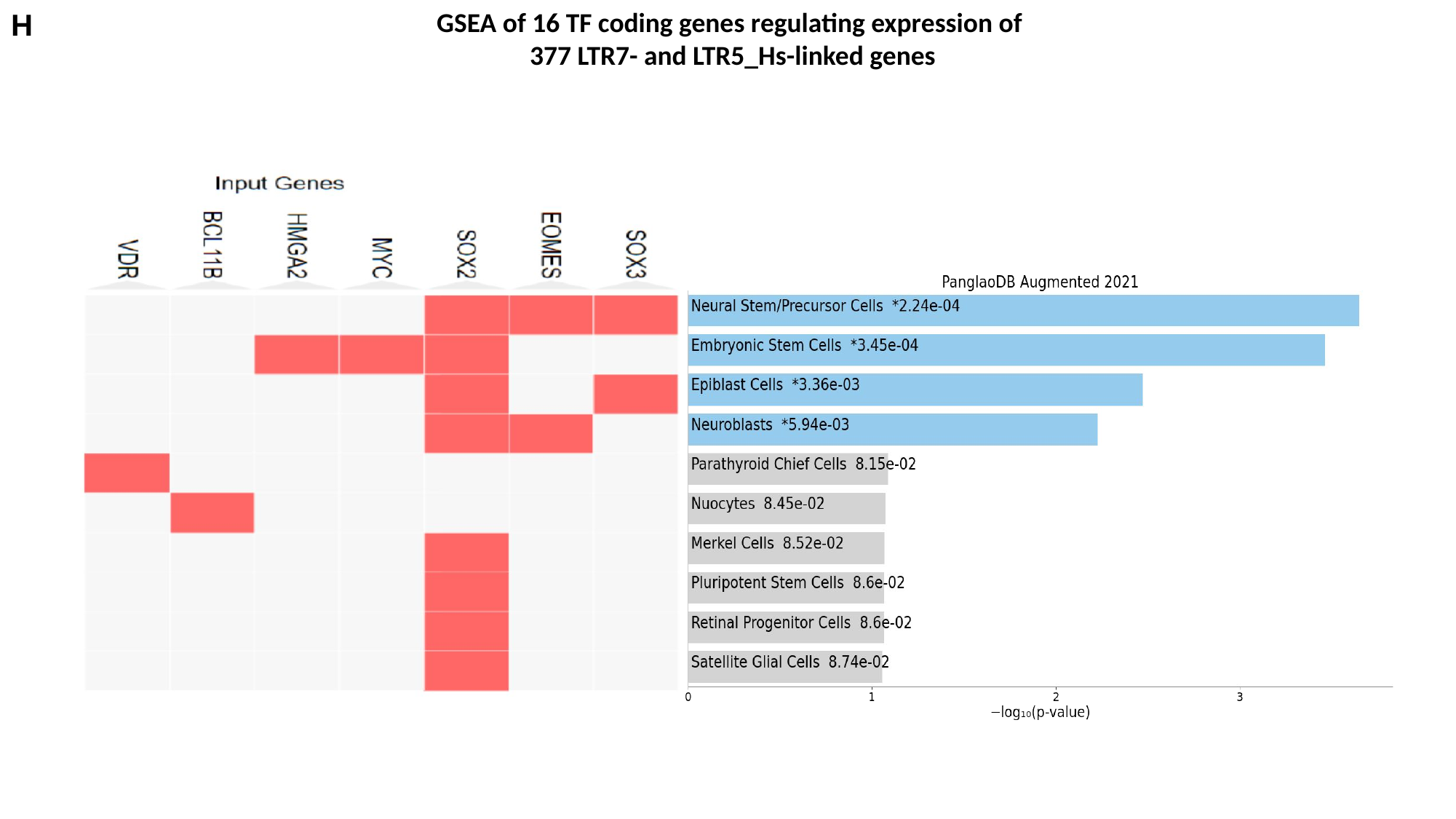

H
GSEA of 16 TF coding genes regulating expression of
377 LTR7- and LTR5_Hs-linked genes

## Slide 10
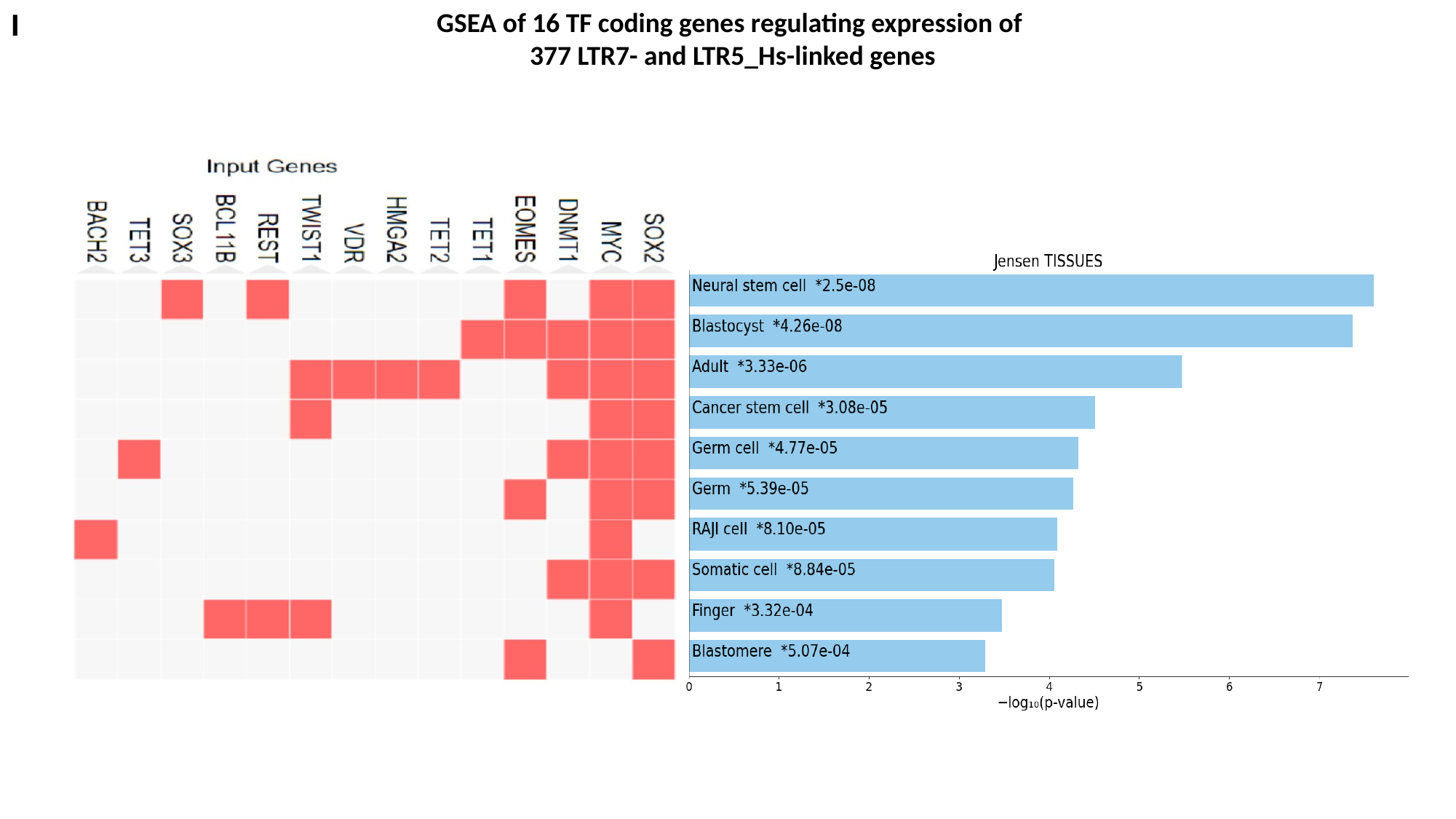

I
GSEA of 16 TF coding genes regulating expression of
377 LTR7- and LTR5_Hs-linked genes

## Slide 11
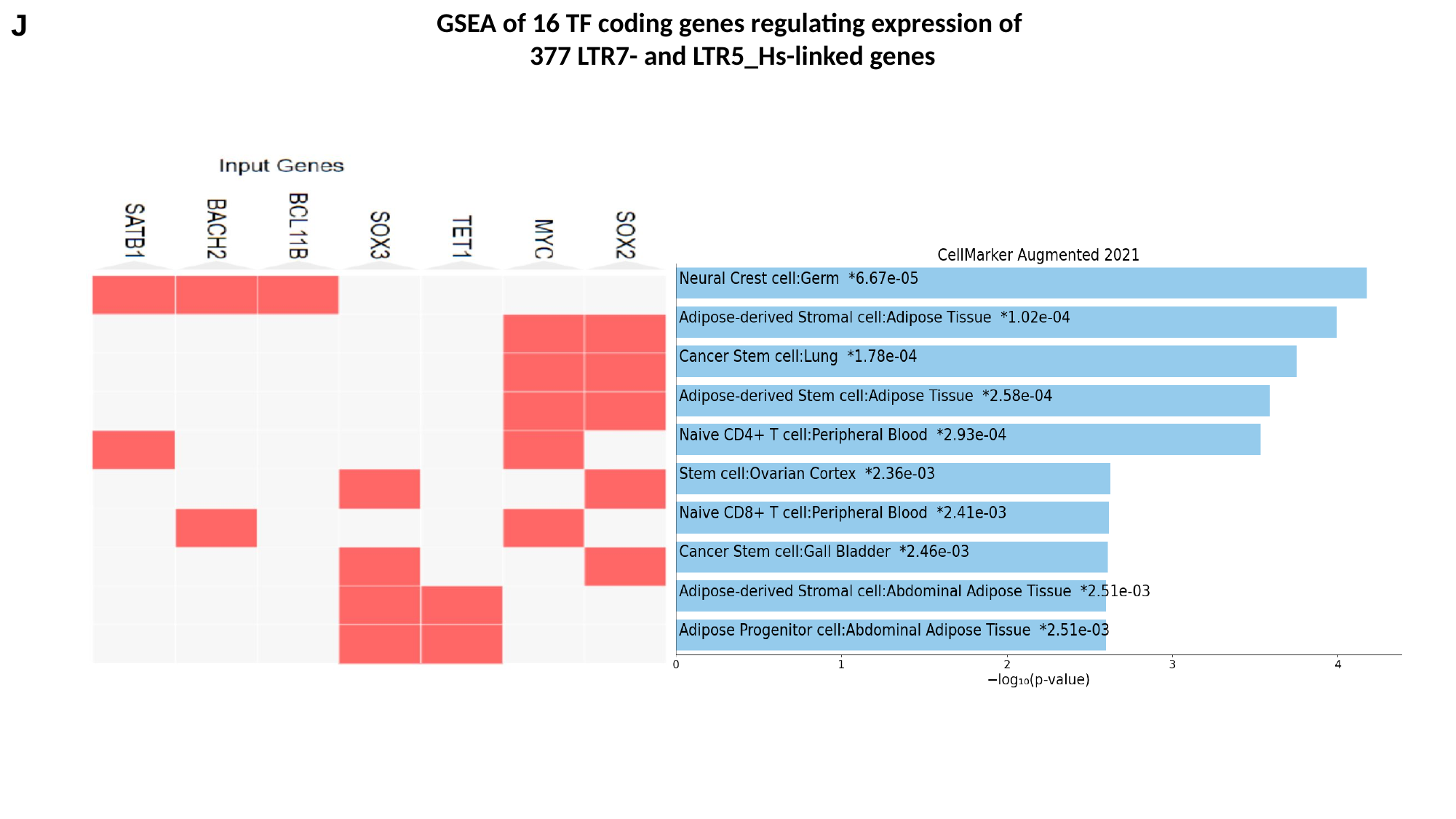

J
GSEA of 16 TF coding genes regulating expression of
377 LTR7- and LTR5_Hs-linked genes

## Slide 12
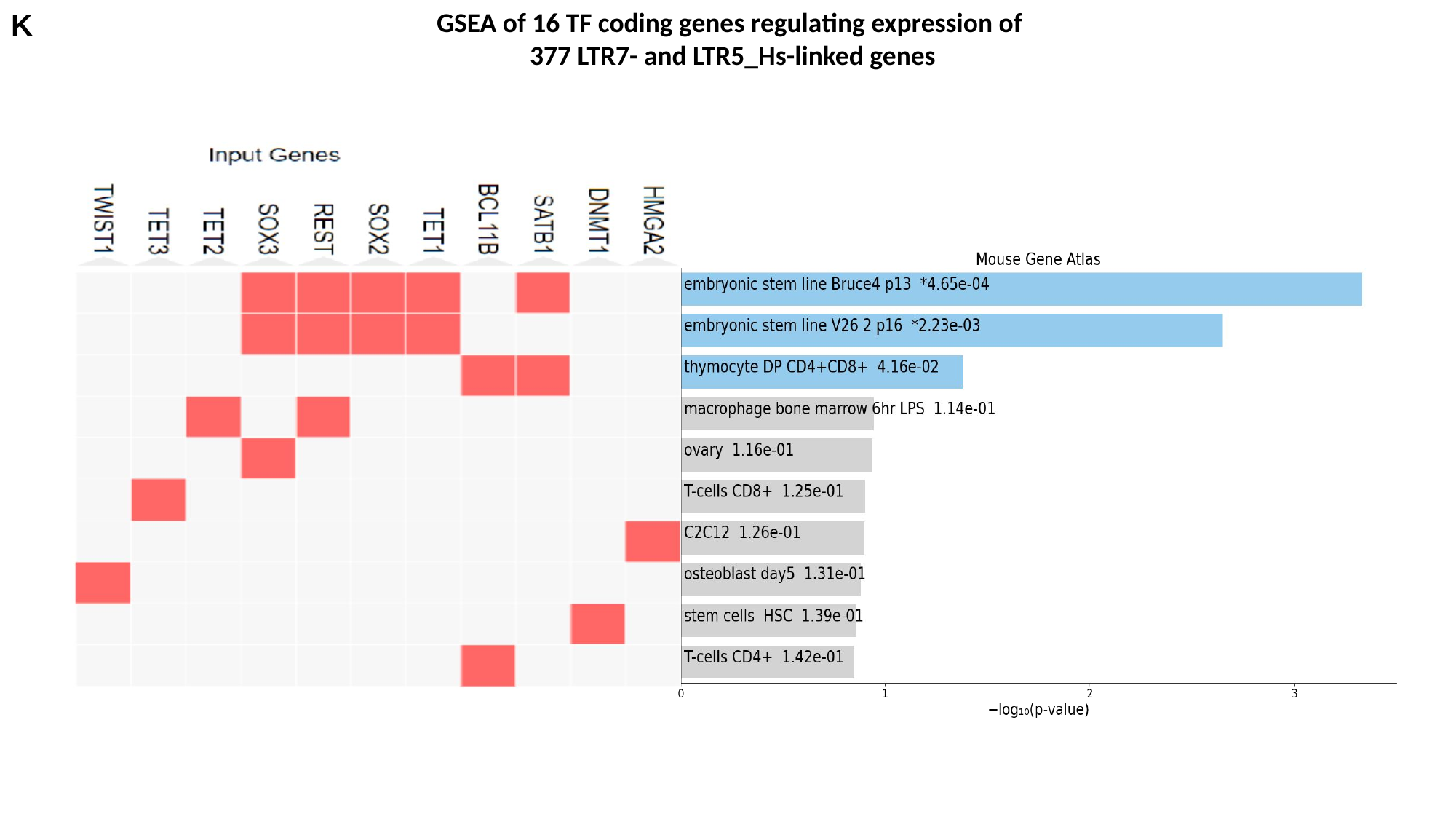

K
GSEA of 16 TF coding genes regulating expression of
377 LTR7- and LTR5_Hs-linked genes

## Slide 13
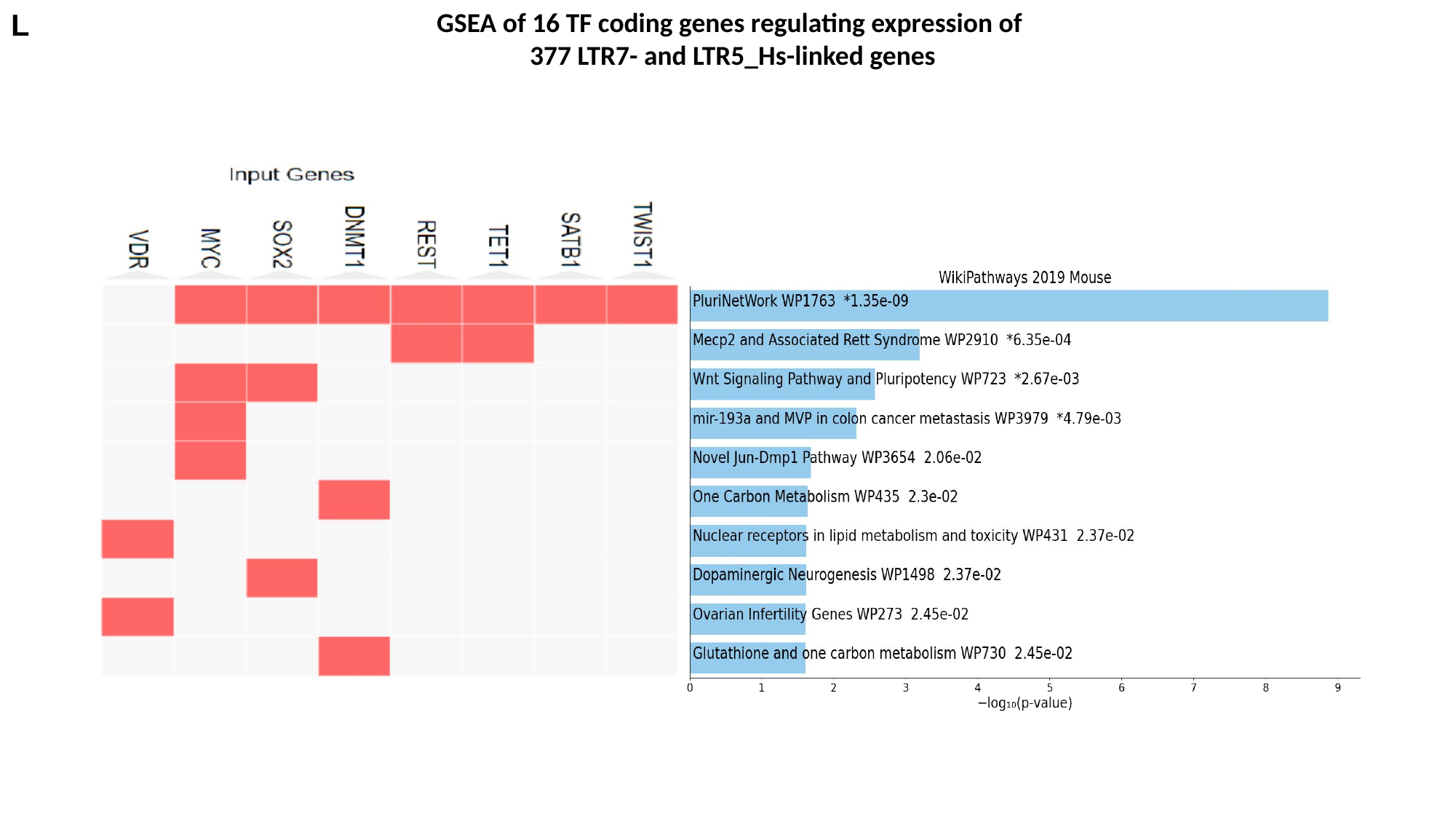

L
GSEA of 16 TF coding genes regulating expression of
377 LTR7- and LTR5_Hs-linked genes

## Slide 14
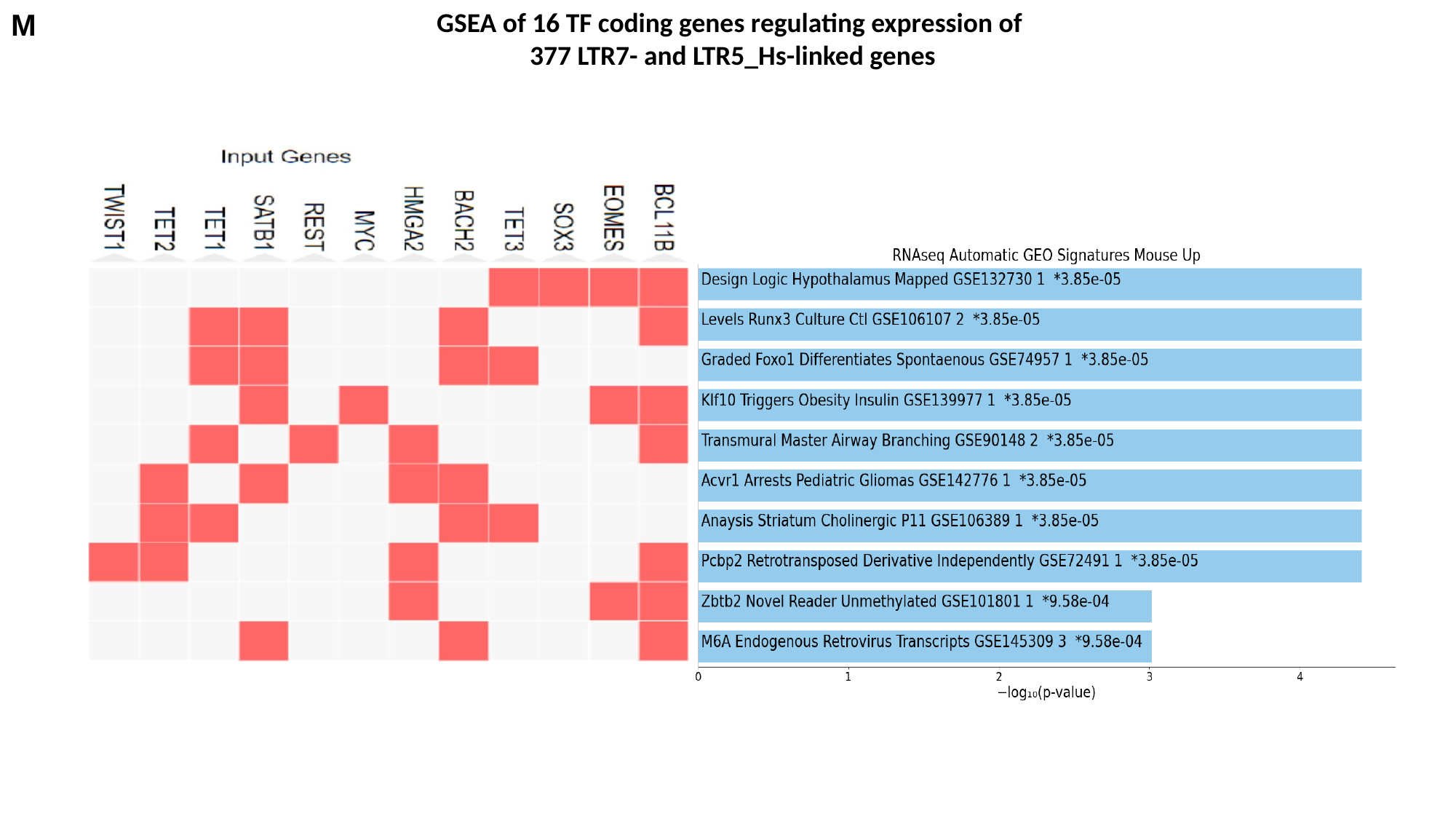

M
GSEA of 16 TF coding genes regulating expression of
377 LTR7- and LTR5_Hs-linked genes
